# Delirium‐associated medication in people at risk: A systematic update review, meta‐analyses, and GRADE‐profiles

**DOI:** 10.1111/acps.13505

**Published:** 2022-10-11

**Authors:** Michael Reisinger, Eva Z. Reininghaus, Johanna De Biasi, Frederike T. Fellendorf, Daniela Schoberer

**Affiliations:** ^1^ Department of Psychiatry & Psychotherapeutic Medicine Medical University of Graz Graz Austria; ^2^ Department of Neurology Klinik Donaustadt Vienna Austria; ^3^ Institute of Nursing Science Medical University of Graz Graz Austria

**Keywords:** deliriogenic, delirium, drug associated delirium, medication, meta‐analysis, systematic review

## Abstract

**Background:**

Drug‐associated delirium is a common but potentially preventable neuropsychiatric syndrome associated with detrimental outcomes. Empirical evidence for delirium‐associated medication is uncertain due to a lack of high‐quality studies. We aimed to further investigate the body of evidence for drugs suspected to trigger delirium.

**Methods:**

A systematic update review and meta‐analyses of prospective studies presenting drug associations with incident delirium in adult study populations was conducted. Two authors independently searched MEDLINE, PsycINFO, Embase, and Google Scholar dated from October 1, 2009 to June 23, 2020, after screening a previous review published in 2011. The most reliable results on drug‐delirium associations were pooled in meta‐analyses using the random‐effects model. Quality of evidence was assessed using the GRADE‐approach. This study is preregistered with OSF (DOI https://doi.org.10.17605/OSF.IO/4PUHY).

**Results:**

The 31 eligible studies, presenting results for 24 medication classes were identified. Meta‐analyses and GRADE level of evidence ratings show no increased delirium risk for Haloperidol (OR: 0.96, 95% CI 0.72–1.28; high‐quality evidence), Olanzapine (OR: 0.25, 95% CI 0.15–0.40), Ketamine (OR: 0.72, 95% CI 0.35–1.46) or corticosteroids (OR: 0.69, 95% CI 0.32–1.50; moderate quality evidence, respectively). Low‐level evidence suggests a three‐fold increased risk for anticholinergics (OR: 3.11, 95% CI 1.04–9.26). Opioids, benzodiazepines, H_1_‐antihistamines, and antidepressants did not reach reliable evidence levels in our analyses.

**Conclusion:**

We investigated the retrievable body of evidence for delirium‐associated medication. The results of this systematic review were then interpreted in conjunction with other evidence‐based works and guidelines providing conclusions for clinical decision‐making.

**Summations**

- Haloperidol, olanzapine, corticosteroids, and ketamine do not exhibit increased risk of delirium (moderate to high quality of evidence).
- There is insufficient evidence for the use of antipsychotics, corticosteroids, or ketamine as pharmacological prevention or treatment.
- Anticholinergics, opioids (especially meperidine), benzodiazepines, H_1_‐antihistamines (especially diphenhydramine), and polypharmacy might trigger delirium, but underlying evidence remains low.

**Limitations**

- Meta‐analysis results deriving from observational studies do not reach reliable evidence levels. Single study results should be interpreted with caution.
- Methodological differences in the studies included (heterogeneous clinical settings, broad ranges of sample sizes/delirium rates, lack of à priori power calculations with frequent low‐precision estimations, inconsistent covariates accounted for in statistical analyses) limited the certainty of evidence.

## 1 INTRODUCTION

Delirium is a serious and common neuropsychiatric disorder that is predominantly reported in elderly patients.^1^ Incidence rates vary from 9% in general medical to 82% in intensive care unit (ICU) settings.^2^ Pathogenesis is highly complex, but mainly affected by predisposing vulnerability and precipitating factors (triggers).^2^ Predisposing risk factors–including increased age, cognitive impairment, frailty, illness severity, co‐ and multi‐morbidity–are not modifiable while there are potentially preventable precipitating factors such as dehydration, electrolyte and metabolic disturbances, or the use of medication with deliriogenic potential.^3^ Even a relatively mild precipitating risk factor (e.g., a newly prescribed sedative) can trigger delirium, particularly in people already at risk of delirium (e.g., elderly patients with dementia and multiple comorbidities).^4^ Drugs may (theoretically) interact with major predisposing risk factors in multiple ways; age‐related changes (e.g., increase in total body fat, decrease in lean body mass/water, decrease in albumin), dementia‐related changes (e.g., decreased cholinergic neurotransmission or impaired blood–brain barrier), and pharmacokinetic consequences of multimorbidity (e.g., altered metabolism/excretion of drugs in hepatic or renal insufficiency; decrease in cytochrome P450 enzyme system) might increase the risk of delirium with many drugs.^5^ Medication as a single factor is described as accounting for 12%–39% of all delirium cases in the elderly.^5^ Since no (pharmacological) treatment approach has yet been able to significantly reduce mortality or cognitive decline, the main clinical focus lies in delirium prophylaxis and the avoidance of precipitating factors.^6^, ^7^

Narrative reviews suggest that benzodiazepines, (tricyclic) antidepressants, opioids, and anticholinergics are suspected to have deliriogenic potential.^3^, ^5^ The evidence, however, is mostly based on pathophysiological hypotheses (esp. decrease in cholinergic transmission, hyper‐dopaminergic state, alterations in γ‐aminobutyric acid and noradrenaline), or empirically on case reports and series.^6^, ^8^ A lack of randomized controlled trials (RCTs) and well‐designed up‐to‐date reviews raises the question of how reliable data on drug‐associated delirium is. In particular, the evidence of associations with specific pharmacological agents with anticholinergic properties (e.g., first‐generation H_1_‐antihistamines) is inconsistent and conflicting.^8^, ^9^, ^10^ Even the National Institute for Health and Care Excellence (NICE) delirium guidelines (updated 2019) do not provide recommendations on avoiding certain medications due to a lack of high‐quality studies.^11^

The aim of this systematic review is to update and comprehensively assess the certainty of evidence for drug‐delirium associations, and to conduct meta‐analyses for delirium with specific medication (classes). A systematic literature search is conducted to update a previous review from 2011.^9^

## 2 MATERIALS AND METHODS

This work is based upon the recommendations of the Cochrane Handbook for Systematic Reviews of Interventions^12^ and was guided by the PRISMA statement.^13^ The study protocol was preregistered in the Open Science Framework.

### 2.1 Design and eligibility criteria

The systematic search required RCTs and prospective observational studies (OS) (prospective cohort [PC] and nested case–control [CC]) with predefined eligibility criteria assessing medication associations with incident delirium in adult study populations. Retrospective study designs, case‐reports, and studies that failed to present exploratory statistics were considered to provide a very low level of evidence, and therefore excluded.^14^ Eligibility criteria for delirium assessment were DSM‐ or ICD‐criteria (all versions) or assessment instruments validated against the latter. There were no restrictions for medication exposure, with the only exception being anticholinergics without the further classification of agents and anticholinergic drug scales (inconsistent comparability).^15^ A comprehensive table of applied eligibility criteria can be found in the Appendix S1.

### 2.2 Search methods

A search strategy using Medical Subject Headings (MeSH) and free text search terms was developed in order to systematically screen the MEDLINE (via PubMed), EMBASE, and PsycInfo databases. A complementary search on Google Scholar and screening of reference lists was conducted to extend the scope. The search strategy combined three search strings, reflecting (i) delirium as the dependent variable, (ii) medication exposure as the independent variable, and (iii) focus on elderly people (> 65 years old), by using the Boolean operator “AND.” Eligibility criteria required adult study populations (exclusion of <18 years old patients) with a focus on older patients. English‐, German‐ and French‐language journal articles were included if published between October 1, 2009–to update the previous systematic review^9^ – and June 23, 2020 (for more details see the Appendix S2).

The literature search was initially conducted by screening the review by Clegg and Young^9^ for eligible studies. Systematic electronic database screening on titles, abstracts, and full texts was then conducted separately by two independent reviewers (RM and DBJ). Possible disagreement was resolved by consensus, or a third independent author (SD).

### 2.3 Data extraction

Data collection of included articles was undertaken by RM while DBJ checked for the accuracy of extracted data. We focused especially on delirium assessment, medication exposure, and statistical approach to ascertain reliable data and assess the possible risk of bias. Adjusted and unadjusted effect measures, as well as raw data, were collected.

### 2.4 Quality appraisal

RCTs were appraised using the Cochrane risk of bias tool (RoB).^16^ OS were assessed using the Newcastle‐Ottawa‐Scale (NOS),^17^ in accordance with the previous review.^9^ We adapted the scale: *age* was defined as a primary confounder, and *dementia* and *comorbidity/illness severity* as joint secondary confounders. A score of ≥7 was regarded as indicative of sufficient bias control. There was a risk of bias, if more than one point in a single category was subtracted.

### 2.5 Summary measures

Meta‐analyses were conducted if there were at least two studies investigating the same medication. These were pooled using the random‐effects model.^18^

Pooled odds ratios (ORs) with 95% confidence intervals (CI) were calculated for dichotomous outcomes (Cochran–Mantel–Haenszel test or generic inverse‐variance method). Statistical heterogeneity was calculated using the χ2 test (with statistical significance at *p* < 0.05) and the I^2^ statistic.^19^ We used the Review Manager (RevMan) software, Version 5.4^20^ to compile meta‐analyses. Relative effect sizes were translated to absolute ones using either the GRADEpro^21^ software or by hand with the formula recommended by the GRADE group.^22^

In addition, we conducted sensitivity analyses to evaluate the robustness of effects. Pooled effect sizes from higher quality studies and multivariable statistical approaches were therefore compared to those from all included studies or univariable analyses.

### 2.6 Certainty in evidence

Certainty in the body of evidence was assessed using the GRADE‐approach^23^ which provides a structured and transparent way of rating the quality of evidence across studies by evaluating the risk of bias, inconsistency (or heterogeneity) of results, indirectness according to the research question, and the imprecision of (pooled) effect estimates.^24^ We focused on sufficient statistical power to evaluate imprecision. The optimal information size (OIS) was estimated and used as a surrogate in accordance with GRADE recommendations.^25^ The GRADE‐approach classifies the quality of evidence as high, moderate, low, and very low.

## 3 RESULTS

### 3.1 Study selection

**FIGURE 1:**
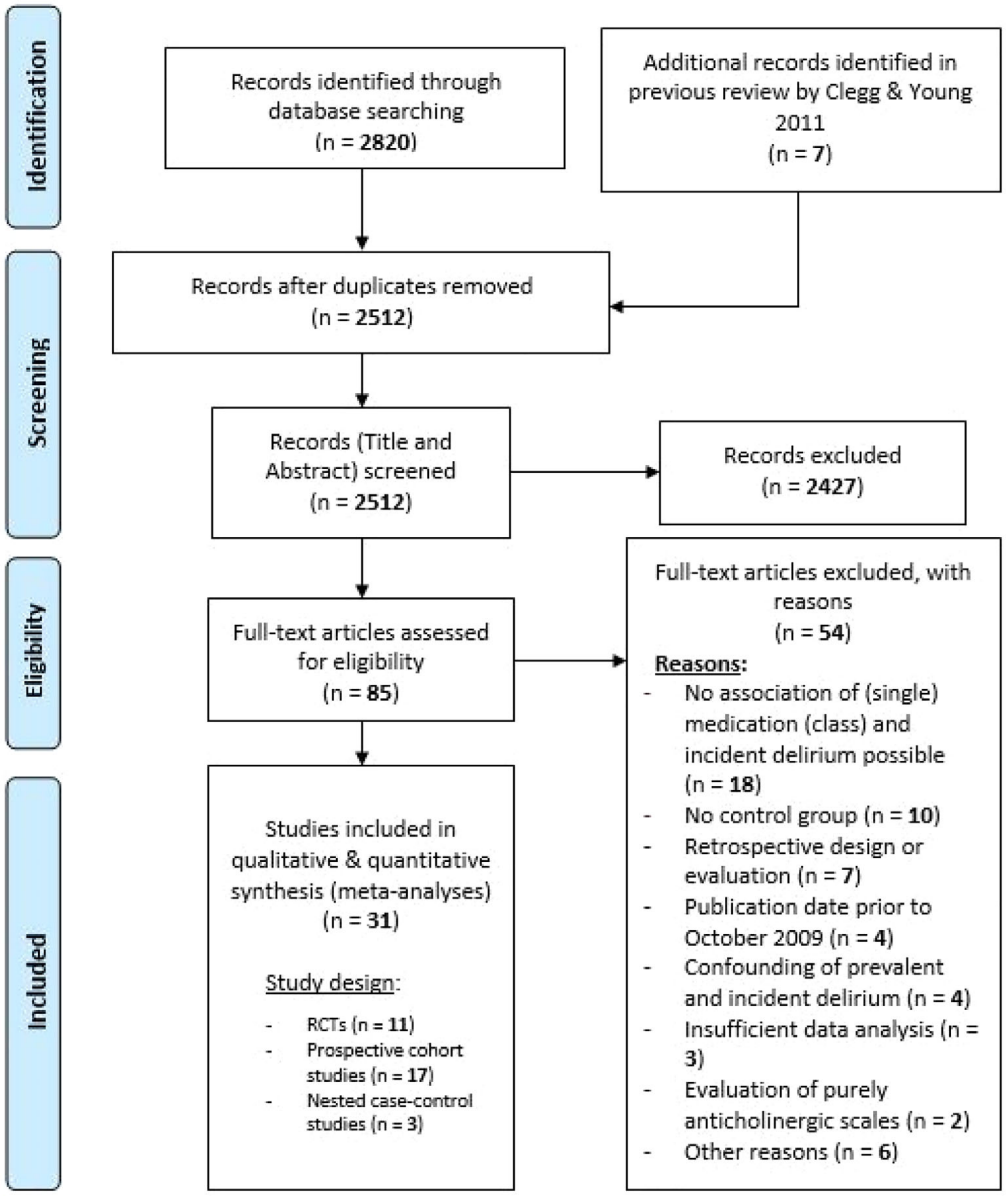
PRISMA flowchart

The systematic database search yielded 2820 articles in total. After title/abstract screening, 2427 studies were excluded (research question or inclusion criteria not met). In full text screening, the eligibility criteria (see Appendix S3) was applied to 85 studies, after which 54 works needed to be excluded. The 31 studies met eligibility criteria (11 randomized controlled trials [RCTs],^26^, ^27^, ^28^, ^29^, ^30^, ^31^, ^32^, ^33^, ^34^, ^35^, ^36^ 17 PC studies [PCs]^37^, ^38^, ^39^, ^40^, ^41^, ^42^, ^43^, ^44^, ^45^, ^46^, ^47^, ^48^, ^49^, ^50^, ^51^, ^52^, ^53^ and 3 prospective case–control studies [CCs]^54^, ^55^, ^56^ of which seven works^28^, ^41^, ^42^, ^43^, ^44^, ^45^, ^56^ were included from the previous review).^9^ The detailed selection process, describing the in‐ and exclusion (with reasons) of retrieved studies, is outlined in Figure 1.

### 3.2 Study characteristics

Comprehensive study characteristics are presented in Table 1.

**TABLE 1:** Study characteristics

Study ID, Year	CEBM level of evidence	Setting	Sample size (n)/delirium cases (n, %)	Age (mean, SD)	Patients with dementia or cognitive impairment	Diagnostic criteria and assessment	Medication exposure			Statistical approach/analysis and factors adjusted for
							Source of medication data, method of data extraction	Medication (class), type and window of medication exposure	Ascertainment of medication exposure prior to delirium	
Randomized controlled trials (RCTs); *n* = 11										
Hongyu et al., 2019^26^	2	Surgical (thoracoscopic surgery)	90/28 (31.1%)	72.4 (5.8)^d^	Excluded	Delirium assessment pre‐OP and on POD 1, 4 & 7 using CAM; not stated who assessed patients; no information on blinding	Source and method of medication data extraction not specified	Anticholinergics: intervention with either 0,01 mg/kg i.m. PHC, atropine or placebo 5 min before surgery	Yes	Delirium rates were compared using chi‐square test; analysis type (PP or ITT) not specified
Larsen et al., 2010^27^	2	Surgical (knee/hip replacement)	400/110 (27.5%)	73.4 (6.1)^d^	Excluded	Daily delirium assessment from POD 1–8 by research assistants (MMSE, DRS‐R‐98), nurses (CAM) & blinded clinical psychologists (DSM‐III‐R‐criteria)	Medication data from randomization allocation, extracted by research staff after unblinding	Antipsychotics: intervention with either 5 mg p.o. olanzapine pre‐ and post‐OP (10 mg in total) or placebo for delirium prevention	Yes	PP MRM including all variables with univariate *p* values <0.10
Schrijver et al., 2018^29^	2	Mixed medical & surgical °	242/41 (16.9%)	83.5 (6.3)	Excluded	Daily delirium assessment by trained clinicians & investigators based on structured patient observations, medical notes & nursing records reviewing using DOSS, DSM‐IV & DRS‐R‐98	Medication data from randomization allocation, extracted by research staff after unblinding	Antipsychotics: intervention with either 1 mg p.o. haloperidol BID or placebo for 7 days for delirium prevention	Yes	ITT
Van den Boogaard et al., 2018^30^	2	Mixed^a^ ICU	1789/616 (33.4%)	66.6 (12.6)	Excluded	Daily delirium screening in the ICU (at least twice daily) as part of daily ICU care using CAM‐ICU, ICDSC (in the ICU) & DOSS (on normal ward); not specified who assessed patients	Medication data from randomization allocation, extracted by research staff after unblinding	Antipsychotics: intervention with either 1 mg, 2 mg i.v. haloperidol or placebo TID for 28 days, until discharge or delirium for delirium prevention	Yes	ITT & PP
Wang et al., 2012^31^	2	ICU (noncardiac surgery)	457/88 (19.3%)	74.0 (5.8)^d^	Partly included^i^	Daily delirium assessment by research members (not involved in clinical care) for 7 days after surgery using CAM‐ICU; no delirium assessment on admission	Medication data from randomization allocation, extracted by research staff after unblinding	Antipsychotics: intervention with either 1.7 mg i.v. haloperidol (0.5 mg bolus +0.1 mg/h for 12 hrs) or placebo for delirium prevention	Not specified	ITT MRM adjusted for variables with a p‐value <0.10
Avidan et al., 2017^32^	2	Surgical (cardiac and noncardiac)	654/128 (19.6%)	70 (7.1)	Not specified^g^	Delirium assessment 2 h post‐OP & twice daily from POD 1–3 by blinded & trained members of the research team using CAM, CAM‐ICU & CAM‐S	Medication data from randomization allocation, extracted by research staff after unblinding	Anesthetics: intervention with either 0.5 mg/kg, 1 mg/kg i.v. ketamine or placebo for delirium prevention	Yes	ITT MRM controlling for known risk factors (ratio of variables to outcomes to 1:10)
Al‐Qadheeb et al., 2016^33^	2	Mixed^a^ ICU (MVP)	68/20 (29.4%)	61.7 (16.9)^d^	Excluded (IQCODE ≥4)	Delirium assessment at admission & daily by bedside nurse (ICDSC), evaluated by investigative team & confirmed by a consulting psychiatrist (DSM‐criteria)	Medication data from randomization allocation, extracted by research staff after unblinding	Antipsychotics: intervention with either 1.0 mg i.v. haloperidol every 6 h (4 mg per day) or placebo until delirium, ICU discharge, 10 days of therapy or an ADE for delirium prevention	Yes	ITT
Clemmesen et al., 2018^34^	2	Surgical (hip fracture surgery)	117/29 (24.8%)	79 (8)^d^	Partly included^i^	Daily delirium assessment using CAM‐S until POD 3 by one study author or trained clinical staff	Medication data from randomization allocation, extracted by research staff after unblinding	Corticosteroids: intervention with either 125 mg i.v. methylprednisolone or placebo pre‐OP for (post‐OP) delirium prevention	Yes	ITT
Sauër et al., 2014^35^	2	Surgical (cardiac surgery)	737/107 (14.5%)	67 (12)^d^	Not specified^g^	Daily delirium assessment by trained research personnel up to POD 4 using CAM & CAM‐ICU, accompanied with chart review of previous 24 h (by bedside nurses as well as neuroleptic use or other surrogates for delirium); no delirium assessment on admission	Medication data from randomization allocation, extracted by research staff after unblinding	Corticosteroids: intervention with either 1 mg/kg i.v. dexamethasone (max. 100 mg) or placebo at the time of induction of anesthesia for delirium prevention	Not specified	ITT MRM adjusted for baseline variables: age, gender, valve surgery, and history of stroke
Perbet et al., 2018^36^	2	Mixed^a^ ICU; MVP)	162/47 (29%)	63 (15)^d^	Not specified^g^	Daily delirium assessment by bedside nurse using CAM‐ICU; no delirium assessment on admission	Medication data from randomization allocation, extracted by research staff after unblinding	Anesthetics: intervention with either 0.2 mg/kg/h i.v. ketamine vs placebo for reduction of opioids (and delirium prevention)	Not specified	PP (no lost to follow up) MRM adjusted for fixed covariables according to univariate results and clinical relevance
Kalisvaart et al.^h^, 2005^28^	2	Surgical (hip surgery)	430/68 (15.8%)	78.71 (6.04)^d^	Partly included^i^	Daily assessment by blinded research team (geriatricians and research nurses not involved in clinical care) from day of admission until discharge using CAM, DSM‐IV criteria, DRS‐R‐98	Medication data extracted from standardized patient record forms by blinded research team	Antipsychotics: intervention with either 1.5 mg p.o. haloperidol) or placebo from day of admission until 3 days post‐OP for delirium prevention	Yes	ITT
Prospective cohort studies (PCs), n = 17										
Burry et al., 2017^37^	3	Mixed ICU^a^	520/260 (50%)	61.7 (16.2)^c^	Included	Daily delirium assessment by research staff with assistance of bedside nursing staff using ICDSC; time frame of delirium assessment not specified	Medication data from pharmacy & medication administration records, extracted (daily) by research staff	ICU medication: benzodiazepines, non‐benzodiazepine sedatives, opioids, antipsychotics & drugs with anticholinergic activity	Yes (24 h or 48 h)	MRM adjusted for: age, APACHE II score on admission, smoking, history of significant alcohol consumption and hypertension, presence of pre‐existing neurologic condition, ICU admission type and mechanical ventilation
Hein et al., 2014^38^	3	Acute geriatric care unit^b^	410/102 (24.9%)	85.51 (1.16)	Included	Delirium assessment within 72 h of admission by geriatrician using CAM; time frame and frequency of delirium assessment not specified	Medication data from patient or caregiver interview & chart review, extracted by geriatrician within 72 h of admission	Baseline medication: polypharmacy (≥6 concurrent drugs)	Not specified	MRM adjusted for: dementia, age, intake of drugs known to induce delirium, severe renal impairment, and source of admission
Schreiber et al., 2014^46^	3	Mixed ICU^a^	330/99 (transitions to delirium); n of delirium cases not stated	51 (IQR 41, 63)^j^	Excluded	Daily delirium assessment by trained research staff using CAM‐ICU; time frame of delirium assessment not specified	Medication data from medication administration records, extracted by research staff	ICU medication: corticosteroids (prednisone‐equivalents), benzodiazepines (midazolam‐equivalents), opioids (morphine‐equivalents)	Yes	MRM adjusted for: demographics (age, race, sex), individual covariates (p ≤ 0.2 in bivariable analysis); predefined baseline covariates (e.g. severity of illness score, ICU covariates, other medication classes)
Wolters et al., 2015^47^	3	Mixed ICU^a^	1.112/535 (48.1%)	60 (16)	Not specified (neurological disorders excluded)	Daily delirium assessment independently by bedside (CAM‐ICU) and research nurse (chart review for delirium symptoms or antipsychotic drug initiation for previous 24 h); time frame of delirium assessment not specified	Medication data from medication administration records; not specified who extracted data	ICU medication: corticosteroids (dexamethasone, fludrocortisone, hydrocortisone, methylprednisolone & prednisone)	Yes	MRM adjusted for: age, corticosteroid use prior to ICU admission, CCI, type of ICU admission, APACHE IV score, length of ICU‐stay, SOFA score, use of mechanical ventilation, presence of inflammation, use of opioids and benzodiazepines
Brown et al., 2016^48^	3	Surgical (spine surgery)	89/36 (40.5%)	75 (IQR 72–78)^j^	Excluded (MMSE score < 15)	Daily delirium assessment for the first 4 postoperative days by trained research assistants using CAM, CAM‐ICU, validated chart review and DRS‐98‐R	Medication data from medical chart review or patient interview, extracted by research assistants	Baseline medication: opioids, benzodiazepines, antidepressants, beta blockers, ACE inhibitors, calcium channel blockers Intra‐OP medication: corticosteroids	Yes	MRM adjusted for à priori covariates: age, functional status, ASA risk score, surgery length, intraoperative red blood cell transfusion, return to the operating room and any complication
Saljuqi et al., 2020^49^	3	Surgical (emergency general surgery)	145/38 (26.2%)	71 (8)	Included	Delirium assessment by investigators using CAM; time frame & frequency of delirium assessment unclear	Medication data from medical chart review, extracted by authors (not further specified)	Baseline medication: polypharmacy (≥ 3 medications)	Not specified	MRM adjusted for: variables significant on the univariable level and confounders: demographics (e.g. age), vital parameters on admission, comorbidities, laboratory parameters, ASA class and diagnosis
Taipale et al., 2012^50^	3	(Cardiac surgical) ICU & nursing units	122/46 (37.7%)	66.8 (9.4)	Excluded	Delirium assessment 12‐18 h post‐OP & daily for 72 h following surgery by trained study nurses using CAM‐ICU & physician record review	Medication data from medical records extracted by study nurses	ICU medication: midazolam PRN‐administration (range 0–83 mg; median 3.0 mg) by ICU nurses (for sedation of ICU patients)	Partially^f^	MRM adjusted for: 15 à priori (intra‐, peri‐ and post‐OP) risk factors + demographics (e.g. age)
Leung et al., 2013^51^	3	Surgical (noncardiac surgery)	581/234 (40.3%)	73.6 (6.1)	Included	Delirium assessment (structured interview) by trained research assistant one week prior to surgery and on POD 1 and 2 (in total: 3x) using CAM	Medication data extracted from medical record by research assistants	Pre‐OP opioid intake & post‐OP opioid administration for pain treatment (on POD 1 and 2): morphine, fentanyl & hydromorphone as hydromorphone equivalents)	Yes	MRM with risk stratification adjusted for: risk factors with p‐values ≤0.20 in bivariate analysis (including i.a. age, sex, cognitive impairment, history of CNS disorder, surgical type & risk)
Limpawattana et al., 2016^52^	4	(Medical) ICU	99/44 (44.4%)	78.8 (7.3)^c^	Included	Daily delirium assessment by trained clinical researchers until nondelirious state, discharge or death using CAM‐ICU	Medication data source not specified, extracted by clinical researchers (ICU nurses)	Baseline & ICU medication: corticosteroids, benzodiazepines, antihistamines, antibiotics	Yes	MRM adjusted for five risk factors: previous stroke, physical restraints, bed change >3, use of bladder catheter, on mechanical ventilators
Pasinska et al., 2018^53^	3	Stroke unit	750/203 (27.1%)	71.8 (13.1)	Included	Daily assessment by a neurologist for 7 days using bCAM, CAM‐ICU & DSM‐V criteria	Medication data source not specified; no details on data extraction	Use of in‐hospital medication: anticoagulants, heparin, insulin, beta blockers, diuretics	Not specified	MRM adjusted for: variables significantly associated with delirium in the univariable logistic regression
Sieber et al., 2011^39^	3	Surgical (hip surgery)	236/60 (25.4%)	83.0 (6.9)^c^	Included	Delirium assessment pre‐OP & on POD 2 by either a geriatrician or a trained research nurse using CAM	Medication data extracted from patient database by research nurses	Intra‐OP (midazolam) and post‐OP medication (opioids)	Yes	MRM adjusted for: pain, opioid use, dementia, ICU admission and other demographic variables
Slor et al., 2011^40^	3	Surgical (hip surgery)	526/60 (11.4%)	76.7 (5.5)^e^	Included	Daily delirium assessment by research nurses (CAM, DRS‐R‐98), validated by geriatrician (DSM‐IV criteria) until POD 5	Medication data extracted from medical records by blinded research nurses	Peri‐OP medication: benzodiazepines (diazepam, lorazepam, midazolam, oxazepam, temazepam); opioids (alfentanil, morphine, nalbuphine, piritramide, sufentanil); anticholinergics (atropine & ipratropium)	Yes	MRM adjusted for: demographics and predefined risk factors with p‐value <0.10 in univariable analysis (including i.a. age, cognitive impairment, illness severity, visual impairment)
Schor et al.^h^, 1992^41^	3	General medical & surgical	291/91 (31.3%)	80.5 (7.6)	Included	Delirium assessment within 48 h of admission & daily for 14 days by authors using DSI & DSM‐III criteria; consecutive evaluation on periodic basis until discharge or death; additionally: primary nurse interview & medical chart review for evidence of symptoms of delirium 24 h prior to each day's assessment	Medication data from nursing medication records, extracted by authors	Baseline medication: antipsychotics, opioids, benzodiazepines, anticholinergics, steroids, H_2_‐blockers, NSAIDs, Digoxin	Yes	MRM adjusted for: all variables with age‐ and sex‐adjusted p‐values <0.10 in univariable analysis (including i.a. cognitive impairment, infection, fracture on admission, opioid and antipsychotic use)
Pandharipande et al.^h^, 2006^42^	3	ICU (medical & coronary)	198/unclear (n of delirium cases not stated)	55.5 (17.0)	Included	Daily delirium assessment by independent ICU nurses using CAM‐ICU & DSM‐IV criteria; time frame not stated	Medication data from ICU records; extraction process not stated	ICU medication: sedatives (lorazepam, midazolam), analgesics (fentanyl, morphine), anesthetics (propofol)	Yes (24 h)	MRM adjusted for: age, sex, visual & hearing deficits, history of dementia, depression, severity of illness (modified APACHE II‐score), sepsis, history of neurologic disease, hematocrit, and daily serum glucose concentrations
Van der Mast et al.^h^, 1999^57^	3	Surgical (cardiac surgery)	296 /40 (13.5%)	63 (11)	Included	Post‐OP delirium assessment on POD 2 to 5 by mental status examination and chart review (according to DSM III‐R criteria); not stated who assessed patients	Medication data from pre‐OP assessment; extraction process not stated	Baseline medication: calcium channel blockers (nifedipine)	Not specified	MRM adjusted for: variables with p‐values <0.10 in univariable analysis (including i.a. age, use of nifedipine, cognitive impairment, amino acid disturbances)
Gustafson et al.^h^, 1988^44^	3	Surgical (hip fracture surgery)	111/68 (61.3%)	79.3 (total range: 65–96)	Included	Delirium assessment by two independent authors pre‐OP until 14 days post‐OP using modified OBS & DSM III criteria; frequency of testing not specified	Medication data from medical records & patient, relative or caregiver interview; extraction process not specified	Baseline medication: Antidepressants, antipsychotics, benzodiazepines, antiparkinsonian drugs & drugs with anticholinergic effect	Not specified	MRM adjusted for: all variables with p‐value <0.05 in univariable analysis (age, dementia, depression, cerebrovascular disease, cardiovascular disease, drugs with anticholinergic effect)
Morrison et al.^h^, 2003^45^	3	Surgical (hip fracture surgery)	541/87 (16.1%)	Not specified (91% > 80a)	Included	Daily delirium assessment by research nurses & assistants within 48 h of admission until discharge using CAM	Medication data from medical records; extraction process not specified	Pre‐ and post‐OP pain medication: Opioids (in morphine sulfate equivalents, meperidine)	Yes	MRM adjusted for: variables with p < 0.15 in univariable analysis (including i.a. age, sex, cognitive impairment, residency in a nursing home, medical complication, heart failure on admission)
(Nested) case–control studies (CCs), *n* = 3										
Perez‐Ros et al.^k^, 2019,^54^	4	Nursing homes	443/83 (18.7%)	85.73 (6.72)	included	Delirium screening by five external nurses (CAM), confirmation by geriatrician or psychiatrist (DSM‐IV‐criteria); time frame & frequency of delirium assessment unclear	Medication data from comprehensive geriatric assessment, extracted by external nurses	Baseline medication: polypharmacy (>7), anxiolytics, anticholinergics, antidepressants, antipsychotics, H_1_‐antihistamines, anti‐inflammatory drugs, corticosteroids	Not specified	MRM adjusted for: predisposing & triggering factors with *p*‐value <0.25 in univariable analysis
Cole et al., 2016^55^	4	Long term care facilities	254/95 (37.4%) Matched controls: 1969	86.3 (6.5)^c^	included	Weekly assessment by research assistants using CAM; 24 weeks follow up time	Medication data from facility pharmacy database; extracted by blinded research assistants	Baseline medication: H_1_‐antihistamines, anti‐inflammatory drugs, corticosteroids	Yes (28 days)	Matched analysis: cases were matched with up to 35 controls; MRM adjusted for dementia status and use of antipsychotics
Marcantonio et al.^h^, 1994^56^	4	Mixed surgical (general, orthopedic & gynecology)	245/91 (37.1%) (sample size of PC: 1341)	73 (8)	included	Daily assessment by study personnel using CAM on POD 2–5 (or until day before discharge); additionally: daily review of the medical record and data from hospital's nursing intensity index	Data from medical records (nurses' medication flow sheet), extracted by blinded reviewer	Post‐OP medication: opioids, benzodiazepines (*long‐acting agents*: chlordiazepoxide, dia‐, flurazepam; *short‐acting agents*: oxa‐, lora‐, temazepam, tria‐, midazolam,), H_1_‐antihistamines (diphenhydramine)	Yes (24 h)	Matched analysis: cases were matched with one or two controls; MRM adjusted for exposure to all other drugs within that class

Abbreviations: ADE, adverse drug event; ALI, acute lung injury; APACHE, Acute Physiology And Chronic Health Evaluation; CAM, Confusion Assessment Method; CC, nested case–control study; CCI, Charlson Comorbidity Index; CEBM, Oxford Centre for Evidence‐based Medicine; DOSS, Delirium Observation Screening Scale; DRS, Delirium Rating Scale (98‐revised); DSI, Delirium Symptom Interview; DSM (‐III/‐IV/‐V‐, TR), Diagnostic and Statistical Manual of Mental Disorders (3rd, 4th, 5th Edition, Text Revision); i.a., inter alia; ICDSC, Intensive Care Delirium Screening Checklist; ICU, Intensive Care Unit; ID, incident delirium (postoperatively); IQCODE, Informant Questionnaire on Cognitive Decline in the Elderly; ITT, intention to treat analysis; MRM, multivariable regression model; N, number; N.A., not applicable; NOS, Newcastle Ottawa Scale; OBS, Organic Brain Syndrome Scale; OP, operation/operative; PC, prospective cohort study; PD, prevalent delirium (preoperatively); PHC, Penehyclidine hydrochloride; POD, post‐operative day; PP, per protocol analysis; RASS, Richmond Agitation Sedation Scale; RCT, randomized controlled trials; SD, Standard Deviation; SOFA, sequential organ failure assessment.

^a^ Surgical and medical patients.

^b^ Acute admission via emergency department.

^c^ Age of delirious patients.

^d^ Age of patients in the intervention group.

^e^ Age of patients receiving general anesthesia.

^f^ The 85% of delirious patients received entire midazolam dosage before delirium.

^g^ Regarded as dementia was included due to the elevated (mean) age of patients.

^h^ Included in review by Clegg and Young.^9^

^i^ Profound/severe dementia excluded (defined as inability to give informed consent or to communicate).

^j^ Median and interquartile range (IQR).

^k^ RoB assessed as PC.

The RCTs included were conducted as pharmacological prophylaxis studies investigating either haloperidol,^28^, ^29^, ^30^, ^31^, ^33^ olanzapine,^27^ ketamine^32^, ^36^ or corticosteroids.^34^, ^35^ One RCT^26^ investigated the effect of anticholinergics on postoperative cognitive function and delirium rates. The included OS^37^, ^38^, ^39^, ^40^, ^41^, ^42^, ^43^, ^44^, ^45^, ^46^, ^47^, ^48^, ^49^, ^50^, ^51^, ^52^, ^53^, ^54^, ^55^, ^56^ investigated multiple medication classes and agents as possible triggers in prospective designs, including both baseline medication as well as newly prescribed drugs. RCTs examined medications as an intervention, and therefore only evaluated newly started drugs. The included studies were reviewed for whether drug exposure preceded delirium (see Table 1).

Clinical settings and patient populations were heterogeneous across studies. Settings ranged from intensive care units (ICU),^30^, ^31^, ^33^, ^36^, ^37^, ^42^, ^46^, ^47^, ^50^, ^52^ surgical wards (orthopedic hip/knee,^27^, ^28^, ^34^, ^39^, ^40^, ^44^, ^45^ cardiac,^35^, ^43^, ^50^ spine,^48^ thoracoscopic^26^ and mixed),^32^, ^49^, ^51^, ^56^ mixed medical/surgical wards,^29^, ^41^ acute geriatric care unit,^38^ stroke unit^53^ to nursing homes and long term care facilities.^54^, ^55^ There was a broad range of sample sizes in the included studies (from 68^33^ to 1789^30^ participants), with a calculated mean of 401. Delirium incidences were equally heterogeneous, and ranged from 11.4%^40^ to 61.3%^44^ (mean: 29.4%). Mean ages ranged from 55.5^42^ to 86.3^55^ years (mean: 72.9 years). Dementia or cognitive impairment status was excluded in most RCTs^26^, ^27^, ^29^, ^30^, ^33^ and three RCTs excluded severe dementia.^28^, ^31^, ^34^ OS included patients with dementia with the exception of three PCs^46^, ^48^, ^50^ and one PC^47^ did not specify.

The RCTs included were based upon intention‐to‐treat (ITT) analyses with the following exception: two RCTs conducted per‐protocol (PP) analyses,^27^, ^36^ one^30^ analyzed data with both ITT and PP analyses, and one^26^ did not provide information on analysis type. Nonrandomized OS performed multivariable logarithmic or linear regression models that were adjusted for various covariates. The statistical approaches and the covariates considered are depicted in Table 1.

### 3.3 Risk of bias of individual studies

**FIGURE 2:**
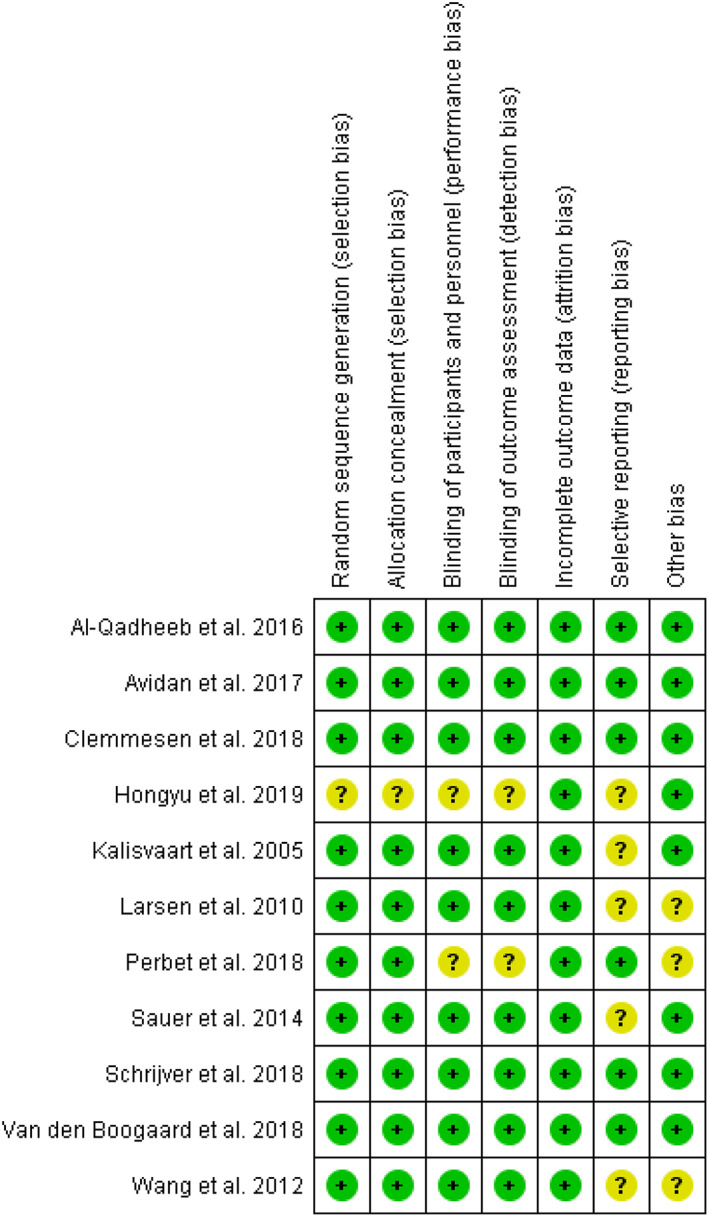
Risk of bias (RoB) assessment of randomized controlled trials (RCTs)

The RoB among the RCTs included was estimated to be low, and is presented in Figure 2 (and Appendix S4). In PCs (Table 2), RoB was balanced overall. Nested CCs^55^, ^56^ did not exhibit limitations or RoB (Table 2). In general, our assessment corresponded to the RoB appraisal by Clegg and Young but two PCs^43^, ^45^ were estimated as of “low quality” by Clegg and Young, while our assessment resulted in a NOS‐score = 7 (comparable to unclear RoB).

**TABLE 2:** Quality and risk of bias assessment using the Newcastle‐Ottawa‐Scale

Cohort studies	Selection				Comparability		Outcome			Assessment		
Study ID (author, year)	Representativeness of the exposed cohort	Selection of the nonexposed cohort	Ascertainment of exposure	Demonstration that outcome of interest was not present at start of study	Comparability of cohorts–main factor: age	Comparability of cohorts–secondary factor: dementia, cognitive impairment/illness severity, multimorbidity	Ascertainment of outcome	Adequate time of follow‐up	Acceptable rate of lost to follow‐up	NOS –Total score	Appraisal	Quality appraisal by Clegg and young
Pasinka et al.; 2018^53^	Yes	Yes	Yes	No	Yes	Yes	Yes	Yes	Yes	8	*Low RoB*	
Perez‐Ros et al. 2019^54^	Yes	Yes	Yes	No	Yes	No	Yes	Yes	Yes	7	*Unclear RoB*	
Brown et al.; 2016^48^	Yes	Yes	Yes	Yes	Yes	No	Yes	Yes	Yes	8	*Low RoB*	
Saljuqi et al.; 2020^49^	Yes	Yes	Yes	No	Yes	No	Yes	Yes	Yes	7	*Unclear RoB*	
Taipale et al.; 2012^50^	Yes	Yes	Yes	No	Yes	Yes	Yes	Yes	Yes	8	*Low RoB*	
Leung et al.; 2013^51^	Yes	Yes	Yes	Yes	Yes	Yes	Yes	No	Yes	8	*Low RoB*	
Limpawattana et al.; 2016^52^	Yes	Yes	Yes	No	No	No	Yes	Yes	Yes	6	*Unclear RoB, probable confounding*	
Sieber et al.; 2011^39^	Yes	Yes	Yes	Yes	Yes	Yes	Yes	No	Yes	8	*Low RoB*	
Slor et al.; 2011^40^	Yes	Yes	Yes	Yes	Yes	Yes	Yes	Yes	Yes	9	*Low RoB*	
Schreiber et al., 2014^46^	Yes	Yes	Yes	Yes	Yes	Yes	Yes	No	Yes	8	*Low RoB*	
Hein et al; 2014^38^	Yes	Yes	Yes	No	Yes	Yes	Yes	No	Yes	7	*Unclear RoB*	
Burry et al.; 2017^37^	Yes	Yes	Yes	Yes	Yes	Yes	Yes	No	Yes	8	*Low RoB*	
Wolters et al.; 2015^47^	Yes	Yes	Yes	Yes	Yes	Yes	Yes	No	Yes	8	*Low RoB*	
Schor et al.^a^; 1992^41^	Yes	Yes	Yes	Yes	Yes	Yes	Yes	Yes	Yes	9	*Low RoB*	*Moderate study quality*
Pandharipande et al.^a^; 2006^42^	Yes	Yes	Yes	Yes	Yes	Yes	Yes	No	Yes	8	*Low RoB*	*Moderate study quality*
Van der Mast et al.^a^; 1999^57^	Yes	Yes	Yes	No	Yes	Yes	No	Yes	Yes	7	*Unclear RoB*	*Low study quality*
Gustafson et al.^a^; 1988^44^	Yes	Yes	Yes	No	Yes	Yes	No	Yes	Yes	7	*Unclear RoB*	*Low study quality*
Morrison et al.^a^; 2003^45^	Yes	Yes	Yes	Yes	Yes	Yes	Yes	Yes	Yes	9	*Low RoB*	*Moderate study quality*
Case–control studies	Selection				Comparability		Exposure			Assessment		
Study ID (author, year)	Adequate case definition	Representativeness of the cases	Selection of controls	Definition of controls	Comparability of cases and controls–main factor: age	Comparability of cases and controls–secondary factor: Dementia, cognitive impairment/illness severity, multimorbidity	Ascertainment of exposure	Same method of ascertainment for cases and controls	Nonresponse rate	NOS –total score	Appraisal	*Quality appraisal by Clegg and Young*
Cole et al. 2016^55^	Yes	Yes	Yes	Yes	Yes	Yes	Yes	Yes	Yes	9	*Low RoB*	
Marcantonio et al.^a^; 1994^56^	Yes	Yes	Yes	Yes	Yes	Yes	Yes	Yes	Yes	9	*Low RoB*	*moderate study quality*

Abbreviations: NOS, Newcastle Ottawa Scale; RoB, risk of bias.

^a^ Studies included in the review by Clegg and Young.^9^

### 3.4 Results of individual studies

Single medication associations (uni‐ and multivariable effect estimates and raw data) are presented in a comprehensive summary table in the Appendix S1.

### 3.5 Results of syntheses

We established a hierarchy of evidence, including meta‐analyses and single effect estimates on medication associations with delirium. Comprehensive meta‐analyses and sensitivity analyses are presented in the Appendix S2. The assessment of confidence in evidence is depicted as GRADE profiles in Table 3.

**TABLE 3:** Quality of evidence table according to medication classes (GRADE profiles)

			Certainty assessment					No of participants		Effect measure		Confidence in evidence
Medication class	No of studies (study ID)	Study design	Risk of bias	Inconsistency	Indirectness	Imprecision	Other considerations	Exposure delirious/total (delirium incidence)	Nonexposure delirious/total (delirium incidence)	Relative (95% CI)	Absolute (95% CI)	
Antipsychotics												
Haloperidol	5	RCTs	Not serious^d^	Not serious	Not serious	Not serious	None	346/1325 (26.1%)	348/1311 (26.5%)	Pooled OR 0.96 (0.72 to 1.28)	8 fewer per 1.000 (from 59 fewer to 51 more)	⨁⨁⨁⨁ High
Olanzapine	1 (Larsen 2010)^27^	RCT	Not serious^e^	Not serious	Not serious	Serious^f^	None	28/196 (14.3%)	82/204 (40.2%)	OR 0.25 (0.15 to 0.40)	258 fewer per 1.000 (from 310 fewer to 190 fewer)	⨁⨁⨁◯ Moderate
Corticosteroids	2	RCTs	Not serious	Not serious^l^	Not serious	Serious^f^	None	62/426 (14.6%)	74/428 (17.3%)	Pooled OR 0.69 (0.32 to 1.50)	47 fewer per 1.000 (from 110 fewer to 66 more)	⨁⨁⨁◯ Moderate
Ketamine	2	RCTs	Not serious^o^	Not serious^p^	Not serious	Serious^f^	None	102/517 (19.7%)	73/299 (24.4%)	Pooled OR 0.72 (0.35 to 1.46)	55 fewer per 1.000 (from 143 fewer to 76 more)	⨁⨁⨁◯ Moderate
Anticholinergics	1 (Hongyu 2019)^26^	RCT	Not serious^a^	Not serious	Not serious	Very serious^b^	None	23/60 (38.3%)	5/30 (16.7%)	OR 3.11 (1.04 to 9.26)	217 more per 1.000 (from 6 more to 483 more)	⨁⨁◯◯ Low
Atropine	1 (Hongyu 2019)^26^	RCT	Not serious^a^	Not serious	Not serious	Very serious^c^	None	7/30 (23.3%)	5/30 (16.7%)	OR 1.52 (0.42 to 5.47)	66 more per 1.000 (from 89 fewer to 356 more)	⨁⨁◯◯ Low
PHC	1 (Hongyu 2019)^26^	RCT	Not serious^a^	Not serious	Not serious	Very serious^b^	None	16/30 (53.3%)	5/30 (16.7%)	OR 5.71 (1.72 to 18.94)	366 more per 1.000 (from 89 more to 624 more)	⨁⨁◯◯ Low
ACE inhibitors	1 (Brown 2016)^48^	PC	Serious^w^	Not serious	Not serious	Serious^q^	None	11/22 (50.0%)	25/67 (37.3%)	OR 1.68 (0.64 to 4.44)	127 more per 1.000 (from 97 fewer to 352 more)	⨁◯◯◯ Very low
Antibiotics	1 (Limpawattana 2016)^52^	PC	Serious^x^	Not serious	Not serious	Serious^q^	None	8/18 (44.4%)	36/81 (44.4%)	OR 1.00 (0.36 to 2.79)	0 fewer per 1.000 (from 221 fewer to 246 more)	⨁◯◯◯ very low
Anticoagulants	1 (Pasinska 2018)^53^	PC	Serious^w^	Not serious	Not serious	Serious^f^	None	24/172 (14.0%)	34/488 (7.0%)	OR 2.17 (1.24 to 3.77)	70 more per 1.000 (from 15 more to 151 more)	⨁◯◯◯ very low
Antidepressants	1 (Brown 2016)^48^	PC	Not serious	Not serious	Not serious	Serious^b^	None	16/26 (61.6%)	20/63 (31.7%)	aOR 4.71 (1.03 to 21.50)	369 more per 1.000 (from 6 more to 592 more)	⨁◯◯◯ very low
Antiparkinsonian drugs	1 (Gustafson 1988)	PC	Serious^w^	Not serious	Not serious	Serious^t^	None	12/16 (75.0%)	56/95 (58.9%)	OR 2.09 (0.63 to 6.96)	161 more per 1.000 (from 115 fewer to 320 more)	⨁◯◯◯ very low
Benzodiazepines	4	PCs	Not serious	Serious^g^	Not serious	Serious^h^	Dose response gradient^i^	n.e.^z^	n.e.^z^	Pooled aOR 0.94 (0.63 to 1.41)	n.e.^z^	⨁◯◯◯ very low
	1 (non‐pooled; Burry 2017)^37^									aHR 1.89 (1.16 to 3.08)		
Midazolam	2	PCs	Serious^j^	Serious^k^	Not serious	Serious^h^	Dose response gradient^i^	n.e.^z^	n.e.^z^	Pooled aOR 0.86 (0.21 to 3.47)	n.e.^z^	⨁◯◯◯ very low
Lorazepam	1 (Pandharipande 2006)^42^	PC	Not serious	Not serious	Not serious	Serious^h^	Dose response gradient^i^	n.e.^z^	n.e.^z^	aOR 1.24 (1.08 to 1.43)	n.e.^z^	⨁◯◯◯ very low
Beta‐blockers	1 (Burry 2017)^37^	PC	Not serious	Not serious	Not serious	Serious^c^	None	136/220 (61.8%)	124/300 (41.3%)	HR 1.26 (0.66 to 2.42)	n.e.	⨁◯◯◯ very low
Calcium channel blockers (Nifedipine)	1 (Van der Mast 1999)^43^	PC	Not serious	Not serious	Not serious	Serious^f^	None	11/53 (20.8%)	29/243 (11.9%)	aOR 2.43 (1.01 to 5.82)	128 more per 1.000 (from 1 more to 322 more)	⨁◯◯◯ very low
Digoxin	1 (Schor 1992)^41^	PC	Not serious	Not serious	Not serious	Serious^f^	None	19/73 (26.0%)	72/218 (33.0%)	aOR 0.52 (0.30 to 0.90)	126 fewer per 1.000 (from 190 fewer to 23 fewer)	⨁◯◯◯ very low
Diuretics	1 (Pasinska 2018)^53^	PC	Serious^w^	Not serious	Not serious	Serious^f^	None	54/168 (32.1%)	103/488 (21.1%)	OR 1.77 (1.20 to 2.62)	110 more per 1.000 (from 32 more to 201 more)	⨁◯◯◯ very low
H_1_‐antihistamines	2	CCs	Not serious	Serious^m^	Not serious	Serious^n^	None	13/198 (6.6%)	173/2111 (8.2%)	Pooled aOR 0.75 (0.14 to 4.16)	19 fewer per 1.000 (from 70 fewer to 189 more)	⨁◯◯◯ very low
Diphenhydramine	2	CCs	Not serious	Not serious	Not serious	Serious^n^	None	10/60 (16.7%)	176/2249 (7.8%)	Pooled aOR 1.50 (0.64 to 3.54)	35 more per 1.000 (from 27 fewer to 153 more)	⨁◯◯◯ very low
H_2_‐antihistamines	1 (Schor 1992)^41^	PC	Not serious	Not serious	Not serious	Serious^q^	None	41/121 (33.9%)	50/170 (29.4%)	aOR 1.42 (0.81 to 2.49)	78 more per 1.000 (from 42 fewer to 215 more)	⨁◯◯◯ very low
Heparin	1 (Pasinska 2018)^53^	PC	Serious^w^	Not serious	Not serious	Serious^f^	None	13/168 (7.7%)	15/488 (3.1%)	OR 2.64 (1.23 to 5.68)	47 more per 1.000 (from 7 more to 122 more)	⨁◯◯◯ very low
Insulin	1 (Pasinska 2018)^53^	PC	Serious^w^	Not serious	Not serious	Serious^f^	None	24/171 (14.0%)	33/488 (6.8%)	OR 2.25 (1.29 to 3.93)	73 more per 1.000 (from 18 more to 154 more)	⨁◯◯◯ very low
NSAIDs	2	1 PC and 1 CC	Not serious	Not serious	Not serious	Serious^n^	None	40/845 (4.7%)	146/1467 (9.9%)	Pooled aOR 0.80 (0.33 to 1.96)	18 fewer per 1.000 (from 64 fewer to 79 more)	⨁◯◯◯ very low
ASA	1 (Cole 2016)^55^	CC	Not serious	Not serious	Not serious	Serious^q^	None	37/826 (4.5%)	58/1195 (4.8%)	aOR 1.07 (0.70 to 1.65)	3 more per 1.000 (from 14 fewer to 29 more)	⨁◯◯◯ very low
Naproxen	1 (Cole 2016)^55^	CC	Not serious	Not serious	Not serious	Serious^q^	None	1/22 (4.5%)	94/2042 (4.6%)	aOR 0.73 (0.10 to 5.33)	13 fewer per 1.000 (from 41 fewer to 159 more)	⨁◯◯◯ very low
Opioids	3	PCs	Not serious	Not serious^r^	Not serious	Serious^h^	Dose response gradient^i^	n.e.^z^	n.e.^z^	Pooled aOR 1.50 (0.91 to 2.49)	n.e.^z^	⨁◯◯◯ very low
Morphine	2	1 PC & 1 CC	Not serious	Not serious	Not serious	Serious^h^	None	n.e.^z^	n.e.^z^	Pooled aOR 1.09 (0.95 to 1.25)	n.e.^z^	⨁◯◯◯ very low
Fentanyl	2	1 PC & 1 CC	Not serious	Not serious	Not serious	Serious^s^	None	n.e.^z^	n.e. ^#^	Pooled aOR 1.22 (0.99 to 1.51)	n.e.^z^	⨁◯◯◯ very low
Pethidine (= Meperidine)	1 (Morrison 2003)^45^	PC	Not serious	Not serious	Not serious	Serious^f^	None	27/129 (20.9%)	56/412 (13.6%)	aRR 2.44 (1.32 to 4.50)	196 more per 1.000 (from 44 more to 476 more)	⨁◯◯◯ very low
Oxycodone	1 (Marcantonio 1994)^56^	CC	Not serious	Not serious	Not serious	Serious^t^	None	9/39 (23.1%)	82/206 (39.8%)	aOR 0.69 (0.30 to 1.60)	85 fewer per 1.000 (from 233 fewer to 116 more)	⨁◯◯◯ very low
Codeine	1 (Marcantonio 1994)^56^	CC	not serious	Not serious	Not serious	Serious^t^	None	6/17 (35.3%)	85/228 (37.3%)	aOR 1.15 (0.37 to 3.56)	33 more per 1.000 (from 193 fewer to 306 more)	⨁◯◯◯ very low
Paracetamol	1 (Cole 2016)^55^	CC	Not serious	Not serious	Not serious	Serious^y^	None	42/989 (4.2%)	53/1075 (4.9%)	aOR 0.81 (0.52 to 1.26)	9 fewer per 1.000 (from 23 fewer to 12 more)	⨁◯◯◯ very low
Polypharmacy	2	PCs	Not serious^u^	Not serious^v^	Not serious	Serious^t^	None	82/270 (30.4%)	58/285 (20.4%)	Pooled aOR 1.60 (0.93 to 2.76)	87 more per 1.000 (from 12 fewer to 210 more)	⨁◯◯◯ very low
Propofol	1 (Pandharipande 2006)^42^	PC	Not serious	Not serious	Not serious	Serious^h^	None	n.e.^z^	n.e.^z^	aOR 1.24 (0.90 to 1.70)	n.e.^z^	⨁◯◯◯ very low

Abbreviations: a, adjusted; CC, nested case–control study; CI, confidence interval; HR, hazard ratio; n.e., not estimable; OR, odds ratio; PC, prospective cohort study; RCT, randomized controlled trial; RR, risk ratio.

^a^ Cochrane RoB of the RCT: 5/7 domains show unclear risk of bias.

^b^ Low sample size, insufficient OIS, and broad confidence interval.

^c^ Lower 95% CI < 1.00 (statistically insignificant estimate), low sample size, and insufficient OIS.

^d^ 2/5 RCTs show unclear risk of reporting bias.

^e^ Unclear risk of reporting bias and possible confounding with alcohol withdrawal symptoms.

^f^ Insufficient OIS.

^g^ I^2^ = 76%, *p* = 0.006.

^h^ Lower 95% CI < 1.00; OIS not estimable due to lack of data.

^i^ Dose–response‐gradient does not lead to an upgrade in the level of evidence due to previous downgrading.

^j^ 1/2 PCs not adjusted for possible confounders.

^k^ I^2^ = 85%, *p* = 0.009.

^l^ Borderline heterogeneity: I^2^ = 63%, *p* = 0.10.

^m^ I^2^ = 83%, *p* = 0.01.

^n^ Lower 95% CI <1.00, borderline OIS.

^o^ Unclear risk of detection and performance bias due to lack of information on blinding and possible confounding of prevalent and incident delirium in one RCT (Perbet 2018).

^p^ Borderline heterogeneity of (I^2^ = 69%, *p* = 0.07) is accepted by review authors due to the direction of point estimates.

^q^ Insufficient OIS, lower 95% CI <1.00.

^r^ Heterogeneity of I^2^ = 47%, *p* = 0.15.

^s^ Lower 95% CI 0.99 (borderline), in combination with inestimable but probably insufficient OIS review authors had to downgrade one level.

^t^ Insufficient OIS, lower 95% CI <1.00.

^u^ Confounding with prevalent delirium possible.

^v^ I^2^ = 63%, *p* = 0.08; interpreted as a borderline estimate for heterogeneity.

^w^ No adjustment for possible confounding (unadjusted estimates).

^x^ Serious risk of confounding (bias).

^y^ insignificant effect measure.

^z^ Pandharipande et al. 2006 do not present raw data on delirium and medication exposure.

#### 3.5.1 Medication classes: High to low evidence quality levels

##### Antipsychotics

There is high quality evidence from five pooled RCTs^28^, ^29^, ^30^, ^31^, ^33^ that haloperidol does not significantly increase the risk of delirium. Meta‐analysis included 2636 patients and the pooled effect estimate ranges around the null effect (odds ratio (OR) 0.96, 95% confidence interval (CI): 0.72–1.28; absolute risk (AR) reduction (ARR) 8 per 1000, 95% CI: 59 fewer to 51 more). Studies investigated dosages from 1.5 mg/d p.o.^28^ to 6 mg/d i.v. The study participants were investigated in heterogeneous clinical settings (ICU,^30^, ^31^, ^33^ mixed medical/surgical,^29^ hip surgery),^28^ and patients with dementia^29^, ^30^, ^33^ (respectively severe dementia)^28^, ^31^ were excluded. RoB assessment did not demonstrate significant risk, although two RCTs^28^, ^31^ show an unclear risk of reporting bias. Heterogeneity of effect estimates was acceptable with an I^2^ of 44% (*p* = 0.13).

The moderate quality evidence of one RCT^27^ suggests a reduction of delirium incidence with prophylactic olanzapine (OR 0.25, 95% CI: 0.15–0.40). ARR is 258 per 1000 fewer delirium cases when exposed to 10 mg olanzapine (95% CI: 310 fewer to 190 fewer). RoB was estimated as low, although reporting bias could not be excluded and confounding with alcohol withdrawal symptoms might be possible. GRADE‐rating needed to be downgraded by one level (to *moderate*) due to insufficient optimal information size (OIS).^25^

##### Corticosteroids

Corticosteroids show no significantly increased risk for delirium in our meta‐analysis of two RCTs^34^, ^35^ (pooled OR 0.69, 95% CI: 0.32–1.50). AR is 47 fewer per 1000 (95% CI: 110 fewer to 66 more). Pooled studies^34^, ^35^ included 854 surgical patients and did not exhibit increased RoB. Patients with severe dementia were excluded in one study^34^ and in another^35^ dementia status was not specified. The GRADE‐rating was *moderate* because of insufficient OIS. Heterogeneity calculations showed a borderline result (I^2^ = 63%, *p* = 0.10) but sensitivity analyses did not change results significantly (comparison to meta‐analysis of OS as well as RCTs and OS). Borderline heterogeneity might be explained by different agents and dosages (125 mg i.v. methylprednisolone^34^ vs 1 mg/kg i.v. dexamethasone).^35^ The (mean) equivalent doses^58^ studied differed by approximately three and a half times (higher equivalent dose of dexamethasone) but neither study indicated increased delirium risk.

##### Ketamine

There is moderate quality of evidence from two RCTs^32^, ^36^ that ketamine does not increase delirium rates in subanesthetic dosages (0.2 mg/kg/h^36^ respectively 0.5 or 1.0 mg/kg i.v. as a bolus^32^; pooled OR 0.72, 95% CI: 0.35–1.46). ARR is 55 fewer per 1000 (95% CI: 143 fewer to 76 more). Pooled RCTs investigated 816 (surgical^32^ and ICU)^36^ patients. Dementia status was not specified. RoB across studies was estimated as low, although detection and performance bias could not be excluded in one RCT.^36^ Borderline heterogeneity (I^2^ = 69, *p* = 0.07) was accepted because point estimates did not differ in direction. The GRADE‐rating had to be downgraded by one level to *moderate* due to serious imprecision.

##### Anticholinergics

There is low quality evidence from one RCT^26^ that anticholinergics increase delirium rates. Atropine and penehyclidine hydrochloride (PHC) were investigated in 90 (thoracoscopic) surgical patients^26^ (with negative dementia status). When pooling both agents, anticholinergics as a medication class demonstrate an OR of 3.11 (95% CI: 1.04–9.26). AR is 217 more per 1000 (95% CI: 6 more to 483 more). The evidence quality rating needed to be downgraded by two levels (to *low*) due to very serious imprecision. Atropine (0.01 mg/kg i.m.) shows an insignificant result (OR 1.52, 95% CI: 0.42–5.47; AR: 66 more per 1000, 95% CI: 89 fewer to 356 more). PHC shows a strong association with delirium, but confidence intervals are very broad (OR 5.71, 95% CI: 1.72–18.94; AR: 366 more per 1000, 95% CI: 89 more to 624 more).

#### 3.5.2 Medication classes: very low‐evidence quality levels

All other medication classes investigated in OS reached only *very low* quality of evidence: ACE‐inhibitors, antibiotics, oral anticoagulants, antidepressants, antiparkinsonian drugs, benzodiazepines, beta‐blockers, calcium‐channel blockers, digoxin, diuretics, H_1_‐ and H_2_‐antihistamines, heparin, Insulin, NSAIDs, paracetamol, opioids, propofol and polypharmacy (as a delirium‐associated drug entity). The results for selected medication that is commonly regarded as being delirium‐associated^5^, ^57^ are described.

##### Antidepressants

Antidepressants as a general drug class were investigated in one study (89 patients with negative dementia status undergoing spine surgery).^48^ Multivariable regression analysis shows a positive association with delirium (adjusted OR 4.71, 95% CI: 1.03–21.20). AR is 369 more per 1000 (95% CI: 6 more to 592 more). The GRADE‐rating had to be downgraded to *very low* due to serious imprecision.

##### Benzodiazepines

Benzodiazepines did not significantly increase or decrease delirium rates in our meta‐analysis of four PCs^40^, ^41^, ^42^, ^46^ which resulted in a pooled effect estimate around the null effect (aOR 0.94, 95% CI: 0.63–1.41). Studies investigated 1345 patients in total in heterogeneous settings (ICU,^42^, ^46^ mixed medical/surgical,^41^ hip surgery),^40^ and included dementia status with one exception.^46^ RoB was estimated as low (NOS‐8^42^, ^46^ respectively NOS‐9).^40^, ^41^ One PC^37^ investigating 520 ICU‐patients (dementia included) showed a significant association for exposure to benzodiazepines 48 h prior to delirium onset (adjusted hazard ratio [aHR] 1.89, 95% CI: 1.16–3.08), but could not be pooled (due to the noncomparable effect estimate). The certainty of evidence needed to be downgraded to *very low* due to serious inconsistency (I^2^ = 76%) and serious imprecision. AR estimates could not be calculated. Sensitivity analysis (comparison to a meta‐analysis of unadjusted ORs of eight studies^37^, ^39^, ^40^, ^41^, ^44^, ^48^, ^52^, ^56^ including in total 2117 participants) did not change the results significantly (see Appendix S4). High heterogeneity may be explained by heterogeneous clinical settings, and even increased in the sensitivity analysis (I^2^ = 90%).

A dose–response gradient is shown in four OS^37^, ^46^, ^50^, ^56^: Systemic administration of >5 mg midazolam‐equivalent in 330 ICU‐patients resulted in a borderline insignificant association (aOR 1.02, 95% CI: 0.99–1.05).^46^ Taipale et al.^50^ compared >3 mg to <3 mg midazolam‐equivalent administration (OR 2.23, 95% CI: 1.06–4.96) and dose increment with every additional mg midazolam‐equivalent (aOR 1.08, 95% CI: 1.00–1.16) in 122 ICU‐patients (with negative dementia status). Burry et al.^37^ presented an aHR of 1.08 (95% CI: 1.04–1.12) for 5 mg midazolam‐equivalent in 520 ICU‐patients. Marcantonio et al.^56^ showed an aOR of 3.32 (95% CI: 1.03–10.70) for a dosage of >5 mg diazepam‐equivalent in 245 surgical patients. Evidence quality remains at a *very low* level because upgrading (dose–response gradient) was not possible due to prior downgrading (serious inconsistency and imprecision).^59^

Among specific agents, lorazepam exhibited an increased risk for delirium and a dose–response gradient in 198 ICU‐patients (aOR of 1.24 [95% CI: 1.08–1.43]).^42^ AR could not be calculated due to a lack of data. A daily dose of >20 mg achieved 100% delirium probability. Confidence in evidence remained *very low* due to serious imprecision.

Meta‐analysis of two PCs^39^, ^42^ investigating 434 patients (dementia status included) showed an insignificant association between midazolam and delirium. One unadjusted univariable estimate^39^ was pooled with an adjusted multivariable estimate^42^ and resulted in an OR of 0.86 (95% CI: 0.21–3.47). AR calculations could not be conducted due to a lack of data.^42^ Heterogeneity was high, with I^2^ of 85% which might be explained by heterogeneous clinical settings (hip surgery patients receiving midazolam intra‐ and postoperatively^39^ versus ICU‐patients being sedated with midazolam)^42^ and pooled unadjusted and adjusted effect estimates. Overall the quality of evidence needed to be downgraded to *very low* due to serious RoB, inconsistency and imprecision.

##### H_1_ ‐antihistamines

Meta‐analysis of two CCs^55^, ^56^ on H_1_‐antihistamines demonstrated an insignificant effect estimate in 2309 patients (dementia status included): aOR of 0.75, 95% CI: 0.14–4.16. AR resulted in 19 fewer cases per 1000 (95% CI: 70 fewer to 189 more). Evidence quality needed to be downgraded to *very low* due to serious inconsistency (I_2_ = 83%) and imprecision. Heterogeneity might be explained by the fact that H_1_‐antihistamines as a medication class^55^ were compared to diphenhydramine as a specific agent.^56^ The clinical settings were also heterogeneous (long term care facilities^55^ vs. mixed surgical).^56^

Heterogeneity was low when results for diphenhydramine were pooled (I^2^ = 0%, *p* = 0.38).^55^, ^56^ The effect estimate indicates an insignificant positive association (aOR 1.50, 95% CI: 0.64–3.54; AR: 35 more per 1000, 95% CI: 27 fewer to 153 more). GRADE‐rating results were the same for diphenhydramine as for H_1_‐antihistamines as a medication class (*very low*) because the same studies were included.

##### Opioids

Meta‐analysis of three PCs^39^, ^41^, ^42^ on opioids revealed a statistically nonsignificant association with delirium (aOR 1.50, 95% CI: 0.91–2.49). Heterogeneity was acceptable, with an I^2^ of 47% (*p* = 0.15). Confidence in evidence needed to be downgraded to *very low* due to serious imprecision. AR was not calculable due to a lack of raw data in one PC.^42^ Studies pooled investigated 725 patients in heterogeneous clinical settings, with dementia status being included. RoB assessment resulted in low probability for biased results. Sensitivity analysis changed results significantly: comparison with a meta‐analysis of eight pooled studies^37^, ^39^, ^40^, ^41^, ^45^, ^48^, ^51^, ^56^ (univariable unadjusted estimates, including 3029 patients in total) resulted in a significant positive association (OR 1.50, 95% CI: 1.15–1.95). This might be explained by confounding due to nonadjusted effect estimates.

Opioids exhibited a dose–response gradient: a postoperative dose of >8 mg hydromorphone‐equivalent in comparison to <8 mg results in an adjusted OR of 2.60 (95% CI: 1.31–5.17)^51^ (low‐delirium risk and low‐pain scores of 145 surgical patients). One PC^58^ on 541 hip fracture patients presented an inverse association: the administration of <10 mg parenteral morphine‐equivalent per day postoperatively resulted in an increased risk (aOR 5.43, 95% CI: 2.40–12.30) compared to higher doses. This was interpreted as pain, and insufficient pain treatment being a precipitating factor for delirium.^45^

The specific agents morphine, fentanyl and meperidine showed higher possible risk than other opioid agents, although evidence levels are *very low* (serious imprecision, insufficient OIS): the meta‐analysis (one PC^42^ with 198 ICU‐patients and one CC^56^ with 245 mixed surgical patients) of morphine showed a nonsignificant delirium association (aOR 1.09, 95% CI: 0.95–1.25). The heterogeneity calculation was low (I^2^ = 0%, *p* = 0.78).

The same two studies^42^, ^56^ presented associations for fentanyl with a borderline insignificant result (aOR 1.22, 95% CI: 0.99–1.51). Heterogeneity was low (I^2^ = 0%). AR estimates could not be calculated. The GRADE‐rating for fentanyl and morphine was downgraded to *very low* due to serious imprecision.

One PC^58^ on postoperative pain and opioid administration showed a significant association of meperidine with delirium (aRR 2.44, 95% CI: 1.32–4.50) in 541 patients undergoing hip surgery. AR results in 196 more cases per 1000 (95% CI: 44 more to 476 more). The results did not change in sensitivity analysis with two OS^45^, ^56^ (OR 2.07, 95% CI: 1.36–2.80).

In comparison, oxycodone and codeine did not show an association with delirium (oxycodone: aOR 0.69, 95% CI: 0.30–1.60; AR: 85 fewer per 1000, 95% CI: 233 fewer to 116 more; codeine: aOR 1.15, 95% CI: 0.37–3.56; AR: 33 more per 1000, 95% CI: 193 fewer to 306 more).^56^ The GRADE‐rating was downgraded to *very low* due to insufficient OIS.

##### Polypharmacy

The pooled adjusted estimates from two PCs^38^, ^49^ on polypharmacy showed an insignificant association with delirium (aOR 1.60, 95% CI: 0.93–2.76). In total, 555 geriatric^38^ and surgical^49^ patients (dementia status included) were included. AR was 87 per 1000 (95% CI: 12 less to 210 more). RoB was estimated as low, although incident delirium could have been confounded with prevalent delirium in both studies.^38^, ^49^ Meta‐analysis showed a borderline heterogeneity (I^2^ = 67%), possibly due to heterogeneous definitions of polypharmacy (≥3 medications^49^ and ≥5 medications^38^). The GRADE‐rating needed to be downgraded to *very low* due to insufficient OIS. Sensitivity analysis exhibited a significant difference: meta‐analysis of unadjusted estimates of the same PCs^38^, ^49^ and one CC^54^ (in total 998 patients) resulted in a significant pooled unadjusted estimate (OR 1.55, 95% CI: 1.07–2.26). The difference might be explained by confounding due to unadjusted effect estimates.

## 4 DISCUSSION

This update‐review with meta‐analyses and GRADE certainty of evidence assessments was conducted to further explore the fragmentary evidence of delirium‐associated medication. In general, the overall evidence (still) remains incomplete, and mostly of low quality, but some findings deserve attention, especially in the context of other evidence‐based works.

The NICE‐guidelines^11^ and two systematic reviews^8^, ^9^ describe positive delirium associations with antipsychotics as a medication group in OS. Sensitivity analysis indicates a contrast between positive associations from OS and insignificant associations in RCTs, which might be explained by possible confounding (as antipsychotics are frequently used in delirium treatment). The high quality of evidence found in our meta‐analysis underlines that there is no significantly increased delirium risk with haloperidol. Moderate evidence shows a reduction of delirium incidences for olanzapine. More research is needed concerning antipsychotics having a possible effect on delirium duration, as described in the NICE‐guidelines^11^ (“low quality evidence”), particularly on side effects. There is no reliable evidence base nor recommendation for the use of antipsychotics as a pharmacological prevention (e.g., olanzapine) or treatment (e.g., haloperidol) as shown in Cochrane reviews,^7^, ^60^ delirium guidelines^11^, ^61^ and central RCTs.^6^, ^62^

Cholinergic deficiency and anticholinergic toxicity are considered the main pathophysiological correlates in delirium genesis.^2^, ^6^ Nevertheless, empirical evidence for anticholinergics is rather weak.^9^ This might, inter alia, be a problem of “too vague” definitions.^11^ We therefore differentiated between primary antimuscarinic anticholinergics (e.g., atropine, scopolamine, oxybutinin, PHC), and drugs with secondary anticholinergic properties (e.g., certain antihistamines, antipsychotics, opioids, tricyclic antidepressants). We did not distinguish between high‐ and low‐potency anticholinergics because there is no consistent correlation between anticholinergic drug load and delirium.^15^, ^63^ In particular, the quantified anticholinergic burden (e.g., measured as serum anticholinergic activity) did not show reproducible and consistent associations with delirium.^64^ We therefore focused on empirical evidence for specific agents. Two reviews^8^, ^9^ included eight OS all providing negative (insignificant) delirium associations with primary anticholinergics. One RCT showed a significantly increased risk for PHC.^26^ When the results for PHC and atropine were pooled, we found a three‐fold increased delirium risk. This must be interpreted with caution, as evidence remains low and a recent meta‐analysis (including 33 Chinese language studies) showed no increased postoperative delirium risk for PHC‐premedication.^65^ Hypothetically, anticholinergic toxicity might instead play a major role in specific patient groups with pre‐existing cholinergic vulnerability, rather than in all delirium cases.^6^ For instance, acetylcholine‐esterase‐receptor antagonists (which increase central cholinergic transmission) failed to show a reduction in delirium rates in patients without preexisting dementia.^60^ Despite inconclusive evidence, we suggest prescribing anticholinergics with precaution.

Only one PC investigated antidepressants as a group and presented an adjusted (insignificant) association with very low‐evidence certainity.^48^ This is consistent with other reviews (each presenting one OS) with very low‐evidence estimations.^8^, ^9^

Benzodiazepines are described as major pharmacological risk factors for inducing delirium due to paradoxical effects.^5^, ^57^ Systematic reviews present both positive and negative results for delirium associations in OS.^8^, ^9^ Clegg and Young^9^ describe “moderate quality evidence” (from seven studies) for increased risk, higher risk in long‐acting agents compared to short‐acting agents as well as dose‐dependent increment of delirium incidences. However, there remains uncertainty due to possible confounding. Clinical guidelines present different evidence according to clinical settings. In non‐ICU‐settings, NICE states with “low quality evidence” that benzodiazepine use is “*not* a significant risk factor.”^11^ The PADIS‐guidelines concerning ICU‐patients presents “strong evidence for an association with delirium.”^61^ Furthermore, “the use of benzodiazepines […] is a risk factor for the duration of delirium in the ICU” (“low quality evidence,” NICE‐guideline).^11^ Our meta‐analysis (of four PCs including 1345 patients) showed an insignificant pooled adjusted OR with very low evidence. High heterogeneity most probably derives from pooling ICU with non‐ICU studies, however, we found evidence from one PC presenting a significant delirium association in 520 ICU‐patients (that could not be pooled in the meta‐analysis), as well as a dose–response gradient in four OS (in ICU‐settings expect of one). We conclude that there is strong evidence for an increased delirium risk with benzodiazepines in the ICU, whereas evidence in non‐ICU‐settings remains inconclusive. Among specific agents, lorazepam exhibited a significant risk for delirium in the ICU and a dose–response gradient when administered for sedation^11^, ^42^ with a very low quality of evidence. Midazolam, on the contrary, did not significantly increase the risk of delirium (“moderate quality evidence”).^11^, ^42^ This is consistent with our meta‐analysis of two PCs, although with very low quality of evidence. Hypothetically, the fact that lorazepam exhibits a longer half‐life (theoretically higher probability of accumulation) and a stronger intrinsic potency compared with midazolam might play a role in delirium genesis. On the other hand, midazolam has active metabolites (while lorazepam does not), possibly leading to pharmacological interactions.^66^ Nevertheless, empirical results for specific agents cannot explain the pathogenesis, and furthermore the quality of evidence (for lorazepam) is very low and insufficient to draw clinical conclusions. Further research is needed, as lorazepam is often the drug of choice in clinical practice.

The anticholinergic properties of first generation H_1_‐antihistamines (e.g., Diphenhydramine) are usually considered deliriogenic.^5^ Clegg and Young described a trend towards increased risk of delirium with H_1_‐antihistamines of “low to moderate quality.”^9^ Our meta‐analysis shows an insignificant effect measure and very low GRADE‐rating (for H_1_‐antihistamines as medication class). The NICE guidelines present “low quality evidence” for an increased risk from diphenhydramine and “very low evidence” not significantly altering delirium risk.^11^ Our meta‐analysis of two CCs for diphenhydramine once more shows an insignificant pooled effect estimate (very low level of evidence). In conclusion, evidence is scarce and of very low quality (for H_1_‐antihistamines in general and diphenhydramine in special), but relatively higher evidence suggests increased delirium risk.

Opioid exposure is considered a well‐established independent risk factor for delirium, although empirical evidence (from OS) is inconsistent.^57^ A review examining opioids (four with positive, eight with insignificant results) suggested significant delirium associations, and showed a 2‐ to 9‐fold increased risk (adjusted ORs: 2.54–9.2).^8^ Our meta‐analysis did not find a significant correlation. The results changed in a sensitivity analysis. This might be explained by confounding (univariable analyses), although hypothetically a significant pooled estimate might have been reached with more statistical power. In conclusion, the GRADE level for opioids as a delirium risk factor is very low, however, opioid exposure shows a dose‐dependent risk in included OS which is consistent with other evidence.^8^, ^11^ There is also evidence from two PCs^67^, ^68^ that >90 mg morphine‐equivalent might be a critical daily dose (in cancer patients). Of note, one PC^58^ found an inverse dose–response relationship. In other evidence‐based works, this was consistently interpreted as meaning that (insufficiently treated) acute pain can induce delirium^8^, ^9^, ^11^ which we agree upon. The NICE delirium‐GDG provides “low quality evidence” for the patient‐controlled administration of opioids as a risk factor for delirium, as well as increasing delirium durations.^11^ The SCCM PADIS guidelines state that opioid use has been “strongly shown *not* to alter risk of delirium occurrence” in ICU settings.^61^ Among individual agents, fentanyl, meperidine and morphine demonstrate higher risk than others (oxycodone, codeine) although associations were insignificant, with one exception: meperidine showed the highest risk. This is consistent with other evidence.^9^, ^11^ Pharmacokinetically, meperidine is metabolized to normeperidine, which exhibits anticholinergic properties, crosses the blood–brain barrier and may accumulate in impaired renal function.^5^, ^69^ Hypothetically, these factors might explain the increased deliriogenic potential of meperidine. GRADE evidence for individual agents was all estimated as “very low.” In conclusion, evidence for opioids as an independent risk factor for delirium remains inconsistent, but high doses (possible threshold: 90 mg morphine‐equivalent) and certain agents (e.g., meperidine) seem to be a trigger.

Polypharmacy–especially the use of multiple psychoactive drugs ‐ was described as a major delirium risk factor (4, 5 fold) by Inouye et al.^2^ Hypothetically, it is likely that multiple concurrent drugs can lead to delirium through possible pharmacokinetic and pharmacodynamic drug interactions or significant underlying comorbidities.^5^ This is not, however, well supported by strong empirical evidence (adjusted for confounders). Our meta‐analysis shows a statistically insignificant result. This changed in a sensitivity analysis and might be explained by confounding, but also by different definitions of polypharmacy (≥3, ≥5 vs. >7 concurrent drugs). Further research should be based on a more standardized definition, control for confounders and observing drug–drug interactions.

The GRADE quality of evidence ratings for all other investigated medication classes and agents were of a very low level. The following are therefore not regarded as playing a major role as *single* triggers for delirium (although a remaining deliriogenic potential cannot be ruled out): ACE‐inhibitors; antibiotics, oral anticoagulants, antiparkinsonian drugs, beta‐blockers, calcium‐channel blockers, digoxin, diuretics (as general class), H_2_‐antihistamines, heparin, Insulin, NSAIDs, paracetamol and propofol. Notably, Clegg and Young describe dihydropyridines (nifedipine) as showing a “small to moderate” risk (“low quality evidence” of one PC),^9^, ^57^ however, our GRADE‐rating needed to be downgraded to “very low.”

### 4.1 Strengths and limitations

This systematic review on delirium‐associated medication is the first work pooling individual studies and providing meta‐analyses according to medication classes and individual agents. Another strength lies in comprehensively grading the quality of evidence in a structured, transparent and standardized manner. We also compared our results to previous reviews and delirium guidelines.^11^, ^61^

Although focusing on multivariable adjusted effect measures, there are limitations to evidence levels due to heterogeneous clinical settings, broad ranges of sample sizes and delirium rates, and lack of à priori power calculations, frequently resulting in insufficient OIS and imprecision estimation. The accounted covariates in multivariable regression analyses were inconsistent across studies and might affect comparability. The number of pooled studies included in meta‐analyses was too low to conduct funnel‐plots and estimate publication biases. Negative results were not reported systematically across the studies included.

### 4.2 Implications

This critical appraisal of current evidence on delirium‐associated medication has both clinical and research implications.

Although the overall evidence is of low quality we suggest the following:

- Haloperidol (up to 6 mg/d) and olanzapine (10 mg) do not increase the risk of delirium (moderate to high quality evidence). Nevertheless, there is insufficient evidence for the use of antipsychotics as pharmacologic prevention or treatment.
- Corticosteroids and ketamine (in subanesthetic dosage: up to 1 mg/kg) did not exhibit significantly increased delirium risk (moderate quality evidence).
- Anticholinergics–whenever possible–should be de‐prescribed or avoided. There is low quality evidence for a three‐fold increased delirium risk.
- Opioids show a dose‐dependent increased delirium risk (very low quality evidence), and insufficiently treated acute pain seems to be a strong precipitating factor. We suggest avoiding meperidine. Oxycodone seems to have a favorable profile. Opioids do not seem to be a risk factor in the ICU‐setting.
- Exposure to benzodiazepines can dose‐dependently increase the risk of delirium, but evidence is of very low quality in non‐ICU‐settings. In ICU‐settings, there is strong evidence for increased risk (especially when administered for sedation and in high dosages). Lorazepam exhibited a higher risk compared to other agents (e.g., midazolam), and showed a strong dose‐effect relationship, but quality of evidence is too low to draw clinical conclusions. We therefore suggest using non‐benzodiazepine sedatives (e.g., propofol or dexmedetomidine in ICU‐settings)^61^ and avoiding oversedation.
- H_1_‐antihistamines–especially diphenhydramine–seem to increase delirium risk, but evidence remains inconsistent and of very low quality.
- Polypharmacy might strongly contribute to delirium but there is a lack of empirical evidence.
- We recommend prospective study designs for future research, with *continuous* delirium assessment and adequate follow‐up time, sufficient statistical power calculations, predefined confounders (i.e., age, dementia, comorbidities, illness severity), standardized dosages, and the sub‐categorization of delirium in the context of suspected drug‐induced pathophysiology (i.e., anticholinergic toxicity in pre‐existing cholinergic vulnerability).

## 5 CONCLUSIONS

Meta‐analyses and GRADE certainty of evidence confirm the paucity of high‐quality studies. In summary, antipsychotics, corticosteroids and ketamine (in subanesthetic dosage) did not demonstrate increased delirium risk with moderate to high certainty evidence. Low quality evidence suggests a three‐fold increased risk with primary anticholinergics. Medication classes investigated in OS (e.g., opioids, benzodiazepines, H_1_‐antihistamines, antidepressants) did not reach a reliable quality for drawing conclusions. Nevertheless, a synopsis including other evidence‐based works and guidelines shows that benzodiazepines are a strong dose‐dependent risk factor in the ICU. In non‐ICU‐settings, however, the evidence remains unclear. Opioids (especially meperidine) can trigger delirium in a dose‐dependent manner, but insufficient analgesia in acute pain is a precipitating risk factor per se. In ICU‐settings, opioids do not seem to significantly contribute to delirium. H_1_‐antihistamines (diphenhydramine) seem to increase delirium risk although the evidence is very low. Polypharmacy might be a major condition, but empirical evidence is unclear. Medication reviews, avoiding and de‐prescribing possibly deliriogenic medication in people at risk is crucial in clinical practice. This review may help clinicians to prevent drug‐induced delirium.

## AUTHOR CONTRIBUTIONS

Michael Reisinger, Daniela Schoberer, and Eva Z. Reininghaus formulated the study design. Michael Reisinger pre‐registered the study protocol. Michael Reisinger and Johanna De Biasi conducted literature searches independently. Daniela Schoberer was consulted in case of disagreement on eligibility. Michael Reisinger extracted data, Johanna De Biasi checked for accuracy. Michael Reisinger and Daniela Schoberer contributed to data analysis, meta‐analyses and interpretation. Michael Reisinger wrote the first draft of the manuscript and prepared figures and tables. Daniela Schoberer, Eva Z. Reininghaus and Frederike T. Fellendorf edited the manuscript.

## FUNDING INFORMATION

This study did not receive financial or nonfinancial support from any funding agency in the public, commercial, or nonprofit sectors.

## CONFLICT OF INTEREST

We declare no competing interests.

### PEER REVIEW

The peer review history for this article is available at https://publons.com/publon/10.1111/acps.13505.

## Supporting information


**Appendix S1** Supporting Information.

**Appendix S2** Supporting Information.

**Appendix S3** Supporting Information.

**Appendix S4** Supporting Information.

## Data Availability

The data that supports the findings of this study are available in the supplementary material of this article
