# Supplementary material for: Delirium‐associated medication in people at risk: A systematic update review, meta‐analyses, and GRADE‐profiles
Source: Acta Psychiatr Scand. 2022 Oct 11;147(1):16–42. doi: 10.1111/acps.13505 (PMC10092229; doi:10.1111/acps.13505)
Supplement: Supplementary file 1 — Appendix S1 Supporting Information. [file ACPS-147-16-s004.docx]

**Appendix – medication association of single studies**

Uni- and multivariable effect estimates and raw data of single studies

|  |  |  | **Exposure** | | **Non-exposure** | | **Univariable Analysis** |  | **Multivariable Analysis** |  |
| --- | --- | --- | --- | --- | --- | --- | --- | --- | --- | --- |
| **Medication class & study ID** | **Agents** | **Setting** | **Delirious** | **Total** | **Delirious** | **Total** | **Unadjusted effect measure (95%-CI); p-value** | **Extracted (ex) / calculated (calc)** | **Adjusted effect measure (95%-CI); p-value** | **Statistical method & adjustment for covariates** |
| **ANTICHOLINERGICS** | |  |  |  |  |  |  |  |  |  |
| Hongyu et al.; 2019 | **Penehyclidine hydrochloride (PHC)** | Surgical (thoracoscopic surgery) | 16 | 30 | 5 | 30 | **Calc OR 5.71 (1.72 – 18.94); p = 0.004** | calc |  | RCT (intention to treat [ITT] analysis)  qui-square test |
| Hongyu et al.; 2019 | **Atropine** | Surgical (thoracoscopic surgery) | 7 | 30 | 5 | 30 | **Calc OR 1.52 (0.42 – 5.47); p = 0.52** | calc | **-** | RCT (intention to treat [ITT] analysis)  qui-square test |
| Hongyu et al.; 2019 | Both **PHC** & **Atropine** (together) | Surgical (thoracoscopic surgery) | 23 | 60 | 5 | 30 | **Calc OR 3.11 (1.04 – 9.26); p = 0.04** | calc | **-** | RCT (intention to treat [ITT] analysis)  qui-square test |
| Perez-Ros et al.; 2019 | Anticholinergics as medication class; agents not specified | Nursing homes | 28 | 89 | 55 | 354 | **OR 2.47 (1.45–4.22)**  **p <0.001** | ex | **aOR 1.87 (0.95 - 3.69); p = 0.07** | Binary logistic regression analysis  adjusted for dementia, neuroleptics, falls (all variables in bivariate analysis with p-value < 0.25 and elimination of variables with an adjusted effect of <10% or with lack of improved standard error of the estimate on adjusting the model without such variables) |
| Slor et al.; 2011 | **Atropine** or **Ipratropium** | Surgical (hip surgery) | 8 | 46 | 52 | 480 | **Calc OR 1.73 (0.77 – 3.91); p = 0.19** | calc | **-** | Univariable |
| Burry et al.; 2017 | High anticholinergic potency agents (due to Duran et al. 2013)  (e.g. **Diphenhydramine, Dimenhydrinate,**  **Ipratropium, Atropine, Scopolamine**)  Exposure in the 48h prior to the development of delirium | Mixed ICU | - | - | - | - | **HR 1.05 (0.58–1.91); p-value not reported** | ex | **aHR 2.45 (1.08–5.54);**  **p-value not reported** | Multivariable Cox regression model  adjusted for age, APACHE II score on admission, smoking, history of significant alcohol consumption, history of hypertension, presence of pre-existing neurologic condition (e.g., dementia, stroke, neuromuscular disease, seizure disorder), ICU admission type (e.g., surgery), and mechanical ventilation |
| Burry et al.; 2017 | Low anticholinergic potency (due to Duran et al. 2013)  (e.g. **Ranitidine, Trazodone,**  **Olanzapine, Risperidone, and Haloperidol**)  Exposure in the 48h prior to the development of delirium | Mixed ICU | - | - | - | - | **HR 0.94 (0.65–1.36); p-value not reported** | ex | **aHR 0.83 (0.47–1.48);**  **p-value not reported** | Multivariable Cox regression model  adjusted for age, APACHE II score on admission, smoking, history of significant alcohol consumption, history of hypertension, presence of pre-existing neurologic condition (e.g., dementia, stroke, neuromuscular disease, seizure disorder), ICU admission type (e.g., surgery), and mechanical ventilation |
| Schor et al.; 1992 | Anticholinergics as medication class; agents not specified | General Medicine / Surgery | 21 | 83 | 70 | 208 | **Calc OR 0.67 (0.38- 1.18); p = 0.17** | Unadjusted OR**-calc**  Adjusted OR**-ex** | **aOR 0.76 (0.41-1.43);**  **p-value not reported** | Multivariable logistic regression analysis adjust ed for age and sex |
| Gustafson et al.; 1988 | Anticholinergics as medication class; agents not specified | Orthopedics Surgical (hip surgery) | 33 | 39 | 35 | 72 | **Calc RR 1.74 (1.33 – 2.29); p = 0.0001** | Unadjusted RR-**calc**  Adjusted estimate**-ex** | **Standardized or beta coefficient 0.181**  **p = 0.0851** | Multiple linear regression analysis  adjusted for all variables significant (p < 0.05) in chi-square test (age, dementia, depression, cerebrovascular disease, cardiovascular disease, drugs with anticholinergic effect) |
| **ANTIDEPRESSANTS** | |  |  |  |  |  |  |  |  |  |
| Perez-Ros et al.; 2019 | Antidepressants as medication class; agents not specified | Nursing homes | - | - | - | - | **OR 1.36 (0.83–2.24)**  **p = 0.224** | ex | - | Univariable |
| Brown et al.; 2016 | Antidepressants as medication class; agents not specified | Surgical (spine surgery) | 16 | 26 | 20 | 63 | **Calc OR 3.44 (1.33 – 8.91); p = 0.01** | Unadjusted OR - **calc**  Adjusted OR - **ex** | **aOR 4.70 (1.03 – 21.5)**  **p = 0.046** | Multivariable model (forward stepwise regression model)  adjusted for age, functional status (acts of daily living), American Society of Anesthesiologists (ASA) risk score, surgery length, intraoperative red blood cell transfusion, return to the operating room, and any complication |
| Burry et al.; 2017 | Antidepressants as medication class; agents not specified | Mixed ICU | 40 | 75 | 220 | 445 | **Calc OR** 1.17 (0.72 – 1.91); p = 0.53 | calc | - | Univariable |
| Gustafson et al.; 1988 | Antidepressants as medication class; agents not specified  (according to Clegg & Young; 2011 = *tricyclic antidepressants*) | Orthopedics Surgical (hip surgery) | 14 | 14 | 54 | 97 | **Calc OR** 4.3167 (1.6062 – 11.6007); p = 0.0037  **Calc RR** 1.59 (1.22 – 2.10); p = 0.0007 | calc | - | Univariable |
| **ANTIPSYCHOTICS** | |  |  |  |  |  |  |  |  |  |
| Perez-Ros et al.; 2019 | Antipsychotics as medication class; agents not specified | Nursing homes | 31 | 102 | 52 | 341 | **OR 2.43 (1.45–4.09); p <0.001** | **ex** | **aOR 2.39 (1.23 -4.65);**  **p <0.001** | Binary logistic regression analysis  adjusted for dementia, neuroleptics, falls (all variables in bivariate analysis with p-value < 0.25 and elimination of variables with an adjusted effect of <10% or with lack of improved standard error of the estimate on adjusting the model without such variables) |
| Burry et al.; 2017 | Antipsychotics as medication class; agents not specified | Mixed ICU | 159 | 215 | 101 | 305 | **Calc OR 5.73 (3.90 – 8.44); p < 0.0001** | calc | - | Univariable |
| Larsen et al.; 2010 | 10 mg **Olanzapine** perioperatively | Surgical (knee or hip replacement surgery) | 28 | 196 | 82 | 204 | **Calc OR 0.25 (0.15 – 0.40); p < 0.0001**  **Ex OR 0.3 (0.2–0.4); p < 0.001** | calc & ex | **aOR 0.2 (0.1–0.4);**  **p < 0.001** | RCT (per protocol [PP]analysis)  Multiple logistic regression including all variables with univariate p values < 0.10 |
| Schrijver et al.; 2018 | 2mg **Haloperidol** (for 7 days) | Mixed medical and surgical (acutely hospitalized patients) | 23 | 118 | 18 | 124 | **Calc OR 1.43 (0.73 – 2.80); p = 0.304**  **Ex OR 1.43 (0.72, 2.78); p = 0.302** | calc & ex | - | RCT (intention to treat [ITT] analysis)  Comparison of delirium incidence using chi-squared test |
| Van den Boogaard et al.; 2018  **ITT-analysis** | 6mg **Haloperidol** vs placebo daily (median 2 days) | 21 ICUs | 244 | 732 | 233 | 707 | **Calc RR 1.01 (0.87 – 1.17); p = 0.88** [ITT]  **Ex:**  proportion difference: 0.4% (- 4.6% to 5.4%) [ITT analysis]  mean difference:  1.5% (- 3.6% to 6.7%) [PP analysis] | calc (from ITT-analysis) & ex (from both) | - | RCT (intention to treat [ITT] and per protocol [PP] analysis)  Delirium incidence comparison (as categorical or binary variable) was calculated using chi-squared test |
| Van den Boogaard et al.; 2018  **PP-analysis** | 6mg i.v. **Haloperidol** vs placebo daily (median 2 days) | 21 ICUs | 233 | 682 | 218 | 668 | **Calc RR 1.05 (0.90 – 1.22); p = 0.55** [PP]  **Ex:**  proportion difference: 0.4% (- 4.6% to 5.4%) [ITT analysis]  mean difference:  1.5% (- 3.6% to 6.7%) [PP analysis] | calc (from PP-analysis) & ex (from both) | - | RCT (intention to treat (ITT) and per protocol (PP) analysis)  Delirium incidence comparison (as categorical or binary variable) was calculated using chi-squared test |
| Wang et al.; 2012 | 1.7mg i.v. **Haloperidol** vs placebo (for 12hrs) | 2 Surgical ICUs | 35 | 229 | 53 | 228 | **Calc OR 0.595 (0.371 – 0.956); p = 0.032** | Unadjusted OR- calc  Adjusted OR - ex | **aOR 0.574 (0.352 – 0.937); p = 0.026** | RCT (intention to treat (ITT) analysis  Multivariable logistic regression model adjusted for variables with a p-value < 0.10 in univariable analysis |
| Al-Qadheeb et al., 2016 | 4 mg i.v. **Haloperidol** vs placebo | 3 Mixed ICUs (medical and surgical)  [Mechanically ventilated patients] | 12 | 34 | 8 | 34 | **Calc OR 1.77 (0.61 – 5.11); p = 0.29** | **calc** | - | RCT (intention to treat (ITT) analysis)  Outcomes were compared using the Mann-Whitney *U* test, the χ² test or Fisher exact tests |
| Schor et al.; 1992 | Antipsychotics as medication class; agents not specified | General Medicine / Surgery | 19 | 38 | 72 | 253 | **Calc OR 2.51 (1.26 – 5.02); p = 0.009** | Unadjusted OR- calc  Adjusted OR - ex | **aOR 4.48 (1.82-10.45); p < 0.01** | Stepwise logistic regression analysis  adjusted for all variables with age- and sex-adjusted values less than 0.10 were entered in the model (prior cognitive impairment, fracture on admission, age > 80a, infection, narcotic use, male sex) |
| Gustafson et al.; 1988 | Antipsychotics as medication class; agents not specified | Orthopedics Surgical (hip surgery) | 28 | 34 | 40 | 77 | **Calc RR** **1.59 (1.22 – 2.10); p = 0.0007** | calc | - | Univariable |
| Kalisvaart et al.; 2005 | **Haloperidol** 1.5 mg/d  (on admission until POD 3) | Orthopedics (hip surgery) | 32 | 212 | 36 | 218 | **Calc RR 0.91 (0.59 – 1.42); p = 0.69**  **Ex RR 0.9 (0.6- 1.3); no p-value stated** | calc & ex | - | RCT **(**intention to treat (ITT) analysis)  Parametric values (Student t test) and nonparametric values (Mann-Whitney U test) |
| Pandharipande et al.; 2006 | Antipsychotics as medication class **(Haloperidol** or **Olanzapine)**  (ICU-administration) | ICU – mechanically ventilated patients | 66 | 75 | 102 | 123 | **Calc OR 1.51 (0.65 – 3.50); p = 0.336** | calc | **No effect estimate reported**; **p = 0.39** | Multivariable Markov regression model  adjusted for age, sex, visual and hearing deficits, history of dementia, depression, severity of illness (modified APACHE II-score), sepsis, history of neurologic  disease, hematocrit, and daily serum glucose concentrations |
| **ACE-INHIBITORS** | |  |  |  |  |  |  |  |  |  |
| Brown et al.; 2016 | ACE-inhibitors as medication class; agents not specified | Surgical (spine surgery) | 11 | 22 | 25 | 67 | **Calc OR 1.68 (0.64 – 4.44); p = 0.30** | calc | - | Univariable |
| **ANTIPARKINSONIAN DRUGS** | |  |  |  |  |  |  |  |  |  |
| Gustafson et al.; 1988 | Antiparkinsonian drugs as medication class; agents not specified | Orthopedics Surgical (hip surgery) | 12 | 16 | 56 | 95 | **Calc RR** **1.27 (0.92 – 1.77); p = 0.15** | calc | - | Univariable |
| **ANTICOAGULANTS** | |  |  |  |  |  |  |  |  |  |
| Pasinska et al.; 2018 | Anticoagulants as medication class; agents not specified | Stroke unit | 24 | 172 | 34 | 488 | **OR 2.17 (1.24–3.77)**  **p = 0.006** | ex | - | Univariable logistic regression |
| **BETA BLOCKERS** | |  |  |  |  |  |  |  |  |  |
| Brown et al.; 2016 | Beta-blockers as medication class; agents not specified | Surgical (spine surgery) | 16 | 35 | 20 | 54 | **Calc RR 1.23 (0.75 – 2.04); p = 0.41** | calc | **-** | Univariable |
| Burry et al.; 2017 | Beta-blockers as medication class; agents not specified  (exposure in the 48h prior to the development of delirium) | Mixed ICU | 136 | 220 | 124 | 300 | **HR 0.94 (0.56 - 1.56); p-value not reported** | ex | **aHR 1.26 (0.66–2.42);**  **p-value not reported** | Multivariable Cox regression model  adjusted for age, APACHE II score on admission, smoking, history of significant alcohol consumption, history of hypertension, presence of pre-existing neurologic condition (e.g., dementia, stroke, neuromuscular disease, seizure disorder), ICU admission type (e.g., surgery), and mechanical ventilation |
| **HEPARIN** | |  |  |  |  |  |  |  |  |  |
| Pasinska et al.; 2018 | **Heparin** | Stroke unit | 13 | 168 | 15 | 488 | **OR 2.64 (1.23–5.69)**  **p = 0.013** | ex | - | Univariable logistic regression |
| **INSULIN** | |  |  |  |  |  |  |  |  |  |
| Pasinska et al.; 2018 | **Insulin** | Stroke unit | 24 | 171 | 33 | 488 | **OR 2.25 (1.29–3.94)**  **p = 0.004** | ex | - | Univariable logistic regression |
| **DIURETICS** | |  |  |  |  |  |  |  |  |  |
| Pasinska et al.; 2018 | Diuretics as medication class; agents not specified | Stroke unit | 54 | 168 | 103 | 488 | **Calc OR 1.77 (1.20–2.62); p = 0.004** | calc | - | Univariable logistic regression |
| **CALCIUM CHANNEL BLOCKERS** | |  |  |  |  |  |  |  |  |  |
| Brown et al.; 2016 | Ca-channel-blockers as medication class; agents not specified | Surgical (spine surgery) | 11 | 23 | 25 | 66 | **Calc OR 1.50 (0.58 – 3.92); p = 0.404** | calc | - | Univariable |
| Van der Mast et al.; 1999 | **Nifedipine** | Cardiac surgery | 11 | 53 | 29 | 243 | **Calc OR 1.93 (0.90 – 4.17); p = 0.09** | Unadjusted OR-**calc**  Adjusted OR-**ex** | **aOR 2.4 (1.0–5.8);**  **p = 0.047** | Multivariable backward stepwise logistic regression analysis  adjusted for age >65 years; Inclusion as an in-patient; Use of nifedipine; MMSE score <23; GHQ score .7; DAL score .6; Albumin <40 g/l; Ratio rT3:T3 >0.1450; Ratio Phe:oLNAA >13.35 |
| **ANTIBIOTICS** | |  |  |  |  |  |  |  |  |  |
| Limpawattana et al.; 2016 | Antibiotics as medication class; agents not specified | Medical ICU | 8 | 18 | 36 | 81 | **OR 1.00 (0.36 -2.79); p = 1** | **ex** | - | Univariable |
| **ANTIHISTAMINES** | |  |  |  |  |  |  |  |  |  |
| Limpawattana et al.; 2016 | Antihistamines as medication class; agents not specified | Medical ICU | 5 | 15 | 39 | 84 | **OR 0.58 (0.18 – 1.83); p = 0.35** | **ex** | - | Univariable |
| 1. **HISTAMINE H_1_-RECEPTOR-ANTAGONISTS** | | |  |  |  |  |  |  |  |  |
| Cole et al.; 2016 | H_1_-antihistamines as medication class  **(**agents**: Dimenhydrinate, Hydroxyzine, Diphenhydramine, Cetirizine, Loratadine)** | Long term care facilities | 4 | 95 | 176 | 1969 | **OR 0.50 (0.18 - 1.39), p = not reported** | ex | **aOR 0.31 (0.11 - 0.87); p-value not reported** | Conditional logistic regression adjusted for antipsychotic use and dementia |
| Cole et al.; 2016 | **Diphenhydramine** | Long term care facilities | 1 | 42 | 94 | 2022 | **OR 0.52 (0.07 – 3.68), p = 0.50** | ex | **aOR 0.67 (0.09 – 4.99); p = 0.70** | Conditional logistic regression adjusted for antipsychotic use and dementia |
| Marcantonio et al.; 1994 | **Diphenhydramine** | Mixed surgical (general, orthopedic surgery and gynecology) | 9 | 18 | 82 | 227 | **Calc OR 1.77 (0.68 – 4.63); p = 0.25** | Unadjusted OR-**calc**  Adjusted OR-**ex** | **aOR 1.8 (0.7 - 4.5);**  **p = 0.22** | Matched analysis: conditional logistic regression model controlled for exposure to all other drugs within that class. |
| 1. **HISTAMINE H_2_-RECEPTOR-ANTAGONISTS** | | |  |  |  |  |  |  |  |  |
| Schor et al.; 1992 | H_2_-antihistamines as medication class; agents not specified | General Medicine / Surgery | 41 | 121 | 50 | 170 | **Calc OR 1.23 (0.75 – 2.03); p = 0.42** | Unadjusted OR-**calc**  Adjusted OR-**ex** | **aOR 1.42 (0.81-2.47);**  **p-value not reported** | Multivariable logistic regression analysis adjusted for age and sex |
| **CARDIAC GLYCOSIDES** | |  |  |  |  |  |  |  |  |  |
| Schor et al.; 1992 | **Digoxin** | General Medicine / Surgery | 19 | 73 | 72 | 218 | **Calc OR 0.71 (0.39 – 1.29); p = 0.27** | Unadjusted OR-**calc**  Adjusted OR-**ex** | **aOR 0.52 (0.30-0.90);**  **p-value not reported** | Multivariable logistic regression analysis adjusted for age and sex |
| **CORTICOSTEROIDS** | |  |  |  |  |  |  |  |  |  |
| Cole et al.; 2016 | **Prednisone** | long term care facilities | 2 | 95 | 63 | 1969 | **OR 0.67 (0.16 - 2.80); p = not reported** | ex | **aOR 0.89 (0.21 - 3.74); p-value not reported** | Conditional logistic regression adjusted for antipsychotic use and dementia |
| Brown et al.; 2016 | Intraoperative exposure:   - **Dexamethasone** (4 – 20 mg) - **Hydrocortisone** (100 – 200 mg) | Surgical (spine surgery) | 10 | 40 | 26 | 49 | **Calc OR 0.29 (0.12 – 0.73); p = 0.008** | calc | - | Univariable |
| Limpawattana et al.; 2016 | Corticosteroids as medication class; agents not specified | Medical ICU | 13 | 23 | 31 | 76 | **OR 1.88 (0.74 - 4.84); p = 0.19** | ex | - | Univariable |
| Schreiber et al.; 2014 | Corticosteroids as medication class; agents not specified | Mechanically ventilated ICU patients | - | - | - | - | Bivariable regression:  **OR 1.48 (1.05 - 2.08); p = 0.03** | ex | **aOR 1.52 (1.05 - 2.21); p = 0.03** | Multivariable logistic regression (first-order Markov model)  adjusted for demographics (age, race, sex), individual covariates (p ≤ 0.2 in bivariable analysis); predefined baseline covariates (e.g. severity of illness score, ICU covariates, other medication classes etc.) |
| Wolters et al.; 2015 | Corticosteroids as medication class **(Dexamethasone**, **Fludrocortisone**,  **Hydrocortisone**, **Methylprednisolone**, or **Prednisone)** | Mixed (medical & surgical) ICU | 284 | 513 | 251 | 599 | **Ex OR 1.12 (0.93–1.35); p-value not reported** (CAVE: daily transition to delirium)  **Calc OR 1.72 (1.36 – 2.18); p < 0.00001** | Unadjusted OR-**calc & ex**  Adjusted OR-**ex** | **aOR 1.08 (0.89–1.32); p-value not reported** | First-order Markov multinomial logistic regression model  adjusted for age, corticosteroid use prior to ICU admission, Charlson Comorbidity Index, type of ICU admission, APACHE IV score, length of ICU stay, SOFA score, use of mechanical ventilation, presence of inflammation, use of opioids and benzodiazepines |
| Burry et al.; 2017 | Corticosteroids as medication class; agents not specified  (exposure in the 48h prior to the development of delirium) | Mixed ICU | 118 | 230 | 142 | 290 | **HR 0.75 (0.49 - 1.15); p-value not reported** | ex | **aHR 0.74 (0.42 - 1.33); p-value nor reported** | Multivariable Cox regression model  adjusted for age, APACHE II score on admission, smoking, history of significant alcohol consumption, history of hypertension, presence of pre-existing neurologic condition (e.g., dementia, stroke, neuromuscular disease, seizure disorder), ICU admission type (e.g., surgery), and mechanical ventilation |
| Clemmesen et al., 2018 | 125 mg iv **Methylprednisolone** preoperatively | Surgical (hip fracture patients) | 10 | 59 | 19 | 58 | **Calc OR 0.42 (0.17 – 1.00); p = 0.0509**  **Ex OR (**non-exposure vs exposure**) 2.39 (1.00 – 5.72) ; p = 0.048**  **NNT = 7** | calc & ex | - | RCT (intention to treat (ITT) analysis)  Postoperative delirium incidence (secondary outcome) was analyzed between study groups using χ² test |
| Sauër et al., 2014 | 1 mg/kg iv **Dexamethasone** intraoperatively  (maximal dose: 100 mg) | Surgical (cardiac surgery) | 52 | 367 | 55 | 370 | **OR 0.95 (0.63 – 1.43); p = 0.79** | ex | **aOR** **0.85 (0.55 – 1.31); p = 0.45** | RCT (intention to treat (ITT) analysis)  Logistic regression analysis adjusted for baseline variables (age, gender, valve surgery, and history of stroke) |
| Schor et al.; 1992 | Corticosteroids as medication class; agents not specified | General Medicine / Surgery | 4 | 24 | 87 | 267 | **Calc OR 0.41 (0.14 – 1.25); p = 0.12** | Unadjusted OR-**calc**  Adjusted OR-**ex** | **aOR 0.51 (0.16 - 1.67);**  **p-value not reported** | Multivariable logistic regression analysis adjusted for age and sex |
| **Dose comparisons** | |  |  |  |  |  |  |  |  |  |
| Schreiber et al.; 2014 | **> 40 mg Prednisone-equivalent** in 24h | Mechanically ventilated ICU patients | - | - | - | - | Bivariable regression:  **OR 1.03 (0.94–1.13); p = 0.56** | ex | **aOR 0.97 (0.89 – 1.07);**  **p = 0.57** | Multivariable logistic regression (first-order Markov model)  adjusted for demographics (age, race, sex), individual covariates (p ≤ 0.2 in bivariable analysis); predefined baseline covariates (e.g. severity of illness score, ICU covariates, other medication classes) |
| Wolters et al.; 2015 | **10 mg Prednisone-equivalent increase**  Agents:  **Dexamethasone**, **Fludrocortisone**,  **Hydrocortisone**, **Methylprednisolone** & **Prednisone** | Mixed (medical & surgical) ICU | - | - | - | - | **OR 0.99 (0.98 – 1.00);** **p-value nor reported** | Ex | **aOR 1.00 (0.99 – 1.01); p-value nor reported** | First-order Markov multinomial logistic regression model  adjusted for age, corticosteroid use prior to ICU admission, Charlson Comorbidity Index, type of ICU admission, APACHE IV score, length of ICU stay, SOFA score, use of mechanical ventilation, presence of inflammation, use of opioids and benzodiazepines |
| **OPIOIDS** | |  |  |  |  |  |  |  |  |  |
| Brown et al.; 2016 | Opioids as medication class; agents not specified | Surgical (spine surgery) | 19 | 35 | 17 | 54 | **Calc OR 2.58 (1.07 – 6.22); p = 0.03** | calc | - | Univariable |
| Sieber et al.; 2012 | Opioids as medication class; agents not specified (postoperative exposure) | Surgical (hip surgery) | 53 | 213 | 7 | 23 | **Calc OR 0.76 (0.30 – 1.94); p = 0.56** | calc | **aOR 1.27 (0.35, 4.68);**  **p = 0.711**  (exposure on POD 1 in regard to incident delirium on POD 2) | Multivariate logistic regression  Adjusted for dementia status and ICU admission |
| Slor et al.; 2011 | Opioids as medication class;  Agents: **Alfentanil,**  **Morphine, Nalbuphine, Piritramide** or **Sufentanil** | Surgical (hip surgery) | 46 | 419 | 14 | 107 | **Calc OR 0.82 (0.43 – 1.55); p =0.54** | calc | - | Univariable |
| Schreiber et al.; 2014 | Opioids as medication class; agents not specified | Mechanically ventilated ICU patients | - | - | - | - | Bivariable regression:  **OR 1.16 (0.88–1.52); p = 0.29** | ex | - | Bivariable regression analysis |
| Leung et al.; 2013 | Opioids as medication class; agents not specified  (pre-operative exposure) | Surgical (non-cardiac surgery) | 79 | 171 | 155 | 410 | **Calc OR 1.41 (0.98 – 2.03); p = 0.06** | calc |  | Univariable |
| Burry et al.; 2017 | Opioids as medication class; agents not specified | Mixed ICU | 238 | 450 | 22 | 70 | **Calc OR** **2.45 (1.43-4.19); p = 0.0011** | calc | - | Univariable |
| Schor et al.; 1992 | Opioids as medication class; agents not specified | General Medicine / Surgery | 41 | 113 | 50 | 178 | **Calc OR 1.46 (0.88 – 2.41); p = 0.14** | Unadjusted OR-**calc**  Adjusted OR-**ex** | **aOR 2.54 (1.24-5.18);**  **p < 0.01** | Stepwise logistic regression analysis  adjusted for all variables with age- and sex-adjusted p-values less than 0.10 |
| Pandharipande et al.; 2006 | **Fentanyl**  (administered in the ICU) | ICU – mechanically ventilated patients | - | - | - | - | Univariable effect estimate not reported | **ex** | **aOR 1.2 (1.0–1.5);**  **p = 0.09** | Multivariable Markov regression model  adjusted for age, sex, visual and hearing deficits, history of dementia, depression, severity of illness (modified APACHE II-score), sepsis, history of neurologic disease, hematocrit, and daily serum glucose concentrations |
| Pandharipande et al.; 2006 | **Morphine**  (administered in the ICU) | ICU – mechanically ventilated patients | - | - | - | - | Univariable effect estimate not reported | **ex** | **aOR 1.1 (0.9–1.2);**  **p = 0.24** | Multivariable Markov regression model  adjusted for age, sex, visual and hearing deficits, history of dementia, depression, severity of illness (modified APACHE II-score), sepsis, history of neurologic disease, hematocrit, and daily serum glucose concentrations |
| Morrison et al.; 2003 | **Meperidine (= Pethidine)** | Orthopedics (Hip surgery) | 27 | 129 | 56 | 412 | **Calc RR 1.54 (1.02 – 2.33); p = 0.04** | Unadjusted RR-**calc**  Adjusted RR-**ex** | **aRR 2.4 (1.3 – 4.5);**  **p = 0.004** | Multiple logistic regression  adjusted for all variables with p < 0.15 in univariate analysis (e.g. age, sex, cognitive impairment, residency in a nursing home, medical complication, heart failure on admission) |
| Marcantonio et al.; 1994 | Opioids as medication class; agents not specified | Mixed surgical (general, orthopedic surgery and gynecology) | 87 | 231 | 4 | 14 | **Calc OR 1.51 (0.46 – 4.96); p = 0.50** | Univariable OR-**calc**  Matched analysis-**ex** | - | Matched analysis |
| Marcantonio et al.; 1994 | **Meperidine (= Pethidine)** | Mixed surgical (general, orthopedic surgery and gynecology) | 59 | 123 | 32 | 122 | **Calc OR 2.59 (1.52 – 4.43); p = 0.0005** | Univariable OR-**calc**  Matched analysis-**ex** | **aOR 2.7 (1.3 - 5.5);**  **p-value not reported** | Matched analysis  Conditional logistic regression model controlled for exposure to all other drugs within that class |
| Marcantonio et al.; 1994 | **Morphine** | Mixed surgical (general, orthopedic surgery and gynecology) | 22 | 74 | 69 | 171 | **Calc OR 0.63 (0.35 – 1.12); p = 0.12** | Univariable OR-**calc**  Matched analysis-**ex** | **aOR 1.2 (0.6 - 2.4);**  **p-value not reported** | Matched analysis  Conditional logistic regression model controlled for exposure to all other drugs within that class |
| Marcantonio et al.; 1994 | **Fentanyl** | Mixed surgical (general, orthopedic surgery and gynecology) | 9 | 23 | 82 | 222 | **Calc OR 1.10 (0.45 – 2.65); p = 0.84** | Univariable OR-**calc**  Matched analysis-**ex** | **aOR 1.5 (0.6 - 4.2);**  **p-value not reported** | Matched analysis  Conditional logistic regression model controlled for exposure to all other drugs within that class |
| Marcantonio et al.; 1994 | **Oxycodon** | Mixed surgical (general, orthopedic surgery and gynecology) | 9 | 39 | 82 | 206 | **Calc OR 0.45 (0.20 – 1.01); p = 0.05** | Univariable OR-**calc**  Matched analysis-**ex** | **aOR 0.7 (0.3 - 1.6);**  **p-value not reported** | Matched analysis  Conditional logistic regression model controlled for exposure to all other drugs within that class |
| Marcantonio et al.; 1994 | **Codeine** | Mixed surgical (general, orthopedic surgery and gynecology) | 6 | 17 | 85 | 228 | **Calc OR 0.92 (0.33 – 2.57); p = 0.87** | Univariable OR-**calc**  Matched analysis-**ex** | **aOR 1.1 (0.4 - 3.6);**  **p-value not reported** | Matched analysis  Conditional logistic regression model controlled for exposure to all other drugs within that class |
| **Dose comparisons** | |  |  |  |  |  |  |  |  |  |
| Leung et al.; 2013 | Postoperative **Hydromorphone-equivalents** > 8 mg vs < 8 mg (with high pain and high delirium risk scores) **#** | Surgical (non-cardiac surgery) | - | - | - | - | Univariable effect estimate not reported | ex | **aOR 3.43 (1.67 – 7.03); p = 0.0008** | Multivariable logistic regression with risk stratification adjusted for 5 risk factors with p-values ≤ 0.20 in bivariate analysis (including age, cognitive impairment, surgical type and risk) |
| Leung et al.; 2013 | Postoperative **Hydromorphone-equivalents** > 8 mg vs < 8 mg (with low pain and delirium risk scores) **##** | Surgical (non-cardiac surgery) | - | - | - | - | Univariable effect estimate not reported | ex | **aOR 2.60 (1.31 – 5.17);**  **p = 0.006** | Multivariable logistic regression with risk stratification adjusted for 5 risk factors with p-values ≤ 0.20 in bivariate analysis (including age, cognitive impairment, surgical type and risk) |
| Burry et al.; 2017 | **25 mcg Fentanyl-equivalents increment**  Exposure in the 48h prior to the development of delirium | Mixed ICU | - | - | - | - | **HR 1.09 (0.75–1.58); p-value not reported** | **ex** | **HR 1.00 (0.99–1.07);**  **p-value nor reported** | Multivariable Cox regression model  adjusted for age, APACHE II score on admission, smoking, history of significant alcohol consumption, history of hypertension, presence of pre-existing neurologic condition (e.g., dementia, stroke, neuromuscular disease, seizure disorder), ICU admission type (e.g., surgery), and mechanical ventilation |
| Morrison et al.; 2003 | **Morphine sulfate equivalents < 10mg** | Orthopedics (Hip surgery) | 56 | 204 | 31 | 337 | **Calc RR 3.00 (2.00 – 4.50); p < 0.001** | Unadjusted RR-**calc**  Adjusted RR-**ex** | **aRR 5.4 (2.4 – 12.3);**  **p < 0.001** | Multiple logistic regression  adjusted for all variables with p < 0.15 in univariate analysis (e.g. age, sex, cognitive impairment, residency in a nursing home, medical complication, heart failure on admission) |
| Morrison et al.; 2003 | **Morphine sulfate equivalents 10 – 30 mg** | Orthopedics (Hip surgery) | 21 | 192 | 66 | 349 | **Calc RR 0.58 (0.37 – 0.91); p = 0.02** | UnadjustedRR-**calc**  Adjusted RR-**ex** | **aRR 1.4 (0.6 – 3.3);**  **p = 0.4** | Multiple logistic regression  adjusted for all variables with p < 0.15 in univariate analysis (e.g. age, sex, cognitive impairment, residency in a nursing home, medical complication, heart failure on admission) |
| Morrison et al.; 2003 | **Morphine sulfate equivalents > 30 mg** | Orthopedics (Hip surgery) | 10 | 145 | 77 | 396 | **Calc RR 0.35 (0.19 – 0.67); p < 0.001** | calc | - | Univariable |
| **NON-OPIOID ANALGETICS** | |  |  |  |  |  |  |  |  |  |
| 1. **NSAIDs** | |  |  |  |  |  |  |  |  |  |
| Schor et al.; 1992 | NSAIDs as medication class; agents not specified | General Medicine / Surgery | 3 | 19 | 88 | 272 | **Calc OR 0.39 (0.11 – 1.38); p = 0.14** | Unadjusted OR-**calc**  Adjusted OR-**ex** | **aOR 0.39 (0.10-1.49);**  **p-value not reported** | Multivariable logistic regression analysis adjusted for age and sex |
| Cole et al.; 2016 | **Aspirin** | Long term care facilities | 37 | 826 | 58 | 1195 | **OR 0.95 (0.62; 1.45); p-value not reported** | ex | **aOR 1.07 (0.70 - 1.65); p-value not reported** | Conditional logistic regression adjusted for antipsychotic use and dementia |
| Cole et al.; 2016 | **Naproxen** | Long term care facilities | 1 | 22 | 94 | 2042 | **OR 1.13 (0.15; 8.44); p-value not reported** | ex | **aOR 0.71 (0.10 - 5.33); p-value not reported** | Conditional logistic regression adjusted for antipsychotic use and dementia |
| 1. **ACETAMINOPHEN (= PARACETAMOL)** | | |  |  |  |  |  |  |  |  |
| Cole et al.; 2016 | **Acetaminophen = Paracetamol** | Long term care facilities | 42 | 95 | 947 | 1969 | **OR 0.70 (0.45; 1.08); p-value not reported** | ex | **aOR 0.81 (0.52 - 1.26); p-value not reported** | Conditional logistic regression adjusted for antipsychotic use and dementia |
| **“INFLAMMATION ANTAGONIST MEDICATION”** | | |  |  |  |  |  |  |  |  |
| Cole et al.; 2016 | Histamine-1-receptor-antagonists, steroids, NSAIDs, Acetaminophen (= Paracetamol)   - **calculated as “class”** | Long term care facilities | 60 | 95 | 1469 | 1969 | **OR 0.53 (0.34; 0.81); p-value not reported** | ex | **aOR 0.60 (0.38 - 0.92); p-value not reported** | Conditional logistic regression adjusted for antipsychotic use and dementia |
| **POLYPHARMACY** | |  |  |  |  |  |  |  |  |  |
| Perez-Ros et al.; 2019 | **>7 medications** | Nursing homes | 66 | 348 | 17 | 95 | **OR 1.07 (0.59–1.93)**  **p = 0.812** | ex | - | Univariable |
| Saljuqi et al.; 2020 | **≥ 3 medications** | Surgical (emergency general surgery) | 11 | 30 | 27 | 115 | **Calc OR 1.89 (0.80 – 4.45); p = 0.15** | Unadjusted OR-**calc**  Adjusted OR-**ex** | **aOR 1.3 (1.1 – 1.4)**  **P < 0.01** | Multivariable logistic regression analysis  adjusted for all variables significant on univariable level (demographics [e.g. age], vital parameters on admission, comorbidities, laboratory parameters, ASA class and diagnosis |
| Hein et al.; 2014 | **≥ 5 medications** | Acute geriatric care unit (after emergency hospital admission) | 71 | 240 | 31 | 170 | **Calc OR 1.88 (1.17 – 3.04); p = 0.0094** | Unadjusted OR-**calc**  Adjusted OR-**ex** | **aOR 2.33 (1.23 - 4.41);**  **p = 0.010** | Forward stepwise logistic regression analysis  adjusted for dementia, age, intake of drugs known to induce delirium, severe renal impairment, and source of admission |
| **ANXIOLYTICS** | |  |  |  |  |  |  |  |  |  |
| Perez-Ros et al.; 2019 | Benzodiazepine & Non-Benzodiazepine anxiolytics as group | Nursing homes | - | - | - | - | **OR 1.01 (0.61–1.65);**  **p = 0.961** | ex | - | Univariable |
| **ANESTHETICS** | |  |  |  |  |  |  |  |  |  |
| 1. **KETAMINE** | |  |  |  |  |  |  |  |  |  |
| Avidan et al.; 2017 | **Ketamine**  (either Ketamine 0.5 or 1.0 mg/kg i.v. as bolus) | Surgical (cardiac and non-cardiac) | 85 | 437 | 43 | 217 | **Calc OR 0.98 (0.65 – 1.47); p = 0.91** | calc | - | RCT (intention to treat [ITT] analysis) |
| Perbet et al.; 2018 | **Ketamine**  (0.2 mg/kg/h continuous infusion of Ketamine) | ICU (mixed; mechanically ventilated patients) | 17 | 80 | 30 | 82 | **Calc OR 0.47 (0.23 – 0.94); p = 0.0332** | calc | - | RCT (per protocol [PP] analysis) |
| **Dose comparisons** | |  |  |  |  |  |  |  |  |  |
| Avidan et al.; 2017 | **Ketamine 0.5mg/kg intraoperatively** | Surgical (cardiac and non-cardiac) | - | - | - | - | Univariable effect estimate not reported | ex | **aOR 0.90 (0.54 - 1.50); p = 0.69** | RCT (intention to treat [ITT] analysis)  Logistic regression model controlling for known risk factors (ratio of variables to outcomes to 1:10) |
| Avidan et al.; 2017 | **Ketamine 1.0mg/kg intraoperatively** | Surgical (cardiac and non-cardiac) | - | - | - | - | Univariable effect estimate not reported | ex | **aOR 0.97 (0.59 – 1.61);**  **p = 0.91** | RCT (intention to treat [ITT] analysis)  Logistic regression model controlling for known risk factors (ratio of variables to outcomes to 1:10) |
| 1. **PROPOFOL** | |  |  |  |  |  |  |  |  |  |
| Pandharipande et al.; 2006 | **Propofol**  (ICU-administration) | ICU – mechanically ventilated patients | - | - | - | - | Univariable effect estimate not reported | ex | **aOR 1.2 (0.9–1.7);**  **p = 0.18** | Multivariable Markov regression model  adjusted for age, sex, visual and hearing deficits, history of dementia, depression, severity of illness (modified APACHE II-score), sepsis, history of neurologic disease, hematocrit, and daily serum glucose concentrations |
| **Dose comparisons** | |  |  |  |  |  |  |  |  |  |
| Burry et al.; 2017 | **Propofol, 25 mg increment**  (exposure in the 48h prior to the development of delirium) | Mixed ICU | - | - | - | - | **HR 1.38 (0.92–2.04); p-value not reported** | ex | **aHR 1.00 (0.99–1.01);**  **p-value nor reported** | Multivariable Cox regression model  adjusted for age, APACHE II score on admission, smoking, history of significant alcohol consumption, history of hypertension, presence of pre-existing neurologic condition (e.g., dementia, stroke, neuromuscular disease, seizure disorder), ICU admission type (e.g., surgery), and mechanical ventilation |
| **BENZODIAZEPINES** | |  |  |  |  |  |  |  |  |  |
| Brown et al.; 2016 | Benzodiazepines as medication class; agents not specified | Surgical (spine surgery) | 9 | 17 | 27 | 72 | **Calc OR: 1.87 (0.65 – 5.43); p = 0.24** | calc | - | Univariable |
| Limpawattana et al.; 2016 | Benzodiazepines as medication class; agents not specified | Medical ICU | 11 | 18 | 33 | 81 | **OR 2.29 (0.80 - 6.51); p = 0.12** | ex |  | Univariable |
| Sieber et al.; 2012 | **Midazolam** intraoperatively | Surgical (hip surgery) | 7 | 50 | 53 | 186 | **Calc OR 0.41 (0.17 – 0.97); p = 0.044** | calc | - | Univariable |
| Slor et al.; 2011 | Benzodiazepines as medication class  agents: **Diazepam,**  **Lorazepam, Midazolam, Oxazepam** or **Temazepam** | Surgical (hip surgery) | 36 | 430 | 24 | 96 | **Calc OR**  **p < 0.001** | Unadjusted OR-**calc**  Adjusted OR-**ex** | **aOR 0.73 (0.35 – 1.51); p = 0.39** | Multivariate logistic regression analysis |
| Schreiber et al.; 2014 | Benzodiazepines as medication class; agents not specified | Mechanically ventilated ICU patients (ALI) | - | - | - | - | Bivariable regression:  **OR 1.33 (1.01 - 1.75); p = 0.05** | ex | **aOR 1.32 (0.93–1.89);**  **p = 0.12** | Multivariable logistic regression (first-order Markov model)  adjusted for: demographics (age, race, sex), individual covariates (p ≤ 0.2 in bivariable analysis); predefined baseline covariates (e.g. severity of illness score, ICU covariates, other medication classes etc.) |
| Burry et al.; 2017 | Benzodiazepines as medication class; agents not specified  (exposure in the 48h prior to the development of delirium) | Mixed ICU | 210 | 372 | 50 | 148 | **Calc OR: 2.54 (1.71-3.78); p < 0.0001** | Unadjusted OR-**calc**  Adjusted HR-**ex** | **aHR 1.89 (1.16–3.08);**  **p-value not reported** | Multivariable Cox regression analysis  Sensitivity analysis - multivariable analysis assessing each drug class entered independently, controlling for modifiable risk factors (physical restraints)  and non-modifiable risk factors (age, APACHE II score, hypertension, surgical or medical admission, mechanical ventilation, pH < 7.2, alcohol use, tobacco use, prior neurological disease) |
| Schor et al.; 1992 | Benzodiazepines as medication class; agents not specified | General Medicine / Surgery | 32 | 144 | 59 | 147 | **Calc OR 0.43 (0.26 – 0.71); p = 0.001** | Unadjusted OR**-calc**  Adjusted OR**-ex** | **aOR 0.43 (0.23-0.81);**  **p-value not reported** | Multivariable logistic regression analysis adjusted for age and sex |
| Pandharipande et al.; 2006 | **Midazolam**  (ICU-administration) | ICU – mechanically ventilated patients | - | - | - | - | Univariable effect estimate not reported | ex | **aOR 1.7 (0.9 –**  **3.2); p = 0.09** | Multivariable Markov regression model  adjusted for age, sex, visual and hearing deficits, history of dementia, depression, severity of illness (modified APACHE II-score), sepsis, history of neurologic disease, hematocrit, and daily serum glucose concentrations |
| Pandharipande et al.; 2006 | **Lorazepam**  (ICU-administration) | ICU – mechanically ventilated patients | - | - | - | - | Univariable effect estimate not reported | ex | **aOR 1.2 (1.1–1.4);**  **p = 0.003** | Multivariable Markov regression model  adjusted for age, sex, visual and hearing deficits, history of dementia, depression, severity of illness (modified APACHE II-score), sepsis, history of neurologic disease, hematocrit, and daily serum glucose concentrations |
| Gustafson et al.; 1988 | Benzodiazepines as medication class; agents not specified | Orthopedics Surgical (hip surgery) | 12 | 14 | 56 | 97 | **Calc RR 1.48 (1.13 – 1.95); p = 0.005** | cal | **-** | Univariable |
| Marcantonio et al.; 1994 | Benzodiazepines as medication class; agents not specified | Mixed surgical (general, orthopedic surgery and gynecology) | 19 | 31 | 72 | 214 | **OR 3.0 (1.3 - 6.8);**  **p < 0.01** | ex | **-** | Matched analysis |
| **Dose comparisons** | |  |  |  |  |  |  |  |  |  |
| Taipale et al.; 2012 | **Midazolam > 3.0 mg vs < 3.0 mg exposure** | Cardiac ICU | - | - | - | - | Bivariate analysis:  **OR 2.23 (1.06 – 4.70)** | ex | **-** | Bivariable analysis |
| Taipale et al.; 2012 | **Every additional mg of Midazolam** | Cardiac ICU | - | - | - | - | Univariable effect estimate not reported | ex | **aOR 1.08 (1.00 – 1.16); p = 0.04** | Stepwise multivariate logistic regression model adjusted for 15 à priori risk factors and demographics |
| Schreiber et al.; 2014 | **> 5 mg Midazolam-equivalent** in 24 h | Mechanically ventilated ICU patients | - | - | - | - | Bivariable regression:  **OR 1.02 (0.99–1.04); p = 0.13** | ex | **aOR 1.02 (0.99–1.04);**  **p = 0.18** | Multivariable logistic regression (first-order Markov model)  adjusted for demographics (age, race, sex), individual covariates (p ≤ 0.2 in bivariable analysis); predefined baseline covariates (e.g. severity of illness score, ICU covariates, other medication classes) |
| Burry et al.; 2017 | **5 mg Midazolam-equivalents increment**  (exposure in the 48h prior to the development of delirium) | Mixed ICU | - | - | - | - | **HR 1.46 (1.01–2.12); p-value not reported** | **ex** | **aHR 1.08 (1.04–1.12);**  **p-value nor reported** | Multivariable Cox regression model  adjusted for age, APACHE II score on admission, smoking, history of significant alcohol consumption, history of hypertension, presence of pre-existing neurologic condition (e.g., dementia, stroke, neuromuscular disease, seizure disorder), ICU admission type (e.g., surgery), and mechanical ventilation |
| Marcantonio et al.; 1994 | High dose **(> 5mg Diazepam or dose equivalent**) in 24h | Mixed surgical (general, orthopedic surgery and gynecology) | 10 | 15 | 81 | 230 | Univariable effect estimate not reported | ex | **aOR 3.3 (1.0-11.0);**  **p = 0.03** | Matched analysis  Associations of single agents with delirium:  conditional logistic regression model controlled for exposure to all other drugs within that class. |
| Marcantonio et al.; 1994 | Low dose **(< 5mg Diazepam or dose equivalent**) in 24h | Mixed surgical (general, orthopedic surgery and gynecology) | 9 | 16 | 82 | 229 | Univariable effect estimate not reported | ex | **aOR 2.6 (0.8-9.1);**  **p = 0.03** | Matched analysis  Conditional logistic regression model controlled for exposure to all other drugs within that class |
| **Duration of action** | |  |  |  |  |  |  |  |  |  |
| Marcantonio et al.; 1994 | Long-acting benzodiazepines  (**Chlordiazepoxide, Diazepam, Flurazepam**) | Mixed surgical (general, orthopedic surgery and gynecology) | 6 | 9 | 85 | 236 | **Calc OR 3.55 (0.97 – 14.57); p = 0.08** | Unadjusted OR-**calc**  Adjusted OR-**ex** | **aOR 5.4 (1.0-29.2);**  **p = 0.02** | Matched analysis    Conditional logistic regression model controlled for exposure to all other drugs within that class |
| Marcantonio et al.; 1994 | Short-acting benzodiazepines  (**Oxazepam, Lorazepam, Triazolam, Midazolam, Temazepam**) | Mixed surgical (general, orthopedic surgery and gynecology) | 13 | 22 | 78 | 223 | **Calc OR 2.69 (1.10 – 6.56); p = 0.03** | Unadjusted OR-**calc**  Adjusted OR-**ex** | **aOR 2.6 (1.1-6.5);**  **p = 0.02** | Matched analysis    Conditional logistic regression model controlled for exposure to all other drugs within that class |
| **Abbreviations:**  **a**, adjusted; **CI**, confidence interval; **h**, hour(s); **HR**, hazard ratio; **ICU**, intensive care unit; **i.v**., intravenous; **mcg**, microgram; **mg**, milligram; **NRS**, numeric rating scale of pain assessment; **OR**, odds ratio; **RR**, risk ratio;  **Comments:**  **#** high pain scores (NRS ≥ 5) & Hydromorphone dosage > 8 mg versus high pain scores (NRS ≥ 5) & Hydromorphone dosage < 8 mg  **##** high pain scores (NRS < 5) & Hydromorphone dosage > 8 mg versus high pain scores (NRS < 5) & Hydromorphone dosage < 8 mg | | | | | | | | | | |
