# Supplementary material for: Delirium‐associated medication in people at risk: A systematic update review, meta‐analyses, and GRADE‐profiles
Source: Acta Psychiatr Scand. 2022 Oct 11;147(1):16–42. doi: 10.1111/acps.13505 (PMC10092229; doi:10.1111/acps.13505)
Supplement: Supplementary file 2 — Appendix S2 Supporting Information. [file ACPS-147-16-s003.docx]

**META-ANALYSES (MA) & FOREST PLOTS (FP)**

Table of contents

[1. Table of figures 3](#_Toc78993468)

[2. Medication classes – multiple studies 6](#_Toc78993469)

[**I.** **Anticholinergics** 6](#_Toc78993470)

[**II.** **Antidepressants** 9](#_Toc78993471)

[**III.** **Antipsychotics** 10](#_Toc78993472)

[**IV.** **Beta blockers** 14](#_Toc78993473)

[**V.** **Calcium channel blockers** 15](#_Toc78993474)

[**VI.** **H_1_-Antihistamines** 16](#_Toc78993475)

[**VII.** **Polypharmacy** 18](#_Toc78993476)

[**VIII.** **Corticosteroids** 19](#_Toc78993477)

[**IX.** **NSAIDs** 23](#_Toc78993478)

[**X.** **Ketamine** 25](#_Toc78993479)

[**XI.** **Benzodiazepines** 26](#_Toc78993480)

[**XII.** **Opioids** 31](#_Toc78993481)

[3. Medication classes – single studies 38](#_Toc78993482)

[**I.** **ACE-inhibitors** 38](#_Toc78993483)

[**II.** **Antiparkinsonian medication** 38](#_Toc78993484)

[**III.** **Anticoagulants** 38](#_Toc78993485)

[**IV.** **Heparin** 39](#_Toc78993486)

[**V.** **Insulin** 39](#_Toc78993487)

[**VI.** **Diuretics** 39](#_Toc78993488)

[**VII.** **Antibiotics** 40](#_Toc78993489)

[**VIII.** **H_2_-Antihistamines** 40](#_Toc78993490)

[**IX.** **Digoxin** 41](#_Toc78993491)

[**X.** **Paracetamol = Acetaminophen** 42](#_Toc78993492)

[**XI.** **Propofol** 43](#_Toc78993493)

## Table of figures

[figure 1: MA of one RCT and three observational studies on anticholinergics - adjusted ORs 6](#_Toc67418826)

[figure 2: MA of one RCT and four observational studies on anticholinergics - unadjusted ORs 7](#_Toc67418827)

[figure 3: Forest plot of one observational study on anticholinergics - adjusted HR 7](#_Toc67418828)

[figure 4: Forest plot of one observational study on anticholinergics – unadjusted HR 8](#_Toc67418829)

[figure 5: Forest plot of one RCT on Atropine 8](#_Toc67418830)

[figure 6: Forest plot of one RCT on Penehyclidine hydrochloride 8](#_Toc67418831)

[figure 7: Forest plot of one observational study on antidepressants - adjusted OR 9](#_Toc67418832)

[figure 8: MA of four observational studies on antidepressants - unadjusted ORs 9](#_Toc67418833)

[figure 9: MA of six RCTs and two observational studies on antipsychotics - adjusted ORs 11](#_Toc67418834)

[figure 10: MA of six RCTs and five observational studies on antipsychotics - unadjusted ORs 12](#_Toc67418835)

[figure 11: MA of five RCTs on Haloperidol 13](#_Toc67418836)

[figure 12: Forest plot of one RCT on Olanzapine 13](#_Toc67418837)

[figure 13: Forest plot of one observational study on beta blockers – adjusted HR 14](#_Toc67418838)

[figure 14: Forest plot of one observational study on beta blockers – unadjusted HR 14](#_Toc67418839)

[figure 15: MA of two observational studies on beta blockers - unadjusted ORs 15](#_Toc67418840)

[figure 16: Forest plot of one observational study on Nifedipine – adjusted OR 15](#_Toc67418841)

[figure 17: MA of two observational studies on calcium channel blockers – unadjusted ORs 16](#_Toc67418842)

[figure 18: MA of two observational studies on H_1_-Antihistamines – adjusted ORs 16](#_Toc67418843)

[figure 19: MA of three observational studies on H_1_-Antihistamines – unadjusted ORs 17](#_Toc67418844)

[figure 20: MA of two observational studies on Diphenhydramine – adjusted ORs 17](#_Toc67418845)

[figure 21: MA of two observational studies on Diphenhydramine – unadjusted ORs 18](#_Toc67418846)

[figure 22: MA of two observational studies on polypharmacy – adjusted ORs 18](#_Toc67418847)

[figure 23: MA of three observational studies on polypharmacy – unadjusted ORs 19](#_Toc67418848)

[figure 24: MA of two RCTs and four observational studies on corticosteroids – adjusted ORs 20](#_Toc67418849)

[figure 25: MA of two RCTs and six observational studies on corticosteroids – unadjusted ORs 21](#_Toc67418850)

[figure 26: MA of five observational studies on corticosteroids – unadjusted RRs and HR 22](#_Toc67418851)

[figure 27: Forest plot of one observational study on corticosteroids – adjusted HR 22](#_Toc67418852)

[figure 28: Forest plots of two observational studies on prednisone-equivalent increments – adjusted ORs 23](#_Toc67418853)

[figure 29: MA of two observational studies on NSAIDs – adjusted ORs 23](#_Toc67418854)

[figure 30: MA of two observational studies on NSAIDs – unadjusted ORs 24](#_Toc67418855)

[figure 31: Forest plot of one observational study on acetylsalicylic acid – adjusted OR 24](#_Toc67418856)

[figure 32: Forest plot of one observational study on Naproxen – adjusted OR 25](#_Toc67418857)

[figure 33: MA of two RCTs on Ketamine 25](#_Toc67418858)

[figure 34: Forest plots of one RCT on Ketamine dosages – adjusted ORs 26](#_Toc67418859)

[figure 35: MA of four observational studies on benzodiazepines – adjusted ORs 26](#_Toc67418860)

[figure 36: Forest plot of one observational study on benzodiazepines – adjusted HR 27](#_Toc67418861)

[figure 37: MA of eight observational studies on benzodiazepines – unadjusted ORs 27](#_Toc67418862)

[figure 38: MA of nine observational studies on benzodiazepines – unadjusted ORs 28](#_Toc67418863)

[figure 39: MA of two observational studies on Midazolam – adjusted and unadjusted ORs 28](#_Toc67418864)

[figure 40: Forest plot of one observational study on Lorazepam – adjusted OR 29](#_Toc67418865)

[figure 41: Forest plots of two observational studies on dosages of Midazolam-equivalents – adjusted ORs 29](#_Toc67418866)

[figure 42: Forest plot of one observational study on Midazolam dose increment – adjusted HR 30](#_Toc67418867)

[figure 43: Forest plots of one observational study on dosages of Diazepam-equivalents – adjusted ORs 30](#_Toc67418868)

[figure 44: Forest plots of one observational study on benzodiazepine duration of action – adjusted ORs 31](#_Toc67418869)

[figure 45: MA of three observational studies on opioids – adjusted ORs 31](#_Toc67418870)

[figure 46: MA of nine observational studies on opioids – unadjusted ORs 32](#_Toc67418871)

[figure 47: MA of eight observational studies on opioids – unadjusted ORs 32](#_Toc67418872)

[figure 48: MA of two observational studies on Morphine – adjusted ORs 33](#_Toc67418873)

[figure 49: MA of two observational studies on Fentanyl – adjusted ORs 33](#_Toc67418874)

[figure 50: Forest plot of one observational study on Pethidine – adjusted RR 34](#_Toc67418875)

[figure 51: MA of two observational studies on Pethidine – unadjusted ORs 34](#_Toc67418876)

[figure 52: Forest plot of one observational study on Oxycodone – adjusted OR 35](#_Toc67418877)

[figure 53: Forest plot of one observational study on Codeine – adjusted OR 35](#_Toc67418878)

[figure 54: Forest plots of one observational study on opioid dosage – adjusted ORs 36](#_Toc67418879)

[figure 55: Forest plots of one observational study on opioid dosage (inverse correlation) – adjusted RRs 36](#_Toc67418880)

[figure 56: Forest plot of one observational study on dose increment – adjusted RR 37](#_Toc67418881)

[figure 57: Forest plot of one observational study on ACE-inhibitors – unadjusted OR 38](#_Toc67418882)

[figure 58: Forest plot of one observational study on antiparkinsonian medication – unadjusted OR 38](#_Toc67418883)

[figure 59: Forest plot of one observational study on anticoagulants – unadjusted OR 38](#_Toc67418884)

[figure 60: Forest plot of one observational study on heparin – unadjusted OR 39](#_Toc67418885)

[figure 61: Forest plot of one observational study on insulin – unadjusted OR 39](#_Toc67418886)

[figure 62: Forest plot of one observational study on diuretics – unadjusted OR 39](#_Toc67418887)

[figure 63: Forest plot of one observational study on antibiotics – unadjusted OR 40](#_Toc67418888)

[figure 64: Forest plot of one observational study on H_2_-antihistamines – adjusted OR 40](#_Toc67418889)

[figure 65: Forest plot of one observational study on H_2_-antihistamines – unadjusted OR 41](#_Toc67418890)

[figure 66: Forest plot of one observational study on digoxin – adjusted OR 41](#_Toc67418891)

[figure 67: Forest plot of one observational study on digoxin – unadjusted OR 41](#_Toc67418892)

[figure 68: Forest plot of one observational study on paracetamol – adjusted OR 42](#_Toc67418893)

[figure 69: Forest plot of one observational study on paracetamol – unadjusted OR 42](#_Toc67418894)

[figure 70: Forest plot of one observational study on propofol – adjusted OR 43](#_Toc67418895)

[figure 71: Forest plots of one observational study on dose increment of propofol – adjusted & unadjusted HR 43](#_Toc67418896)

## Medication classes – multiple studies

### **Anticholinergics**

1. **RCT**

figure 1: Forest plot of one RCT on anticholinergics


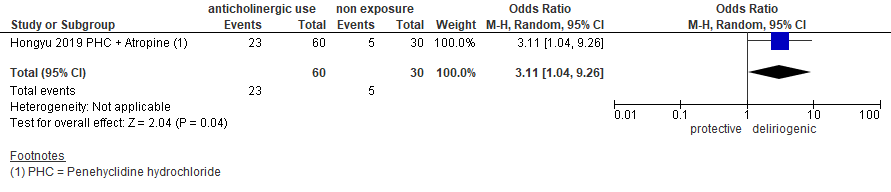


1. **Adjusted estimates (n = 4)**

figure 2: MA of one RCT and three observational studies on anticholinergics - adjusted ORs

**
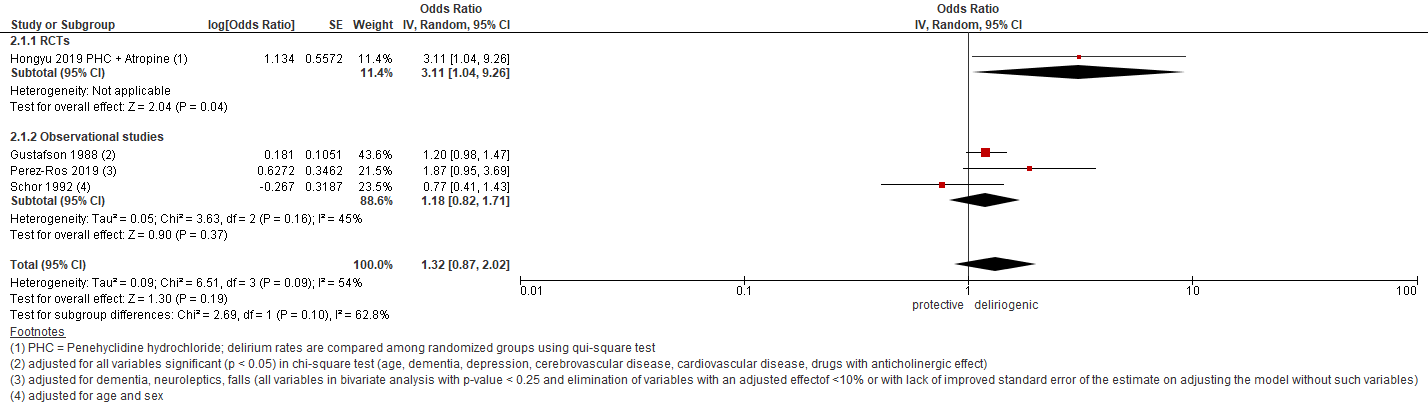
**

1. **Unadjusted estimates (n = 5)**

figure 3: MA of one RCT and four observational studies on anticholinergics - unadjusted ORs

**
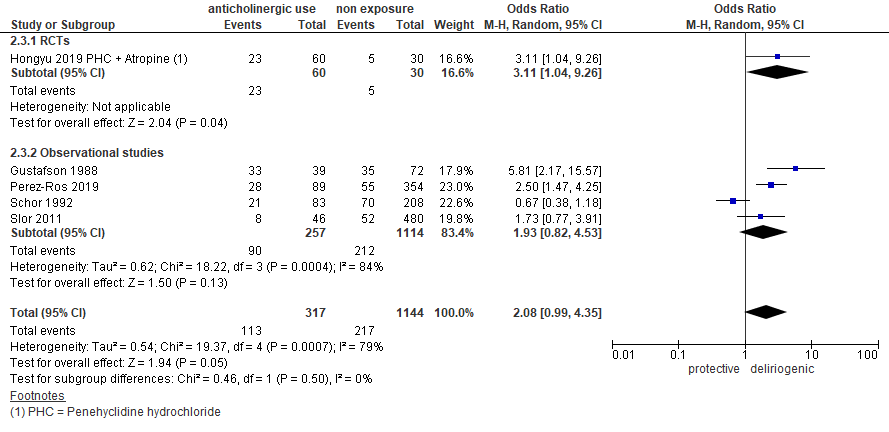
**

1. **Adjusted HR (n = 1)**

figure 4: Forest plot of one observational study on anticholinergics - adjusted HR


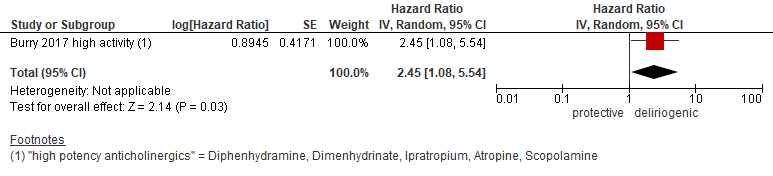


adjusted for age, APACHE II score on admission, smoking, history of significant alcohol consumption, history of hypertension, presence of pre-existing neurologic condition (e.g., dementia, stroke, neuromuscular disease, seizure disorder), ICU admission type (e.g., surgery), and mechanical ventilation

1. **Unadjusted HR (n = 1)**

figure 5: Forest plot of one observational study on anticholinergics – unadjusted HR

**
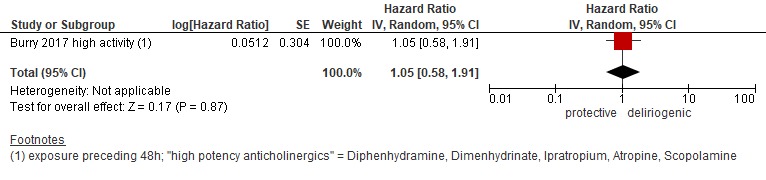
**

1. **Atropine (n = 1)**

figure 6: Forest plot of one RCT on Atropine

**
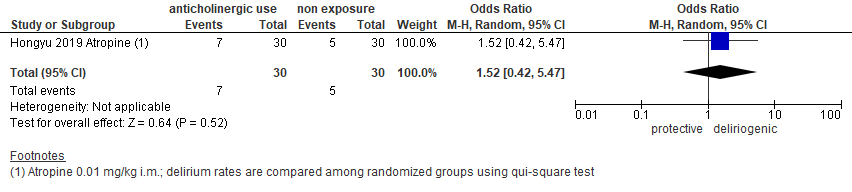
**

1. **Penehyclidine hydrochloride (n = 1)**

figure 7: Forest plot of one RCT on Penehyclidine hydrochloride


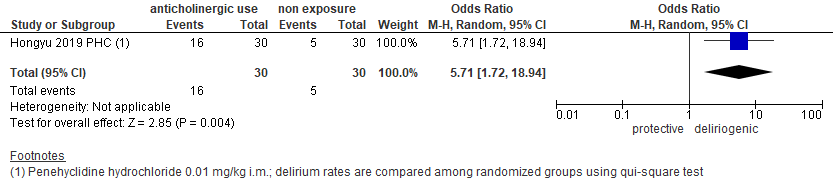


### **Antidepressants**

1. **Adjusted estimate (n = 1)**

figure 8: Forest plot of one observational study on antidepressants - adjusted OR


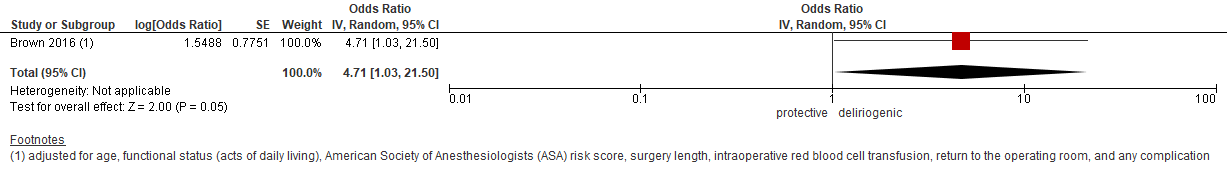


1. **Unadjusted estimates (n = 4)**

figure 9: MA of four observational studies on antidepressants - unadjusted ORs


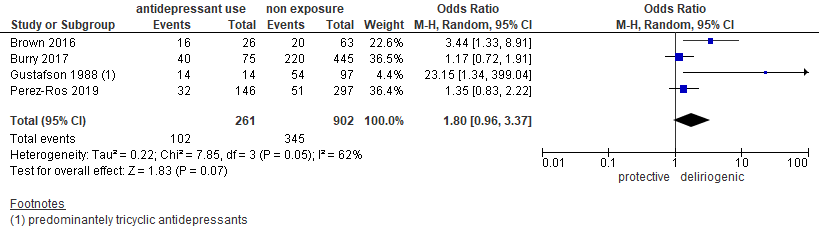


### **Antipsychotics**

1. **RCTs on antipsychotics as medication class**

figure 10: MA of 6 RCTs on antipsychotics


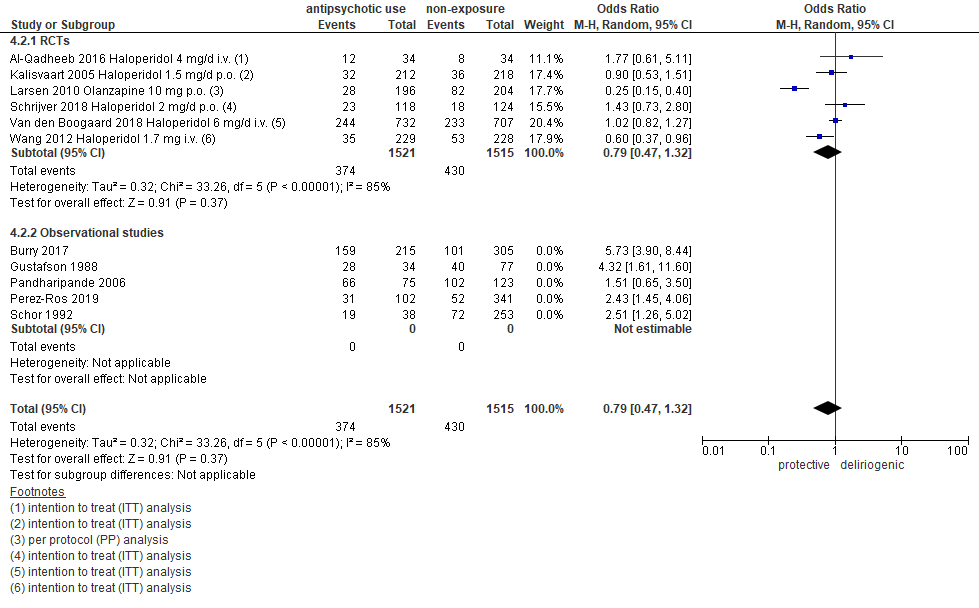


1. **RCTs & observational studies - adjusted estimates (n = 8)**

figure 11: MA of six RCTs and two observational studies on antipsychotics - adjusted ORs


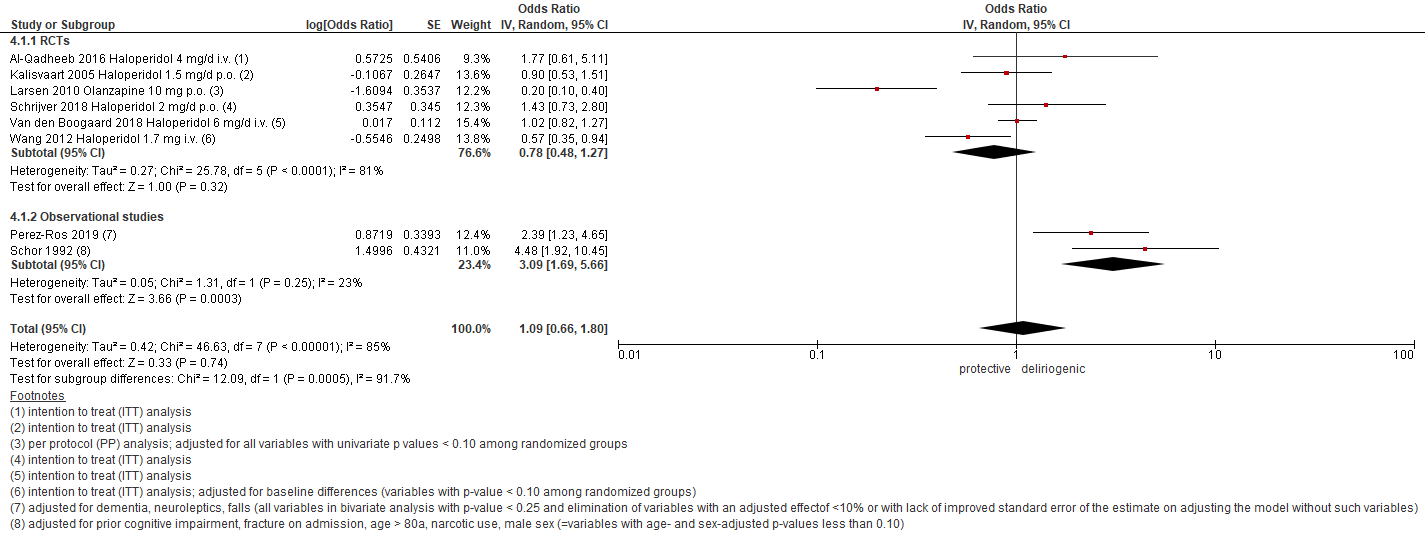


1. **RCTs & observational studies - unadjusted estimates (n = 11)**

figure 12: MA of six RCTs and five observational studies on antipsychotics - unadjusted ORs


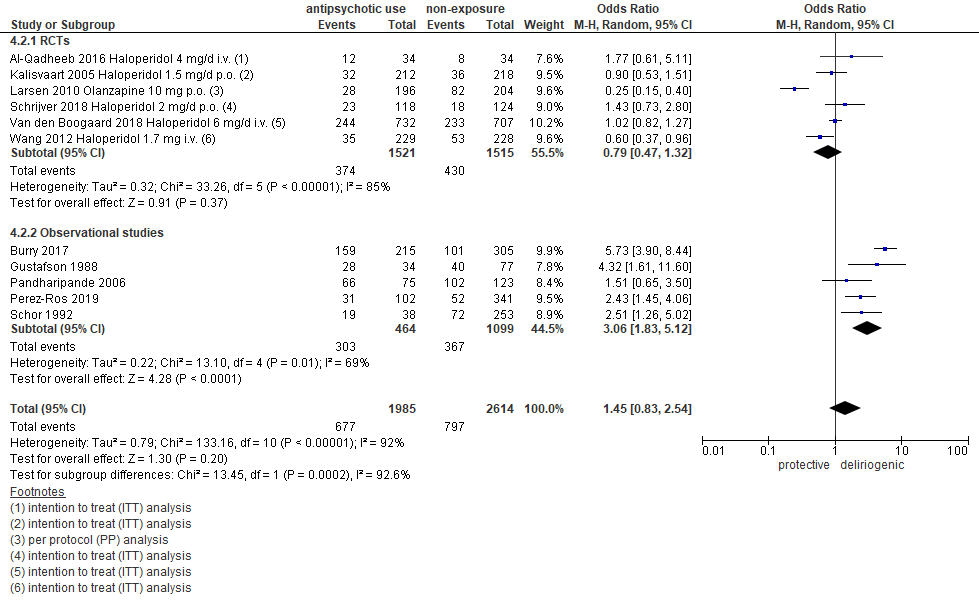


1. **Haloperidol (n = 5)**

figure 13: MA of five RCTs on Haloperidol


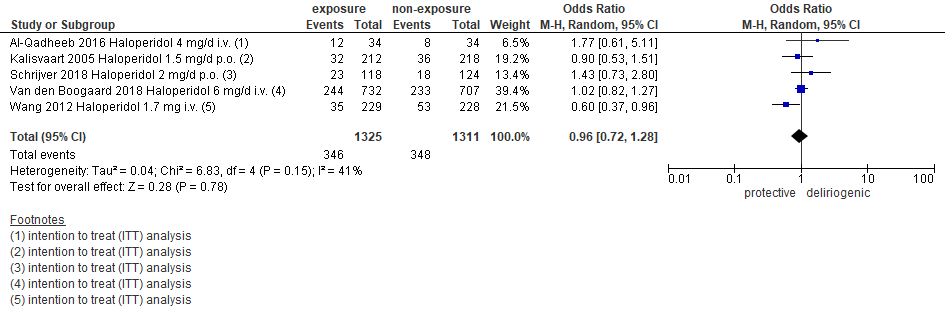


1. **Olanzapine (n = 1)**

figure 14: Forest plot of one RCT on Olanzapine


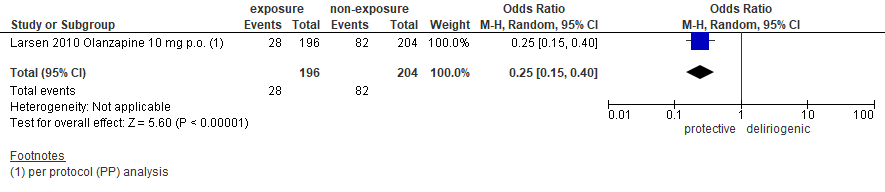


### **Beta blockers**

1. **Adjusted HR (n = 1)**

figure 15: Forest plot of one observational study on beta blockers – adjusted HR


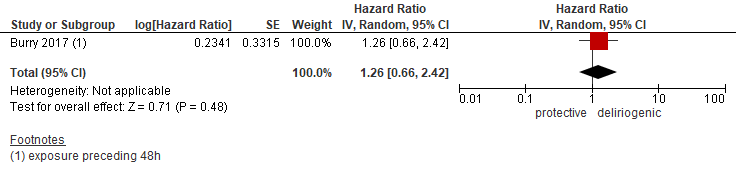


adjusted for age, APACHE II score on admission, smoking, history of significant alcohol consumption, history of hypertension, presence of pre-existing neurologic condition (e.g., dementia, stroke, neuromuscular disease, seizure disorder), ICU admission type (e.g., surgery), and mechanical ventilation

1. **Unadjusted HR (n = 1)**

figure 16: Forest plot of one observational study on beta blockers – unadjusted HR


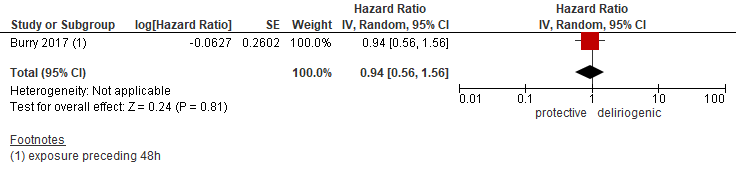


1. **Unadjusted estimates (n = 2)**

figure 17: MA of two observational studies on beta blockers - unadjusted ORs


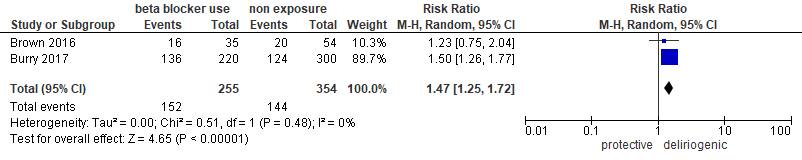


### **Calcium channel blockers**

1. **Nifedipine – adjusted estimate (n = 1)**

figure 18: Forest plot of one observational study on Nifedipine – adjusted OR


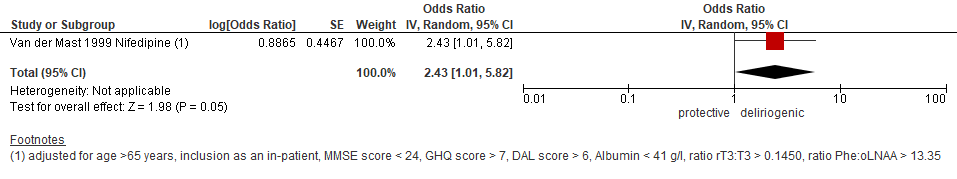


1. **All calcium channel blockers - unadjusted estimates (n = 2)**

figure 19: MA of two observational studies on calcium channel blockers – unadjusted ORs


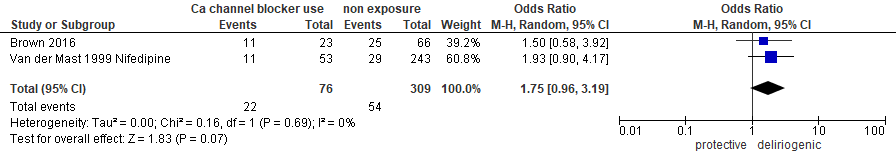


### **H_1_-Antihistamines**

1. **All H_1_-Antihistamines – adjusted estimates (n = 2)**

figure 20: MA of two observational studies on H_1_-Antihistamines – adjusted ORs


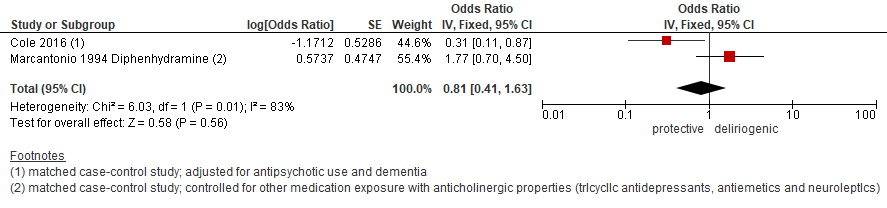


1. **All H_1_-Antihistamines – unadjusted estimates (n = 3)**

figure 21: MA of three observational studies on H_1_-Antihistamines – unadjusted ORs


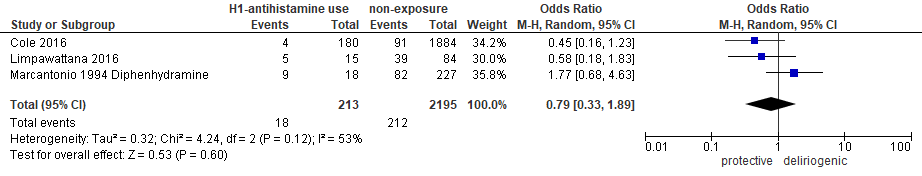


1. **Diphenhydramine – adjusted estimates (n = 2)**

figure 22: MA of two observational studies on Diphenhydramine – adjusted ORs


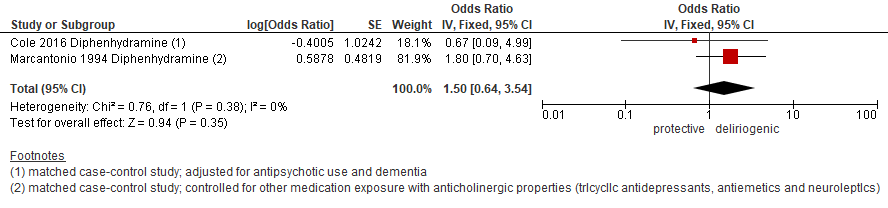


1. **Diphenhydramine – unadjusted estimates (n = 2)**

figure 23: MA of two observational studies on Diphenhydramine – unadjusted ORs


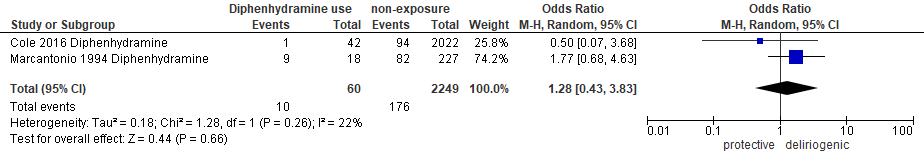


### **Polypharmacy**

1. **Adjusted estimates (n = 2)**

figure 24: MA of two observational studies on polypharmacy – adjusted ORs


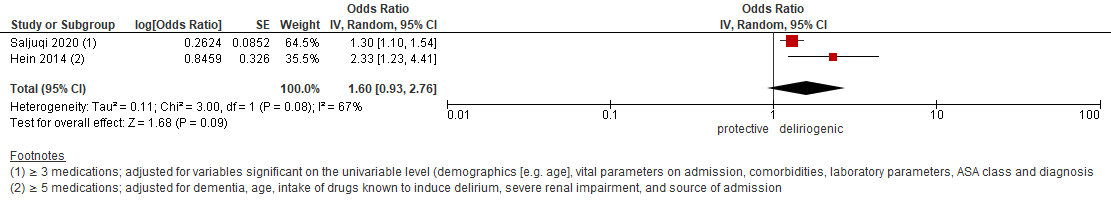


1. **Unadjusted estimates (n = 3)**

figure 25: MA of three observational studies on polypharmacy – unadjusted ORs


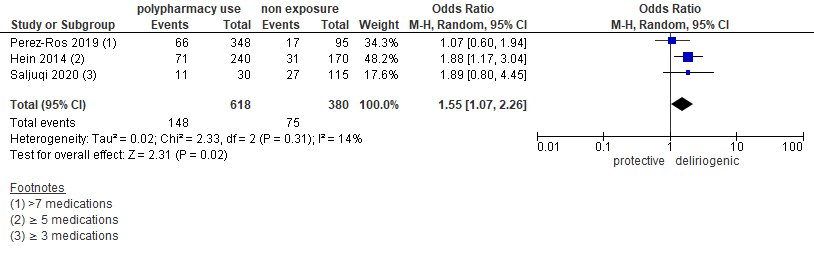


### **Corticosteroids**

1. **RCTs on corticosteroids as medication class (n = 2)**

figure 26: MA of two RCTs on corticosteroids


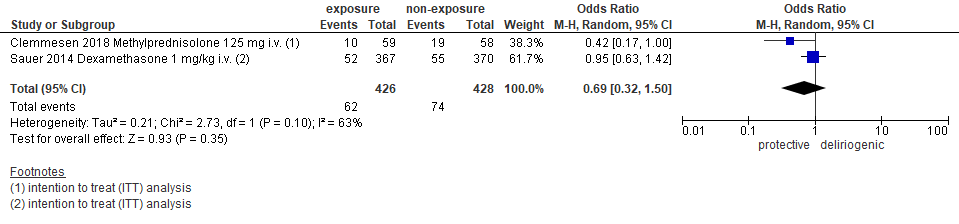


1. **RCTs and observational studies pooled - adjusted estimates (n = 6)**

figure 27: MA of two RCTs and four observational studies on corticosteroids – adjusted ORs


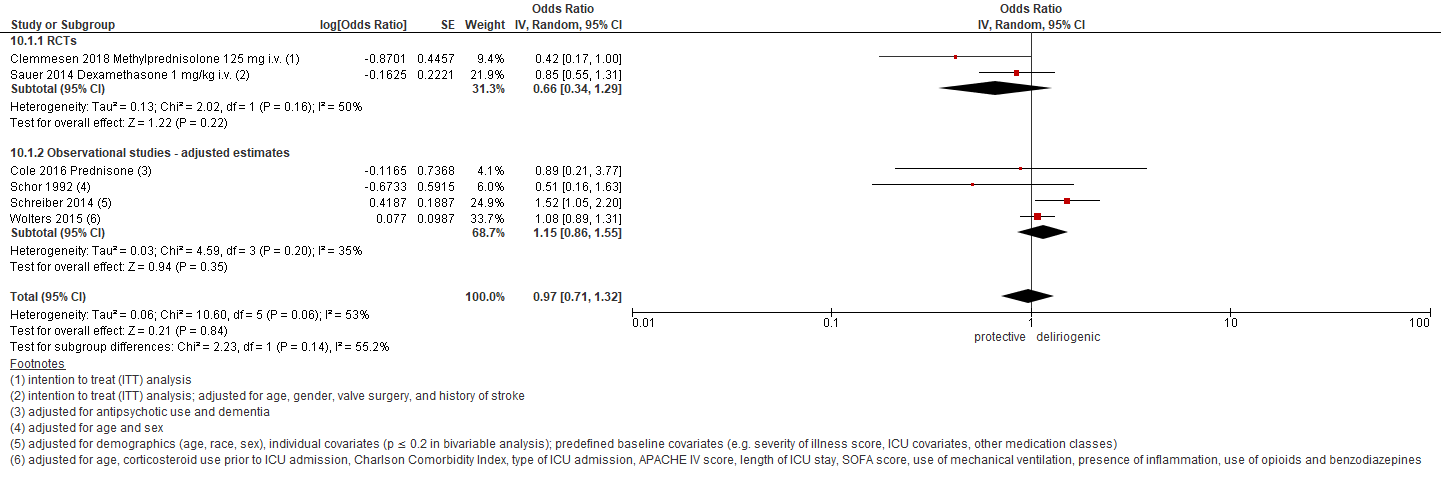


1. **RCTs and observational studies pooled - unadjusted estimates (n = 8)**

figure 28: MA of two RCTs and six observational studies on corticosteroids – unadjusted ORs


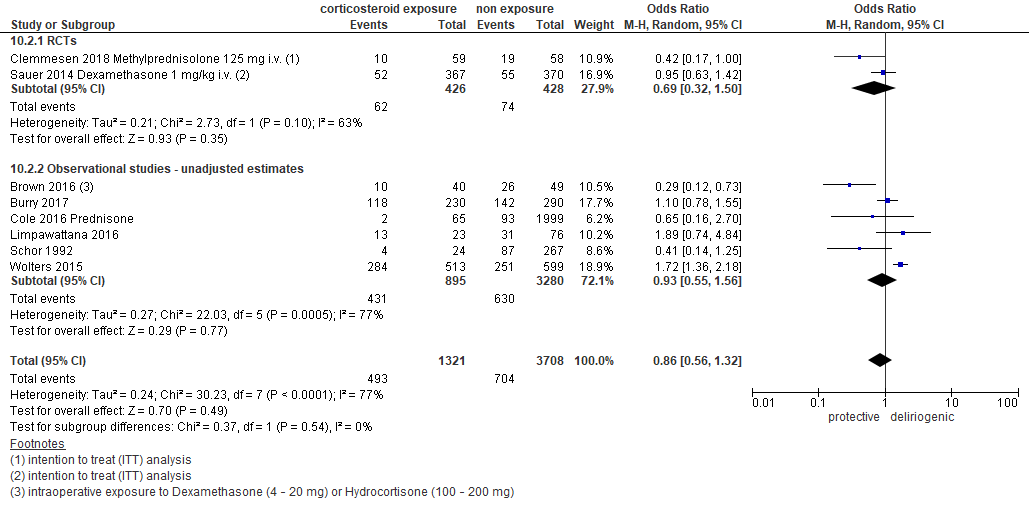


1. **Observational studies - unadjusted estimates – RRs & HR (n = 5)**

figure 29: MA of five observational studies on corticosteroids – unadjusted RRs and HR


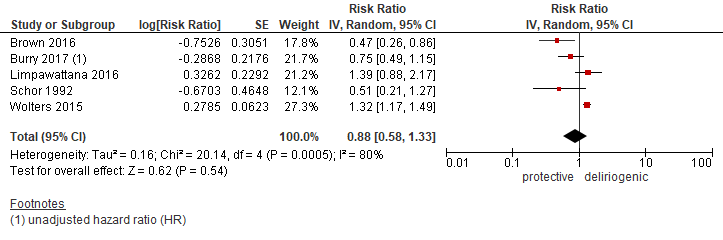


1. **Adjusted HR (n = 1)**

figure 30: Forest plot of one observational study on corticosteroids – adjusted HR


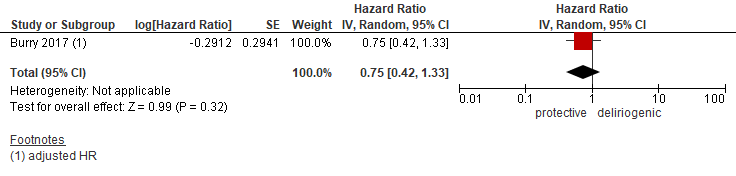


adjusted for age, APACHE II score on admission, smoking, history of significant alcohol consumption, history of hypertension, presence of pre-existing neurologic condition (e.g., dementia, stroke, neuromuscular disease, seizure disorder), ICU admission type (e.g., surgery), and mechanical ventilation

1. **Prednisone-equivalent dose increments (n = 2)**

figure 31: Forest plots of two observational studies on prednisone-equivalent increments – adjusted ORs


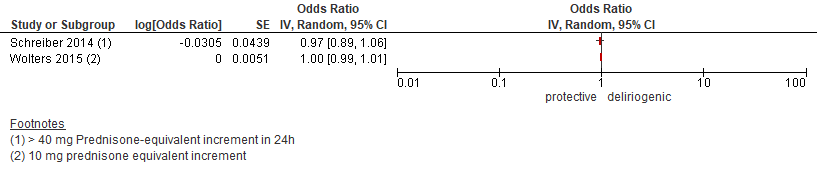


1. adjusted for demographics (age, race, sex), individual covariates (p ≤ 0.2 in bivariable analysis), predefined baseline covariates (e.g. severity of illness score, ICU covariates, other medication classes)
2. adjusted for age, corticosteroid use prior to ICU admission, Charlson Comorbidity Index, type of ICU admission, APACHE IV score, length of ICU stay, SOFA score, use of mechanical ventilation, presence of inflammation, use of opioids and benzodiazepines

### **NSAIDs**

1. **Adjusted estimates (n = 2)**

figure 32: MA of two observational studies on NSAIDs – adjusted ORs


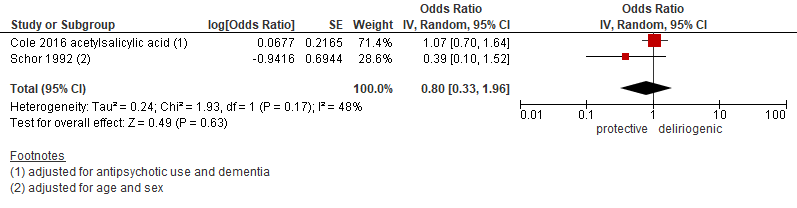


1. **Unadjusted estimates (n = 2)**

figure 33: MA of two observational studies on NSAIDs – unadjusted ORs


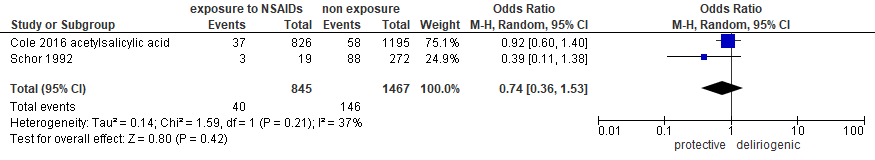


1. **ASA – adjusted estimate (n = 1)**

figure 34: Forest plot of one observational study on acetylsalicylic acid – adjusted OR


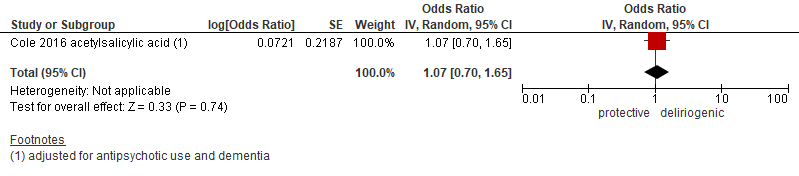


1. **Naproxen – adjusted estimate (n = 1)**

figure 35: Forest plot of one observational study on Naproxen – adjusted OR


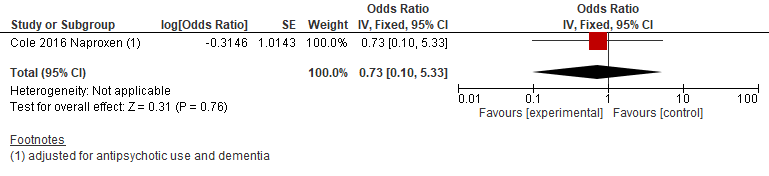


### **Ketamine**

1. **RCTs (n = 2)**

figure 36: MA of two RCTs on Ketamine


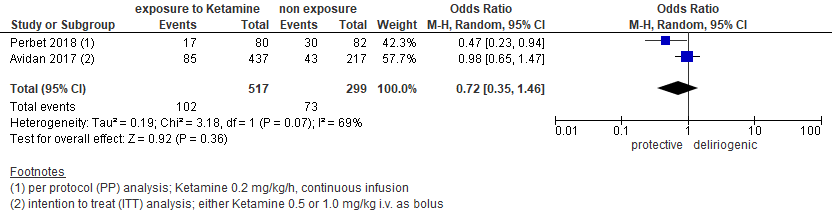


1. **Ketamine dosages (n = 1)**

figure 37: Forest plots of one RCT on Ketamine dosages – adjusted ORs


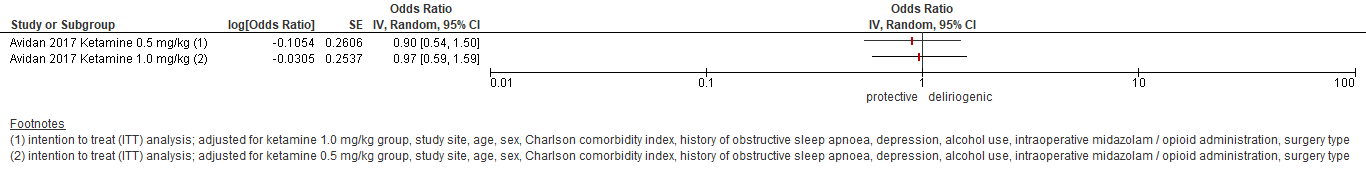


### **Benzodiazepines**

1. **Adjusted estimates (n = 4)**

figure 38: MA of four observational studies on benzodiazepines – adjusted ORs


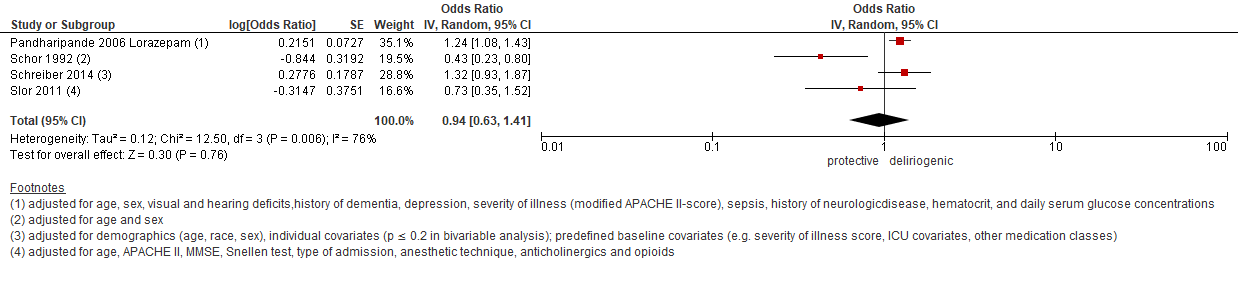


1. **Adjusted HR (n = 1)**

figure 39: Forest plot of one observational study on benzodiazepines – adjusted HR


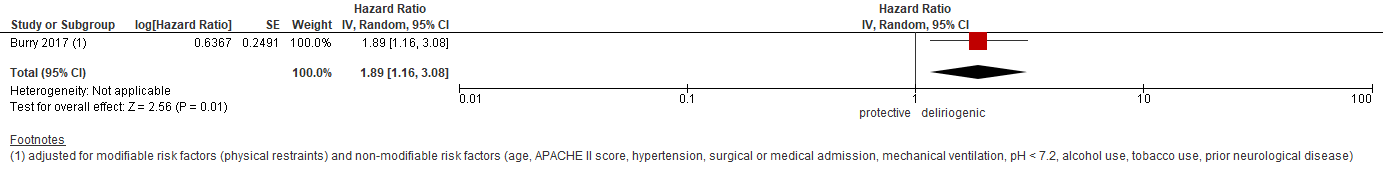


1. **Unadjusted estimates – Mantel Haenszel analysis (n = 8)**

figure 40: MA of eight observational studies on benzodiazepines – unadjusted ORs


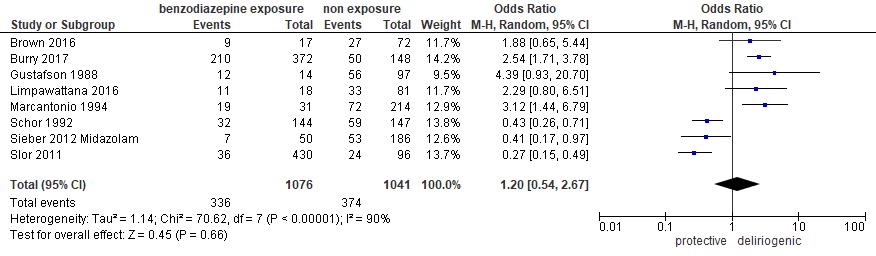


1. **Unadjusted estimates – generic inverse variance analysis (n = 9)**

figure 41: MA of nine observational studies on benzodiazepines – unadjusted ORs


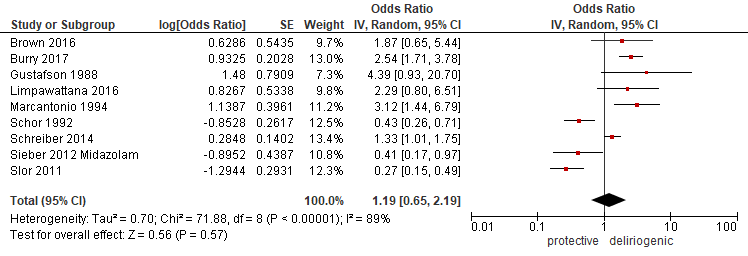


1. **Midazolam (n =2)**

figure 42: MA of two observational studies on Midazolam – adjusted and unadjusted ORs


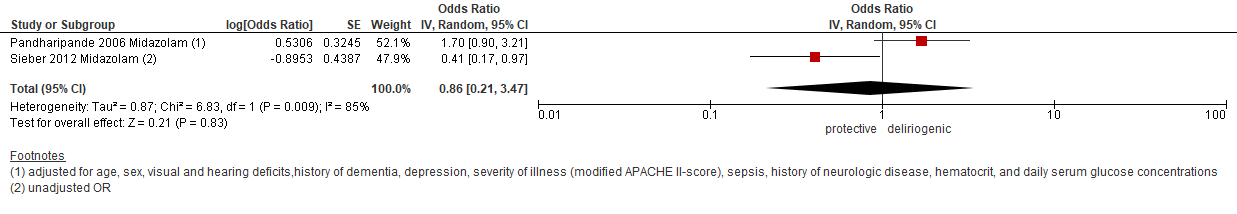


1. **Lorazepam – adjusted estimate (n = 1)**

figure 43: Forest plot of one observational study on Lorazepam – adjusted OR


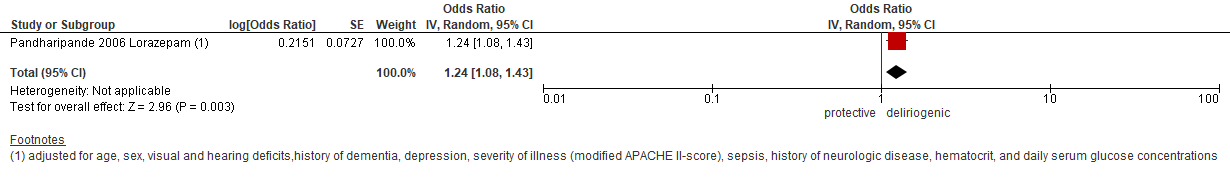


1. **Dosages**

- **Midazolam-equivalents – adjusted estimates (n = 2)**

figure 44: Forest plots of two observational studies on dosages of Midazolam-equivalents – adjusted ORs


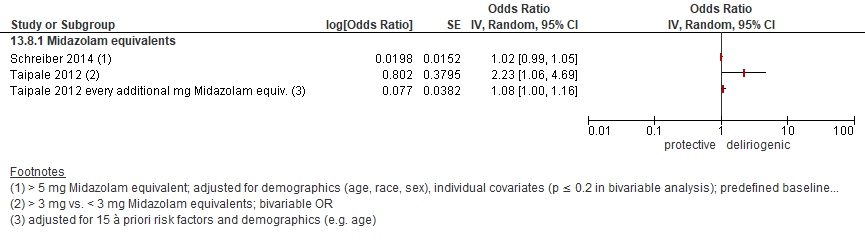


- **Midazolam dose increment – adjusted estimate (n = 1)**

figure 45: Forest plot of one observational study on Midazolam dose increment – adjusted HR


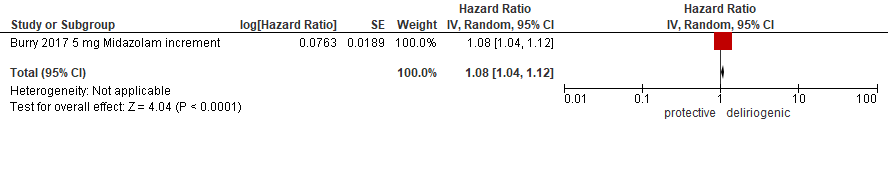


adjusted for age, APACHE II score on admission, smoking, history of significant alcohol consumption, history of hypertension, presence of pre-existing neurologic condition (e.g., dementia, stroke, neuromuscular disease, seizure disorder), ICU admission type (e.g. surgery), and mechanical ventilation

- **Diazepam-equivalents – adjusted estimates (n = 1)**

figure 46: Forest plots of one observational study on dosages of Diazepam-equivalents – adjusted ORs


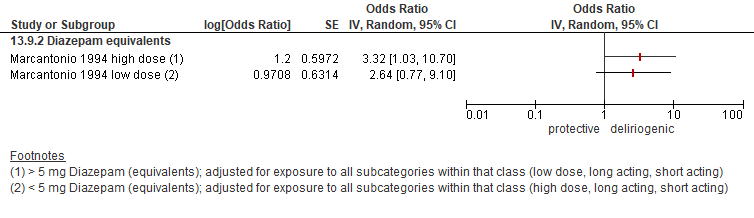


1. **Duration of action – adjusted estimates (n = 1)**

figure 47: Forest plots of one observational study on benzodiazepine duration of action – adjusted ORs


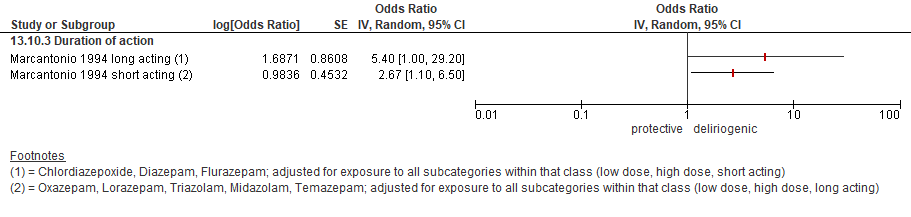


### **Opioids**

1. **Adjusted estimates (n = 3)**

figure 48: MA of three observational studies on opioids – adjusted ORs


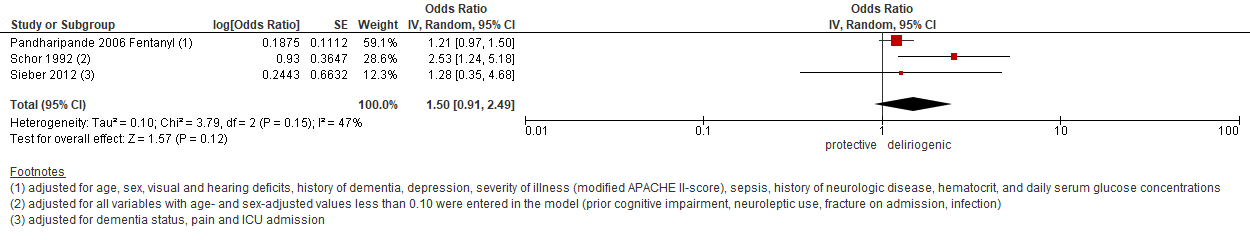


1. **Unadjusted estimates – generic inverse variance analysis (n = 9)**

figure 49: MA of nine observational studies on opioids – unadjusted ORs


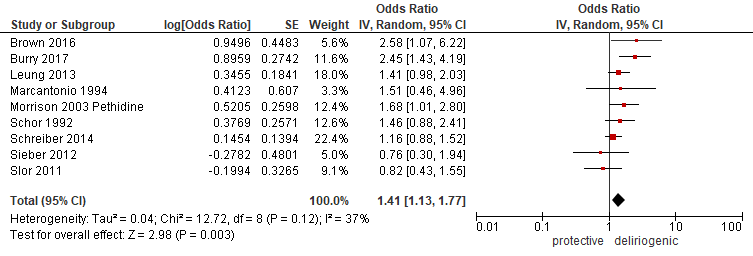


1. **Unadjusted estimates – Mantel Haenszel analysis (n = 8)**

figure 50: MA of eight observational studies on opioids – unadjusted ORs


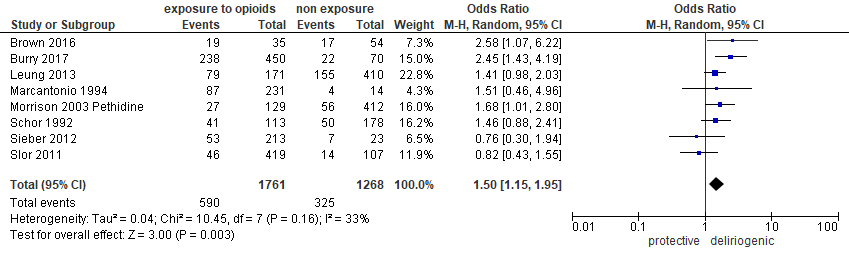


1. **Morphine – adjusted estimates (n = 2)**

figure 51: MA of two observational studies on Morphine – adjusted ORs


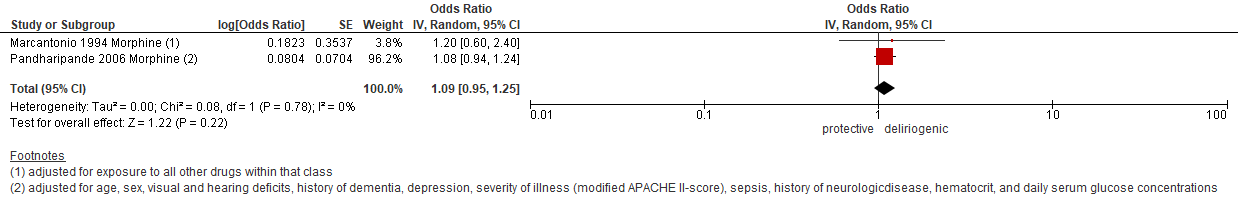


1. **Fentanyl – adjusted estimates (n = 2)**

figure 52: MA of two observational studies on Fentanyl – adjusted ORs


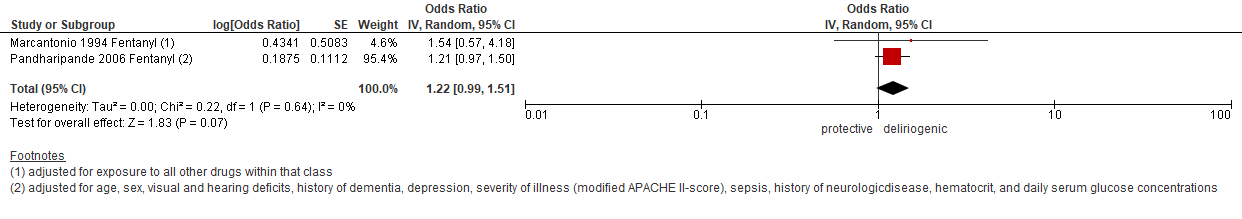


1. **Pethidine = Meperidine – adjusted estimate (n = 1)**

figure 53: Forest plot of one observational study on Pethidine – adjusted RR


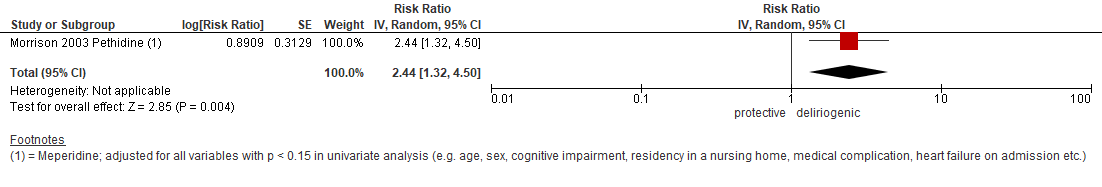


1. **Pethidine = Meperidine – unadjusted estimates (n = 2)**

figure 54: MA of two observational studies on Pethidine – unadjusted ORs


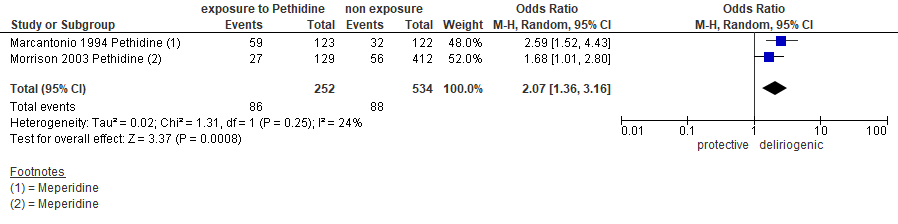


1. **Oxycodone – adjusted estimate (n = 1)**

figure 55: Forest plot of one observational study on Oxycodone – adjusted OR


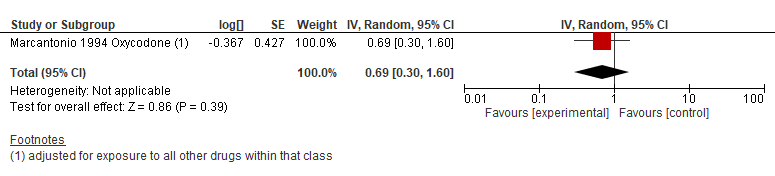


1. **Codeine – adjusted estimate (n = 1)**

figure 56: Forest plot of one observational study on Codeine – adjusted OR


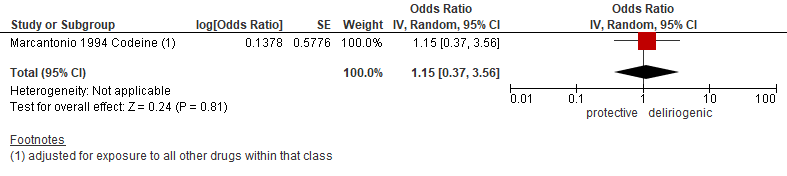


1. **Dosage: high vs. low dose – adjusted estimates (n = 1)**

figure 57: Forest plots of one observational study on opioid dosage – adjusted ORs


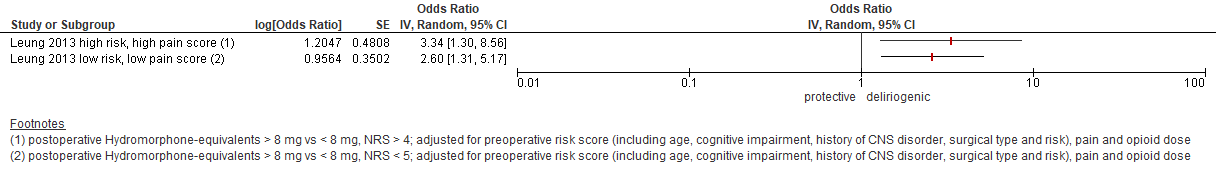


1. **Dosage: inverse correlation – adjusted estimates (n = 1)**

figure 58: Forest plots of one observational study on opioid dosage (inverse correlation) – adjusted RRs


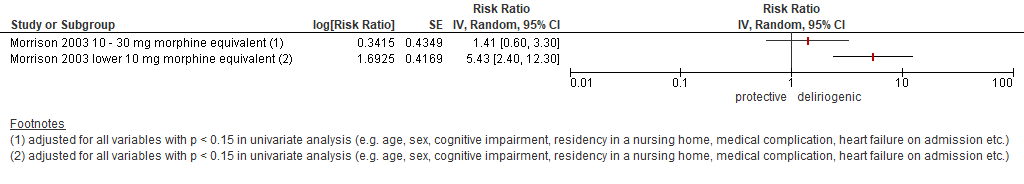


1. **Dose increment – adjusted estimate (n = 1)**

figure 59: Forest plot of one observational study on dose increment – adjusted RR


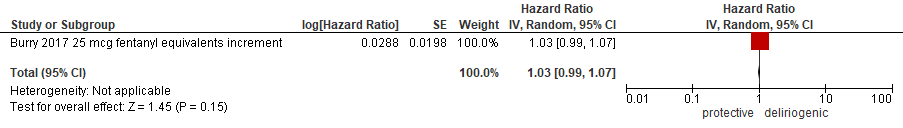


adjusted for age, APACHE II score on admission, smoking, history of significant alcohol consumption, history of hypertension, presence of pre-existing neurologic condition (e.g. dementia, stroke, neuromuscular disease, seizure disorder), ICU admission type (e.g. surgery), and mechanical ventilation

## Medication classes – single studies

### **ACE-inhibitors**

figure 60: Forest plot of one observational study on ACE-inhibitors – unadjusted OR


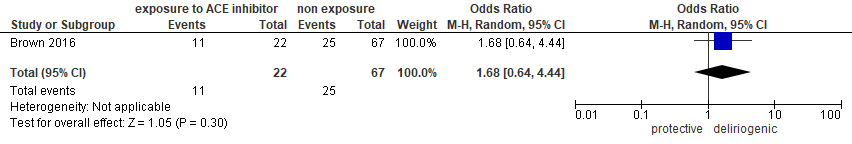


### **Antiparkinsonian medication**

figure 61: Forest plot of one observational study on antiparkinsonian medication – unadjusted OR


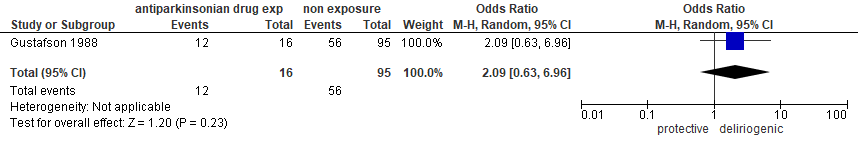


### **Anticoagulants**

figure 62: Forest plot of one observational study on anticoagulants – unadjusted OR


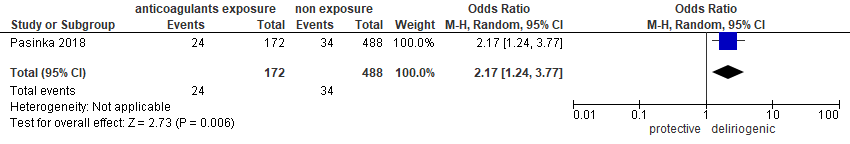


### **Heparin**

figure 63: Forest plot of one observational study on heparin – unadjusted OR


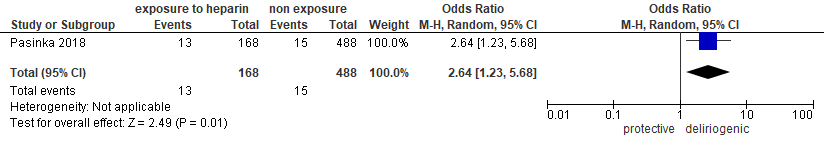


### **Insulin**

figure 64: Forest plot of one observational study on insulin – unadjusted OR


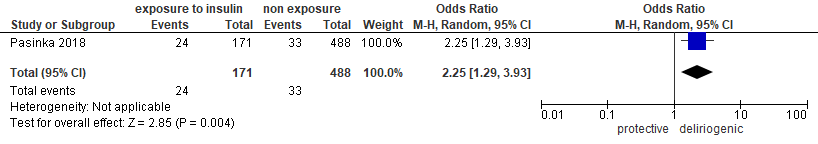


### **Diuretics**

figure 65: Forest plot of one observational study on diuretics – unadjusted OR


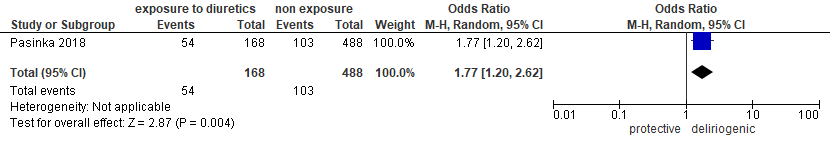


### **Antibiotics**

figure 66: Forest plot of one observational study on antibiotics – unadjusted OR


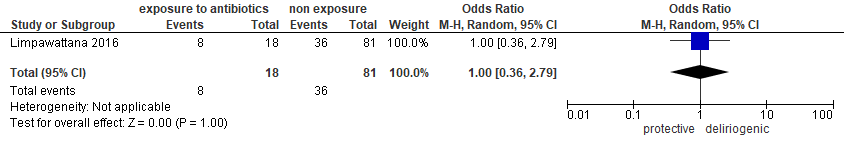


### **H_2_-Antihistamines**

1. **Adjusted estimate**

figure 67: Forest plot of one observational study on H_2_-antihistamines – adjusted OR


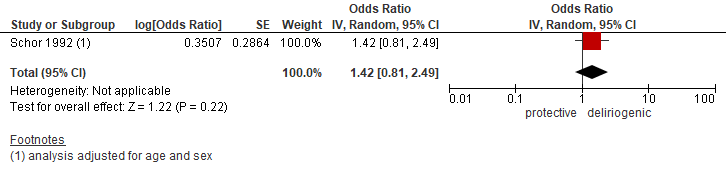


1. **Unadjusted estimate**

figure 68: Forest plot of one observational study on H_2_-antihistamines – unadjusted OR


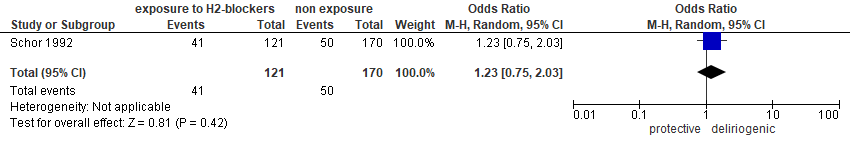


### **Digoxin**

1. **Adjusted estimate**

figure 69: Forest plot of one observational study on digoxin – adjusted OR


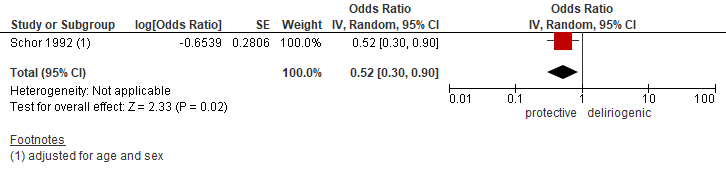


1. **Unadjusted estimate**

figure 70: Forest plot of one observational study on digoxin – unadjusted OR


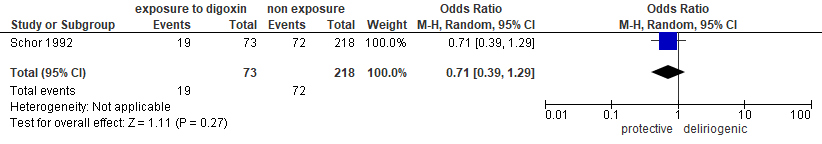


### **Paracetamol = Acetaminophen**

1. **Adjusted estimate**

figure 71: Forest plot of one observational study on paracetamol – adjusted OR


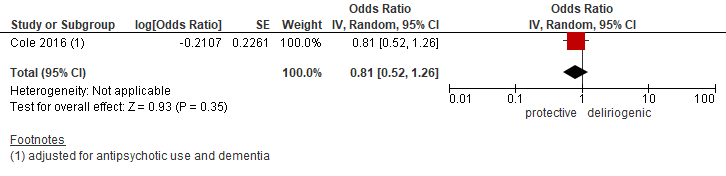


1. **Unadjusted estimate**

figure 72: Forest plot of one observational study on paracetamol – unadjusted OR


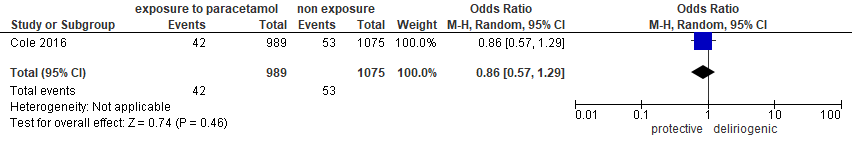


### **Propofol**

1. **Adjusted estimate**

figure 73: Forest plot of one observational study on propofol – adjusted OR


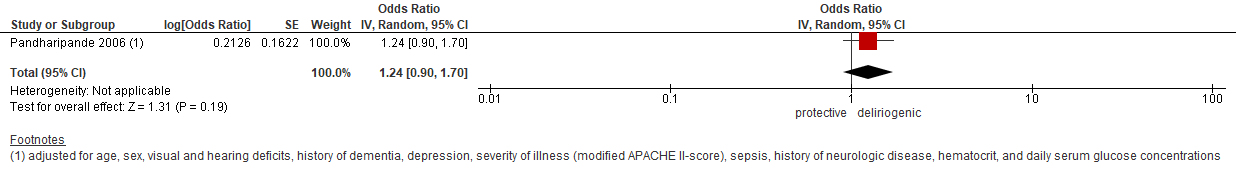


1. **Dose increment (adjusted & unadjusted estimate)**

figure 74: Forest plots of one observational study on dose increment of propofol – adjusted & unadjusted HR


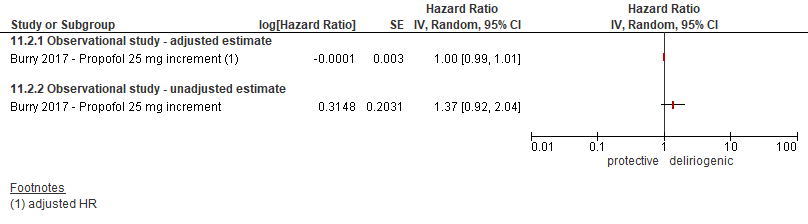


1. adjusted for age, APACHE II score on admission, smoking, history of significant alcohol consumption, history of hypertension, presence of pre-existing neurologic condition (e.g., dementia, stroke, neuromuscular disease, seizure disorder), ICU admission type (e.g., surgery), and mechanical ventilation
