# Supplementary material for: Delirium‐associated medication in people at risk: A systematic update review, meta‐analyses, and GRADE‐profiles
Source: Acta Psychiatr Scand. 2022 Oct 11;147(1):16–42. doi: 10.1111/acps.13505 (PMC10092229; doi:10.1111/acps.13505)
Supplement: Supplementary file 3 — Appendix S3 Supporting Information. [file ACPS-147-16-s002.docx]

**Appendix – Cochrane risk of bias (RoB) assessment of single RCTs**

1. **RoB table Al-Qadheeb et al.; 2016**

| **Bias** |  | **Outcome / Endpoint** | **Reviewers’ judgment** | **Support for judgment** |
| --- | --- | --- | --- | --- |
| Selection bias | Random sequence generation | - | **Low risk of bias** | Citation: “Subjects were randomized in blocks of four to receive either haloperidol (1 mg IV every 6 hr) or placebo in a 1:1 ratio by means of a computer-generated random number table with treatment allocation known only to the investigational pharmacist” |
|  | Allocation concealment | - | **Low risk of bias** | Citation:   - “Subjects were randomized in blocks of four to receive either haloperidol (1 mg IV every 6 hr) or placebo in a 1:1 ratio by means of a computer-generated random number table with *treatment allocation known only to the investigational pharmacist*” - “Each study dose was prepared by the investigational pharmacy so that an *identical looking* 0.5 mL tuberculin syringe contained 0.2 mL of either haloperidol 1 mg or 5% dextrose in water (D5W).” |
| Performance bias | Blinding of participants and researchers | Intervention group vs. placebo group | **Low risk of bias** | Citation:   - “Subjects, clinicians, and all study personnel were blinded to study drug assignment.” - „Clinicians, investigators, patients, and their families remained blinded to treatment allocation” |
| Detection bias | Blinding of outcome assessment | Blinding to Intervention group vs. placebo group while assessing primary outcome (incident delirium) | **Low risk of bias** | Citation:   - “Subjects, clinicians, and all study personnel were blinded to study drug assignment.” - „Clinicians, investigators, patients, and their families remained blinded to treatment allocation” |
| Attrition bias | Incomplete outcome data | Primary outcome (incident delirium) | **Low risk of bias** | Citation: “No subjects withdrew from the study, and thus, 68 subjects were included in the final analysis.” |
| Reporting bias | Selective reporting | - | **Low risk of bias** | Comment:  No pre-published study protocol found; All primary and secondary outcomes as described in the methods section of the paper as well as on clinicaltrial.gov registration (except of secondary outcome point 6 [cognition, incidence of depressive symptoms, quality of life and sleep quality] were reported in the results section; the missing secondary outcome was not regarded essential for the research question of the review |
| Other bias | - | - | **Low risk of bias** | Comment:  study appears to be free of other sources of bias   - (no potential source of bias related to the specific study design nor evidence of fraudulent behaviour) |

1. **RoB table Avidan et al.; 2017**

| **Bias** |  | **Outcome / Endpoint** | **Reviewers’ judgment** | **Support for judgment** |
| --- | --- | --- | --- | --- |
| Selection bias | Random sequence generation | - | **Low risk of bias** | Citation:   - “Participants were block-randomised by the coordinating centre using *computer-generated randomisation* in blocks of 15 patients. The randomisation codes were sent to participating hospital pharmacists, who assigned study numbers to enrolled patients. Each block of 15 patients contained equal numbers in each group (1:1:1 ratio of 0·5 mg/kg ketamine: 1 mg/kg ketamine: saline placebo) to balance the randomisation across sites and maintain homogeneity between groups.” - “Using a computer-generated randomisation sequence we randomly assigned patients to one of three groups in blocks of 15 to receive placebo (normal saline), low-dose ketamine (0·5 mg/kg), or high dose ketamine (1·0 mg/kg) after induction of anaesthesia, before surgical incision.” |
|  | Allocation concealment | - | **Low risk of bias** | Citation: “Study identifiers were documented in the REDCap database. Prepared formulations of either saline placebo or ketamine were directly delivered to the operating room. *Randomisation codes were concealed until the primary analysis was completed*. Clinicians, patients, and study team members were blinded to the study drug. The study syringes were prepared by pharmacists such that the *contents of the syringes (ketamine vs saline) or ketamine concentration (if they contained ketamine) could not be determined by visual inspection*.” |
| Performance bias | Blinding of participants and researchers | Study medication (Ketamine) or placebo | **Low risk of bias** | Citation: “Clinicians, patients, and study team members were blinded to the study drug. The study syringes were prepared by pharmacists such that the contents of the syringes (ketamine *vs* saline) or ketamine concentration (if they contained ketamine) could not be determined by visual inspection.” |
| Detection bias | Blinding of outcome assessment | Postoperative delirium (primary outcome) | **Low risk of bias** | Citation: “Trained members of the research team who were blinded to group assignment assessed patients for delirium (primary outcome) […]” |
| Attrition bias | Incomplete outcome data | Postoperative delirium (primary outcome) | **Low risk of bias** | Comment: There was missing outcome data for 5 (of 222) patients in the placebo group, 6 (of 227) in the 0.5mg/kg Ketamine group and 7 (of 223) in the 1.0mg/kg Ketamine group; reasons therefore were stated and comparable throughout the groups |
| Reporting bias | Selective reporting | - | **Low risk of bias** | Comment: all primary and secondary outcomes as mentioned in the study protocol as well as in the methods section of the paper were reported in the results section of the paper as well as in the supplementary appendix (online) |
| Other bias | - | - | **Low risk of bias** | Comment:  study appears to be free of other sources of bias   - (no potential source of bias related to the specific study design nor evidence of fraudulent behaviour) |

1. **RoB table Clemmesen et al.; 2018**

| **Bias** |  | **Outcome / Endpoint** | **Reviewers’ judgment** | **Support for judgment** |
| --- | --- | --- | --- | --- |
| Selection bias | Random sequence generation | - | **Low risk of bias** | Citation: “A computer-generated 1:1 randomisation list using the free Internet service www.randomization.com was generated by two nurses not otherwise involved in the study. One-hundred and twenty patients were assigned randomly to one of two equal-sized groups.” |
|  | Allocation concealment | - | **Low risk of bias** | Citation: “Their allocation was concealed in 120 sequentially numbered and opaque envelopes determining active treatment or placebo.” |
| Performance bias | Blinding of participants and researchers | Intervention group vs placebo group | **Low risk of bias** | Citation: “Inclusion triggered two nurses from a separate hospital ward to open a sealed randomisation envelope and prepare the trial medication indicated. Both the methylprednisolone (as methylprednisolone sodium succinate 125 mg/2 ml (Pfizer, Ixelles, Belgium) and the placebo were supplied to the blinded anaesthetist as transparent solutions of the same volume (5 ml) in identical syringes. All trial participants, care providers, attending physicians, nurses, data collectors and analysers were blinded to group allocation.” |
| Detection bias | Blinding of outcome assessment | Intervention group vs placebo group | **Low risk of bias** | Citation: “All trial participants, care providers, attending physicians, nurses, data collectors and analysers were blinded to group allocation” |
| Attrition bias | Incomplete outcome data | Secondary outcome (postoperative delirium) | **Low risk of bias** | Comment: 120 study participants were randomly allocated. One of 60 participants in the Methylprednisolone group withdrew due to withdrawal of consent, two of 60 participants in the placebo group due to interhospital transfer. |
| Reporting bias | Selective reporting | - | **Low risk of bias** | Comment: Study was pre-registered on ClinicalTrials.gov and all primary and secondary outcomes as described in the preregistration as well as in the methods section of the paper were reported in the results section (except of inflammatory biomarkers that do not play a role in the research question of the review) |
| Other bias | - | - | **Low risk of bias** | Comment: study appears to be free of other sources of bias   - (no potential source of bias related to the specific study design nor evidence of fraudulent behaviour) |

1. **RoB table Hongyu et al.; 2019**

| **Bias** |  | **Outcome** | **Reviewers’ judgment** | **Support for judgment** |
| --- | --- | --- | --- | --- |
| Selection bias | Random sequence generation | - | **Unclear risk of bias** | Citation: “The patients were randomized into three groups (n=30 each) on the basis of pre-anesthetic medication: group A (PHC), group B (atropine), and group C (normal saline control) according to **numerical random method**.”  Comment: The process of randomly allocating study participants to study groups was not presented clearly enough to be able to retrace the random sequence generation. Nevertheless, it is unlikely representing a high risk of bias because baseline characteristics of the three groups as presented in table 1,2,3 and 4 are comparable without statistically significant differences. |
|  | Allocation concealment | - | **Unclear risk of bias** | Comment: No information provided about allocation concealment but baseline characteristics across intervention groups are compatible with chance / random intervention group allocation. **#** |
| Performance bias | Blinding of participants and researchers | Primary outcome (incident delirium) | **Unclear risk of bias** | Comment: No information provided on blinding of participants or researchers. There is no evidence / no information that patients were analyzed in a wrong group or that it would affect the outcome. **#** |
| Detection bias | Blinding of outcome assessment | Primary outcome (incident delirium) | **Unclear risk of bias** | Comment: No information provided on blinding of personal who conducted the outcome assessment. Unlikely that possible knowledge of received intervention influenced the outcome assessment. **#** |
| Attrition bias | Incomplete outcome data | Primary outcome (incident delirium) | **Low risk of bias** | Comment: No evidence that attrition occurred. There is no information provided but it seems unlikely that attrition occurred and numbers (table 6) suggest no attrition. |
| Reporting bias | Selective reporting | - | **Unclear risk of bias** | Comment: No study protocol or trial pre-registration was found.  All primary and secondary outcomes as mentioned in the methods section of the paper were presented in the results section. |
| Other bias | - | - | **Low risk of bias** | Comment: study appears to be free of other sources of bias   - (no potential source of bias related to the specific study design nor evidence of fraudulent behaviour) |

Clarification:

**#** items were additionally assed and compared using RoB 2.0 tool from Cochrane. The RoB 2.0 evaluation was “some concerns” in all categories which was regarded as the equivalent of “unclear risk of bias”.

1. **RoB table Kalisvaart et al.; 2005**

| **Bias** |  | **Outcome** | **Reviewers’ judgment** | **Support for judgment** |
| --- | --- | --- | --- | --- |
| Selection bias | Random sequence generation | - | **Low risk of bias** | Citation: “Eligible patients were sequentially randomly assigned to study treatment (placebo or haloperidol 0.5 mg three times daily) from a block of drugs that the hospital pharmacist had prepackaged, according to a **computer-generated randomization code**.” |
|  | Allocation concealment | - | **Low risk of bias** | Citation: “Placebo medication was **identical** in appearance to the active drug” |
| Performance bias | Blinding of participants and researchers | Intervention (Haloperidol) vs. control (placebo) group | **Low risk of bias** | Citation: “**The research team and all participants were blinded to the treatment group**, and **blinding** was **maintained** throughout the study and **checked** by **interviewing** the assessors.”  Comment: Due to emergencies “two  patients in the haloperidol group and five in the placebo group” had to be revealed of their intervention- / control-status. This was not regarded as representing a risk of bias |
| Detection bias | Blinding of outcome assessment | Delirium incidence (primary outcome) | **Low risk of bias** | Citation:   - “Members of the research team not involved in the clinical care of the patients performed all baseline and outcome assessments“ - “**The research team and all participants were blinded to the treatment group**, and **blinding** was **maintained** throughout the study and **checked** by **interviewing** the assessors.”   Comment: Due to emergencies “two  patients in the haloperidol group and five in the placebo group” had to be revealed of their intervention- / control-status. This was not regarded as representing a risk of bias |
| Attrition bias | Incomplete outcome data | Delirium incidence (primary outcome) | **Low risk of bias** | Citation: “The dropout incidence was 20 (9.4%) patients in the haloperidol group, of which 11 were lost for follow-up for the per-protocol analysis and 28 (12.8%) patients in the placebo group, of which 24 were lost to follow-up”  Comment:  Missing outcome data: 11 / 212 (5.2 %; intervention group) and 24 / 218 (11.0 %; control group) were lost to follow-up within the intention-to-treat (ITT) analysis; reasons for drop-outs were comparable within both groups and are unlikely to have a clinically relevant impact on the intervention effect estimate |
| Reporting bias | Selective reporting | - | **Unclear risk of bias** | No study protocol retrieved; unclear whether all predefined primary or secondary outcomes were reported  Primary (incidence of postoperative delirium) and secondary outcomes (severity / duration of delirium and length of hospital stay) were reported in the results section as mentioned in the methods section of the paper |
| Other bias | - | - | **Low risk of bias** | study appears to be free of other sources of bias   - (no potential source of bias related to the specific study design nor evidence of fraudulent behaviour) |

1. **RoB table Larsen et al.; 2010**

| **Bias** |  | **Outcome / Endpoint** | **Reviewers’ judgment** | **Support for judgment** |
| --- | --- | --- | --- | --- |
| Selection bias | Random sequence generation | - | **Low risk of bias** | Citation: “The patients were then randomly assigned to receive either perioperative olanzapine or placebo. The statistician  provided the pharmacy with **a computer-generated random-number table** for participant randomization.”  Comment: Initial stratification into two cohorts according to the complexity of the joint-replacement surgery (simple and complex cohort). Thereafter, randomization with computer-generated random sequence |
|  | Allocation concealment | - | **Low risk of bias** | Citation: “[…] placebo of similar appearance to the olanzapine tablet. The hospital pharmacy prepackaged the study drug and placebo in identical packages […]”  Comment: Identical packages and similar appearance of Olanzapine and placebo in the form of orally-disintegrating tablets |
| Performance bias | Blinding of participants and researchers | Blinding to intervention or placebo group | **Low risk of bias** | Citation:   - “Study patients, attending and consulting staff, nursing staff, research assistants, and investigators remained blinded to study-group assignments throughout the trial.” - “The hospital pharmacy prepackaged the study drug and placebo in identical packages, carried out the randomization, and blinded investigators and subjects.” |
| Detection bias | Blinding of outcome assessment | Primary outcome: incident delirium | **Low risk of bias** | Citation:   - “Study patients, attending and consulting staff, nursing staff, research assistants, and investigators remained blinded to study-group assignments throughout the trial.” - “A research assistant interviewed patients and gathered information from the nursing staff about each patient’s mental status and any signs or symptoms of delirium. Nurses trained in the use of the Confusion Assessment Method (CAM) documented observed manifestations of delirium. Research assistants administered the Mini-Mental State Exam (MMSE) and the Delirium Rating Scale-Revised-98 (DRS-R-98) delirium assessment instruments. A blinded co-investigator (clinical psychologist) collected and verified each day’s data” |
| Attrition bias | Incomplete outcome data | Primary outcome: incident delirium | **Low risk of bias** | Comment: In total 95 out of 495 (19.2%) dropped out after randomization (pre-operatively and before study intervention) and where not included in the final analysis. Reasons therefore were stated and comparable throughout the groups (see figure 1).  Citation: “Attrition occurred from normal discharge from the hospital, with 400 patients remaining in the hospital on Postoperative Day 1 (POD: 1), 398 on POD 2; 318 on POD 3; and 129 on POD 4.”  Normal discharge from hospital was not regarded as causing a risk of attrition bias. |
| Reporting bias | Selective reporting | - | **Unclear risk of bias** | No study protocol retrieved; unclear whether all predefined primary or secondary outcomes were reported  Primary (incidence of postoperative delirium) and secondary outcomes (e.g. the length of stay, medical complications, the severity and duration of delirium, and disposition after hospital discharge.) were reported in the results section as mentioned in the methods section of the paper |
| Other bias | Confounding | Confounding with alcohol withdrawal symptoms | **Unclear risk of bias** | Citation: “Outcomes in our study may have been influenced by the anticholinergic potency of olanzapine as well as the patients who developed unanticipated postoperative alcohol withdrawal during the study. Preoperative misrepresentation of alcohol intake (>10 drinks/week met exclusion criteria) resulted in acute alcohol withdrawal in 5 of the 28 patients (17.9%) who developed delirium in the olanzapine-treated group and 1 of the 82 (1.2%) who developed it in the placebo-treated group. Symptoms of alcohol withdrawal were included in our assessments for delirium.”  Comment: present (and history of) alcohol abuse was an exclusion criterion. However, 17.9% of delirious patients in the Olanzapine group and 1.2% of delirious patients in the placebo group developed alcohol withdrawal symptoms and were included in the outcome assessment of delirium. Although a higher percentage of alcohol withdrawal in the intervention group would reduce the prophylactic effect of olanzapine it remains unclear if alcohol withdrawal symptoms were confounded with delirium and if that could have imposed a bias on the effect measure of olanzapine |

1. **RoB table Perbet et al.; 2018**

| **Bias** |  | **Outcome / Endpoint** | **Reviewers’ judgment** | **Support for judgment** |
| --- | --- | --- | --- | --- |
| Selection bias | Random sequence generation | - | **Low risk of bias** | Citation: “Patients were randomised using computer-generated, permuted block 1:1 randomisation (known only to the investigational pharmacists) to receive ketamine or placebo.” |
|  | Allocation concealment | - | **Low risk of bias** | Comment: Unclear if or which measurements were taken to conceal randomization allocation but baseline characteristics of study groups and randomization process suggest that there is no increased risk of selection bias. |
| Performance bias | Blinding of participants and researchers | Intervention vs placebo group | **Unclear risk of bias** | Comment: The trial is described as double-blind and in the pre-registration of the study it is stated that participants and care providers were masked or blinded. But it remains unclear whether all personal involved in the study was blinded and if blinding was maintained throughout the study (i.a. because there is a non-blinded team mentioned)  Citation: Citations of mentioned blinding in the paper:   - “Patients were randomised using computer-generated, permuted block 1:1 randomisation (known only to the investigational pharmacists) to receive ketamine or placebo.” - “Concentration of ketamine was adapted by a nurse of the **non-blinded team,** according to patient weight, to keep a constant infusion flow” |
| Detection bias | Blinding of outcome assessment | Intervention vs placebo group | **Unclear risk of bias** | Comment: The trial is described as double-blind and in the pre-registration of the study it is stated that participants and care providers were masked or blinded. But it remains unclear whether all personal involved in the study was blinded and if blinding was maintained throughout the study (i.a. because there is a non-blinded team mentioned); unclear if bed-side nurse who assed for delirium was blinded or not  Citation: Citation of mentioned blinding in the paper:   - “Concentration of ketamine was adapted by a nurse of the **non-blinded team**, according to patient weight, to keep a constant infusion flow” |
| Attrition bias | Incomplete outcome data | Secondary outcome (incidence of delirium) | **Low risk of bias** | Comment: The study authors stated no lost to follow up and complete outcome data for all study participants |
| Reporting bias | Selective reporting | - | **Low risk of bias** | Comment: All primary and secondary outcomes as described in the methods section of the paper and in the pre-registration of the trial on clinicaltrials.gov were reported in the results section of the paper |
| Other bias | Confounding of prevalent and incident delirium | - | **Unclear risk of bias** | Citation: “all participants were screened and enrolled after ICU admission and did not have baseline delirium and cognitive assessments. Therefore, we cannot preclude the potential bias introduced by pre-admission imbalance of baseline conditions. However, relatives’ interrogations before randomization and large sample size should have helped to balance these factors between groups.”  Comment: It remains unclear whether the lack of baseline assessment of cognitive and delirium assessment could have caused confounding of prevalent and incident delirium, therefore review authors judged this as an unclear risk of bias. |

1. **RoB table Sauer et al.; 2014**

| **Bias** |  | **Outcome / Endpoint** | **Reviewers’ judgment** | **Support for judgment** |
| --- | --- | --- | --- | --- |
| Selection bias | Random sequence generation | - | **Low risk of bias** | Citation: “An independent statistician created a computer-generated 1:1 randomization scheme, which was stratified to participating center and in blocks of 40.” |
|  | Allocation concealment | - | **Low risk of bias** | Citation: “Packages and ampoules of dexamethasone and placebo were identical and contained an equal volume (5mL) of a 20mg/mL dexamethasone solution or normal saline, respectively” |
| Performance bias | Blinding of participants and researchers | Intervention vs placebo group | **Low risk of bias** | Citation:   - “Patients, caregivers, and researchers were unaware of study group assignment” - “A strength of our study is that blinding for treatment was well maintained during the perioperative period. “ |
| Detection bias | Blinding of outcome assessment | Intervention vs placebo group | **Low risk of bias** | Citation: “Patients, caregivers, and researchers were unaware of study group assignment” |
| Attrition bias | Incomplete outcome data | Primary outcome (postoperative delirium incidence) | **Low risk of bias** | Comment: A total of 49 (of 367; 13.4%) study participants in the dexamethasone group vs a total of 45 (of 370; 12.2%) in the placebo group dropped out due to comparable and relatively comparable reasons (with the most frequent reason being transfers); In a sensitivity analysis the study authors showed that assigning all participants who died in the 4 postoperative days to delirium did not change the results |
| Reporting bias | Selective reporting | - | **Unclear risk of bias** | Comment: All primary and secondary outcomes as described in the methods section of the paper (as well as in the original DECS study) were reported in the results section of the paper. No pre-published study protocol was found. Registration on clinicaltrials.gov does not describe pre-defined outcomes |
| Other bias | - | - | **Low risk of bias** | Comment:  study appears to be free of other sources of bias   - (no potential source of bias related to the specific study design nor evidence of fraudulent behaviour) |

1. **RoB table Schrijver et al.; 2018**

| **Bias** |  | **Outcome / Endpoint** | **Reviewers’ judgment** | **Support for judgment** |
| --- | --- | --- | --- | --- |
| Selection bias | Random sequence generation | - | **Low risk of bias** | Citation:   - “‘Apotheek Haagse Ziekenhuizen’ was responsible for manufacturing, processing and packaging the verum and placebo haloperidol tablets in accordance with *Good Manufacturing Practice guidelines* and provided the randomisation schemes and blinding.” - “Treatment assignment was performed in a blind manner using fixed randomisation schemes per site with a block size of 4 (1:1) stratified according to wellknown risk factors for hospital-associated delirium (age 70–79 or ≥80 years; planned surgery on admission or not).“ - “To balance population representatives in both treatment groups, four strata are constructed with separate randomisation schedules: (1) age 70 to 80 years, planned surgery on admission; (2) age 70 to 80 years, no planned surgery on admission; (3) age 80 years or over, planned surgery on admission; (4) age 80 years or over, no planned surgery on admission. Each stratum consists of 20 pre-randomised individual study numbers, 10 assigned to haloperidol and 10 to placebo treatment…” (study protocol) |
|  | Allocation concealment | - | **Low risk of bias** | Citation: “Placebo and haloperidol tablets were identical in appearance and packaged in identical unit-dose blister packs in cardboard boxes with sequentially numbered labels, each with an unique study identification number.” |
| Performance bias | Blinding of participants and researchers | Primary outcome (Incident delirium) | **Low risk of bias** | Citation:   - “Participants, observers, physicians, nurses, other care givers, at the investigative sites are all blinded to the assigned intervention. The VUmc clinical pharmacy holds the randomisation list.” (study protocol) - “Emergency unblinding was possible by 24 h contact with an on-call pharmacist in case delirium developed or when knowledge of treatment assignment could influence patient care. Study staff, clinicians and participants were to remain blinded throughout the study.” |
| Detection bias | Blinding of outcome assessment | Primary outcome (Incident delirium) | **Low risk of bias** | Citation:   - “Participants, observers, physicians, nurses, other care givers, at the investigative sites are all blinded to the assigned intervention. The VUmc clinical pharmacy holds the randomisation list.” (study protocol) - “Data will be mainly collected by two observers, a physician (EJMS) and geriatric nurse (AV) trained according to the Good Clinical Practice (GCP) principles.” (study protocol) - “Nursing records included three times daily care observations and the 13-item Delirium Observation Screening Scale (DOSS) scores [21], with average daily scores of ≥3 indicating possible delirium. Delirium diagnosis was confirmed by a clinician or an investigator according to Diagnostic and Statistical Manual of Mental Disorders, 4th edition (DSM-IV) criteria for delirium [22]. All delirium cases were retrospectively verified by the local principal investigator by reviewing patient records before unblinding.”   Comment: Blinding is described as having been maintained throughout the study and with regard to outcome assessment |
| Attrition bias | Incomplete outcome data | Primary outcome (incident delirium) | **Low risk of bias** | Comment: Intention to treat (ITT) analysis; 1 patient in the Haloperidol group and 2 patients in the placebo group could not be included in the ITT analysis due to missing informed consent. This was not regarded as presenting a risk of bias.  (6/118 patients in the Haloperidol group did not receive the study drug vs. 7/124 in the placebo group due to comparable reasons that is not considered as changing the comparability of randomized groups) |
| Reporting bias | Selective reporting | - | **Low risk of bias** | Comment: All primary and secondary outcomes as described in the pre-published study protocol as well as in the methods section of the published paper were described in the results section of the study  (“delirium free days” was not – to be precise – but it is not considered relevant to the research question of the review) |
| Other bias | - | - | **Low risk of bias** | Comment: study appears to be free of other sources of bias   - (no potential source of bias related to the specific study design nor evidence of fraudulent behaviour) |

1. **RoB table Van den Boogaard et al.; 2018**

| **Bias** |  | **Outcome / Endpoint** | **Reviewers’ judgment** | **Support for judgment** |
| --- | --- | --- | --- | --- |
| Selection bias | Random sequence generation | - | **Low risk of bias** | Citation: “Randomization was applied by the pharmacist of the Radboudumc using a permuted block randomization. Patients were allocated to each group in a 1:1:1 ratio. The pharmacist who kept the randomization code and the members of the data and safety management board were the only people who were unblinded for this study” |
|  | Allocation concealment | - | **Low risk of bias** | Citation:   - “The study medication was accompanied with a randomization list. Following randomization, the numbers printed on the medication box were coupled with the randomization numbers. The numbered boxes consisted of 12ampoules of the study drug. If necessary, when a patient was admitted to the ICU for more than 4 days and did not develop delirium, a follow-up study medication box was assigned to this patient consisting of the same study regime as the previous box. The follow up study medication was always delivered by a researcher or pharmacist who was not involved in the study, using a shadow list with the group code to ensure that the patients remained in the same study group.” - “All study medication was prepared by the pharmacy department at Radboudumc according to good manufacturing practice regulations. All ampoules of the study medication had a total volume of 1 mL. The ampoules and drug boxes were identical in appearance and in labelling.” |
| Performance bias | Blinding of participants and researchers | Secondary outcome: incident delirium | **Low risk of bias** | Citation: “The physicians, nurses,  investigators and participating patients will be blinded for treatment allocation.” (study protocol) |
| Detection bias | Blinding of outcome assessment | Secondary outcome: incident delirium | **Low risk of bias** | Citation: “The physicians, nurses,  investigators and participating patients will be blinded for treatment allocation.” (study protocol) |
| Attrition bias | Incomplete outcome data | Secondary outcome: incident delirium | **Low risk of bias** | Citation:   - “Patients of which informed consent was obtained and who received at least one dose of study drug will remain in the study” (study protocol) - “All patients who did not receive the study medication according to the study protocol (6.8% in 2-mg haloperidol group and 5.5% in the placebo group) were excluded from the per-protocol analysis.” - “Five serious adverse events were reported. Three patients died, 1 in each of the 3 groups (Table 2). None of the serious adverse events were likely related to the study medication. (…) The number of reported adverse events was not statistically different between groups.”   Comment: Intention to treat analysis was conducted primarily. A per protocol analysis was performed as well. Both analyses do not show statistically significant measures or differences. A drop-out rate in the per-protocol analysis of 6.8% (Haloperidol group) and 5.5% (placebo group) was considered being low and comparable among the groups by the review authors. It is not considered as having a clinically relevant impact on the effect measure. |
| Reporting bias | Selective reporting | - | **Low risk of bias** | Comment: all primary and secondary endpoints as described in the study protocol and in the methods section of the published article were reported in the results section of the article (except of long-term quality of life. It is mentioned that it will be reported “elsewhere”.)  This one missing secondary endpoint is not considered to bias (the main) results according to the research question of the review |
| Other bias | - | - | **Low risk of bias** | study appears to be free of other sources of bias   - (no potential source of bias related to the specific study design nor evidence of fraudulent behaviour) |

1. **RoB table Wang et al.; 2012**

| **Bias** |  | **Outcome / Endpoint** | **Reviewers’ judgment** | **Support for judgment** |
| --- | --- | --- | --- | --- |
| Selection bias | Random sequence generation | - | **Low risk of bias** | Citation: “Randomization was stratified by center. Eligible patients were sequentially randomly assigned to either haloperidol group or placebo group according to computer-generated randomization codes.” |
|  | Allocation concealment | - | **Low risk of bias** | Citation: “Study drug was prepared by an independent nurse with either haloperidol (5 mg diluted with normal saline to a final concentration of 0.1 mg/mL) or placebo (normal saline), and was prepackaged according to the randomization code. Placebo medication was identical in the appearance to the active drug.” |
| Performance bias | Blinding of participants and researchers | Primary outcome: incident postoperative delirium | **Low risk of bias** | Citation:   - “All study personnel, healthcare team members, and patients were unaware of treatment group assignment, and blinding was maintained throughout the whole study period. Code envelopes were stored at the site of investigation. In case of emergency, attending intensivist for patient care could request unmasking of the treatment allocation. A statement had to be made in the case report formulary in that case.” - “no emergent unmasking of the treatment allocation was needed in all enrolled patients.“ (results section) |
| Detection bias | Blinding of outcome assessment | Primary outcome: incident postoperative delirium | **Low risk of bias** | Citation:   - „Outcome assessments were performed by research members who were not involved in clinical care of patients.“ - “All study personnel, healthcare team members, and patients were unaware of treatment group assignment, and blinding was maintained throughout the whole study period. Code envelopes were stored at the site of investigation. In case of emergency, attending intensivist for patient care could request unmasking of the treatment allocation. A statement had to be made in the case report formulary in that case.” - “no emergent unmasking of the treatment allocation was needed in all enrolled patients.“ (results section) |
| Attrition bias | Incomplete outcome data | Primary outcome: incident postoperative delirium | **Low risk of bias** | Citation: “Among them, 457 patients gave written informed consents and were randomized into the study. Study drug infusion was failed to initiate in four patients (three in the haloperidol group and one in the placebo group) because of heavy clinical workload. The results of these four patients were included in the final intention-to-treat analyses.”  Comment: Very low drop out / attrition which was not considered as having an impact on the structure of groups or biasing the results |
| Reporting bias | Selective reporting | - | **Unclear risk of bias** | Comment: no pre-published study protocol retrieved; all primary and secondary outcomes as described in the methods section of the article were reported in the results section |
| Other bias | Significant intraoperative differences between groups | - | **Unclear risk of bias** | Citation: “Baseline characteristics were similar between the two groups (Table 1). Despite double-blind randomization, the **durations of anesthesia and surgery** were significantly longer, and the **volume of total intraoperative infusion** was *significantly larger in the haloperidol group than in the placebo group*. There were no significant differences with regard to other intraoperative and postoperative variables between the two groups (Table 2).”  Comment: Unclear whether randomization failed to provide similar intraoperative characteristics or other causes leaded to two statistically significant differences in duration of anesthesia and surgery and volume of total intraoperative infusion. Both characteristics can be regarded as risk factors for postoperative delirium but it remains unclear whether it caused a risk of bias of the effect measure. |
