# Supplementary material for: Delirium‐associated medication in people at risk: A systematic update review, meta‐analyses, and GRADE‐profiles
Source: Acta Psychiatr Scand. 2022 Oct 11;147(1):16–42. doi: 10.1111/acps.13505 (PMC10092229; doi:10.1111/acps.13505)

**Appendix - search strategy & eligibility criteria**

1. **TABLES**
   1. Eligibility criteria

Table 1: eligibility criteria for inclusion and exclusion of retrieved studies

|  | **Inclusion criteria** | **Exclusion criteria** |
| --- | --- | --- |
| Study design | - randomized controlled trials (RCT), prospective observational studies (cohort studies, nested case-control studies) | - case reports, case series, editorials, letters, conference abstracts, (systematic) reviews, meta-analyses - retrospective study design - observational studies without exploratory statistics or data analysis |
| Population | - adults (> 18 years) | - study participants ≤ 18 years |
| Exposure | - exposure to drugs suspected to induce delirium | - clinical evaluation of purely anticholinergic drug scores or scales |
| Control | - non exposure | - no control group |
| Outcome | - incident delirium:   all types (hypoactive, hyperactive, mixed; post-operative, withdrawal of drugs / substances, DSD [delirium superimposed on dementia]) | - impact on the duration of prevalent delirium |
| - Outcome assessment | - diagnostic criteria of DSM (all versions), ICD (all versions) or assessment instruments validated against DSM or ICD (e.g. CAM) - clinical diagnosis | - no precise disclosure of diagnostical assessment |
| Setting | - institutional settings (all medical and surgical specialities, intensive care unit [ICU], post anesthesia care unit, palliative setting, geriatrics, nursing facilities) | - pediatrics |
| Publication language | - German, English, French | - all others |
| Time period of studies | - since October 2009 | - before October 2009 |

- 1. Search terms and databases

Databases and time frame of search:

- MEDLINE (via PubMed): Oct 1, 2009, to June 23, 2020
- EMBASE: Oct 1, 2009, to June 23, 2020
- PsycINFO: Oct 1, 2009, to June 23, 2020
- Google Scholar: no time frame, screening of the first 100 articles, sorted by relevance

Table 2: search terms (including MeSH terms) used

| **Search terms used** |  |
| --- | --- |
| i. dependent variable (delirium) | *delirium [MeSH Terms]* OR *delirium/chemically induced [MeSH Terms]* OR *delir** OR *acute confusional state* OR *acute brain failure* OR *brain dysfunction* |
| ii. independent variable (medication exposure) | *medication** OR *drug** OR *drug induced* OR *adverse effect / event* OR *polypharmacy* OR *anticholinergic** OR *cholinergic receptor blocking agent [MeSH Terms]* OR *dopaminergic** OR *dopamine receptor stimulating agent [MeSH Terms]* OR *delirogenic OR adverse event [MeSH Terms] OR adverse effect* |
| iii. elderly study population | *aged [MeSH Terms]* OR *age** OR *aged hospital patient* OR *elderly* |

1. **FIGURES – search strategy used in databases**

Figure 1: search strategy for MEDLINE (via pubmed)


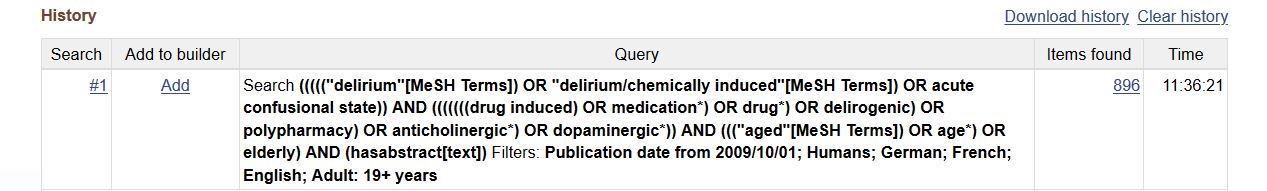


Figure 2: search strategy for Embase


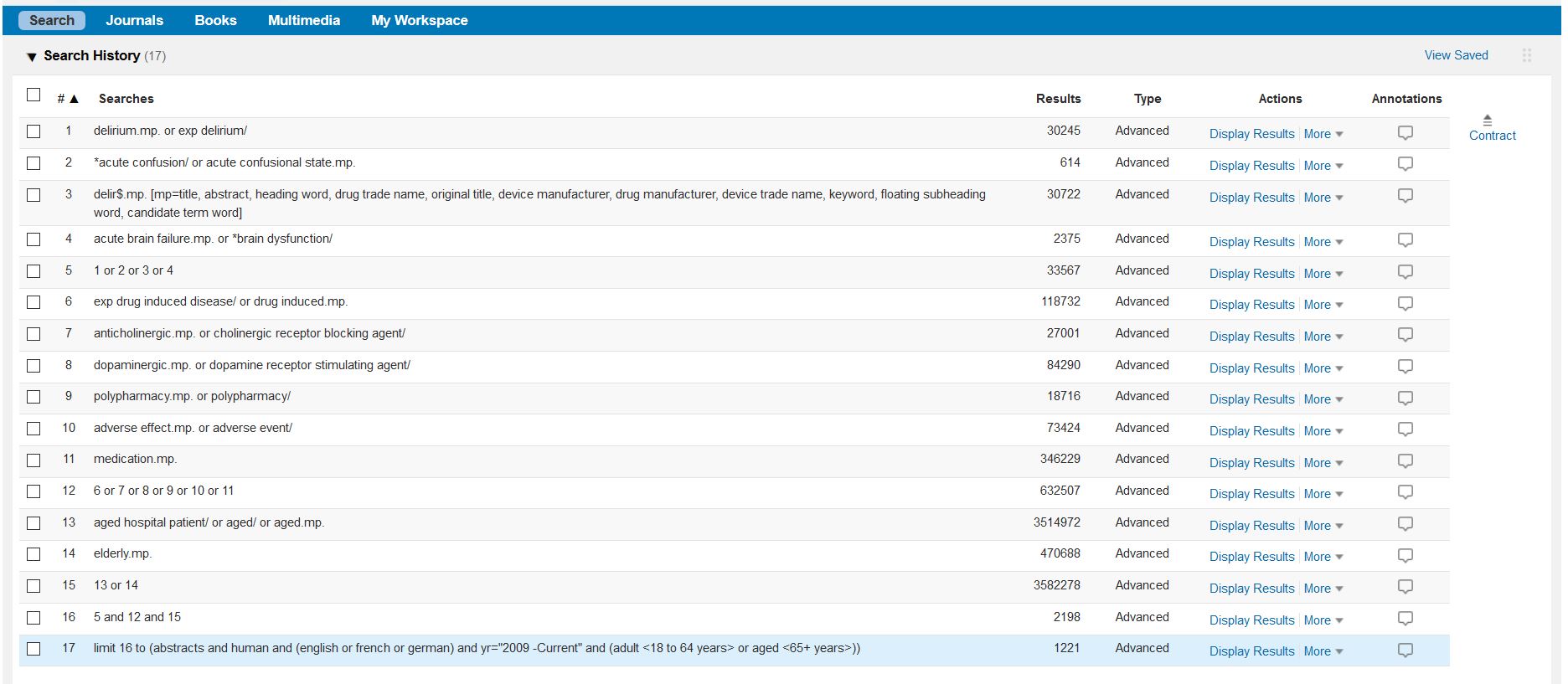


Figure 3: search strategy for PsycInfo


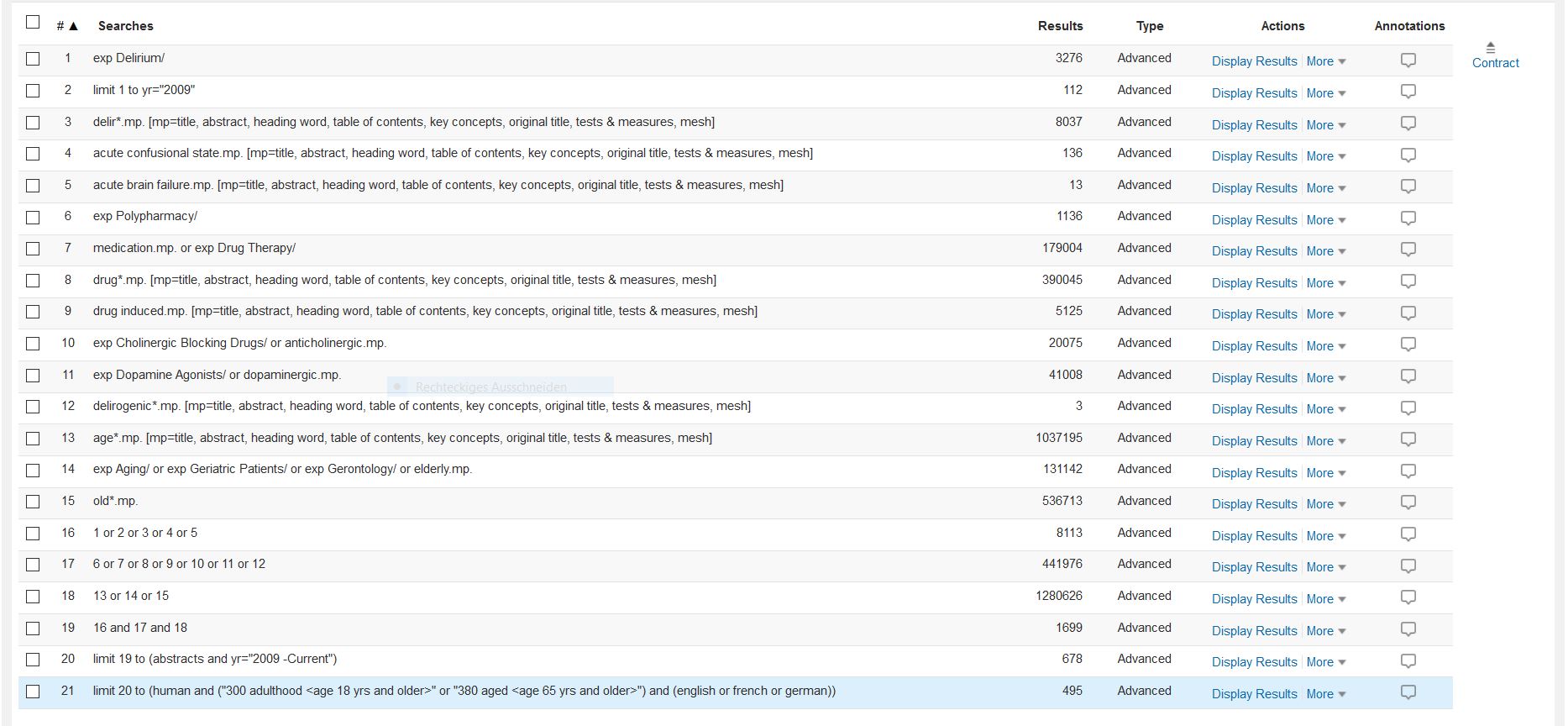

Supplement: Supplementary file 4 — Appendix S4 Supporting Information. [file ACPS-147-16-s001.docx]
